# Global Burden of Respiratory Diseases Attributable to Ambient Particulate Matter Pollution: Findings From the Global Burden of Disease Study 2019

**DOI:** 10.3389/fpubh.2021.740800

**Published:** 2021-11-23

**Authors:** Ying Wu, Ping Song, Shuai Lin, Ling Peng, Yizhen Li, Yujiao Deng, Xinyue Deng, Weiyang Lou, Si Yang, Yi Zheng, Dong Xiang, Jingjing Hu, Yuyao Zhu, Meng Wang, Zhen Zhai, Dai Zhang, Zhijun Dai, Jie Gao

**Affiliations:** ^1^Department of Nephrology, The Second Affiliated Hospital of Xi'an Jiaotong University, Xi'an, China; ^2^Department of Gastroenterology, The Second Affiliated Hospital of Xi'an Jiaotong University, Xi'an, China; ^3^Department of Oncology, The Second Affiliated Hospital of Xi'an Jiaotong University, Xi'an, China; ^4^Department of Respiratory Disease, Zhejiang Provincial People's Hospital, Hangzhou, China; ^5^Department of Breast Surgery, The First Affiliated Hospital, College of Medicine, Zhejiang University, Hangzhou, China; ^6^Celilo Cancer Center, Oregon Health Science Center Affiliated Mid-Columbia Medical Center, The Dalles, OR, United States; ^7^Dana-Farber Cancer Institute and Harvard Medical School, Boston, MA, United States

**Keywords:** ambient particulate matter pollution, global burden of respiratory diseases, death, spatiotemporal trends, disability-adjusted life year

## Abstract

**Background:** Exposure to ambient particulate matter pollution (APMP) is a global health issue that directly affects the human respiratory system. Thus, we estimated the spatiotemporal trends in the burden of APMP-related respiratory diseases from 1990 to 2019.

**Methods:** Based on the Global Burden of Disease Study 2019, data on the burden of APMP-related respiratory diseases were analyzed by age, sex, cause, and location. Joinpoint regression analysis was used to analyze the temporal trends in the burden of different respiratory diseases over the 30 years.

**Results:** Globally, in 2019, APMP contributed the most to chronic obstructive pulmonary disease (COPD), with 695.1 thousand deaths and 15.4 million disability-adjusted life years (DALYs); however, the corresponding age-standardized death and DALY rates declined from 1990 to 2019. Similarly, although age-standardized death and DALY rates since 1990 decreased by 24% and 40%, respectively, lower respiratory infections (LRIs) still had the second highest number of deaths and DALYs attributable to APMP. This was followed by tracheal, bronchus, and lung (TBL) cancer, which showed increased age-standardized death and DALY rates during the past 30 years and reached 3.78 deaths per 100,000 persons and 84.22 DALYs per 100,000 persons in 2019. Among children aged < 5 years, LRIs had a huge burden attributable to APMP, whereas for older people, COPD was the leading cause of death and DALYs attributable to APMP. The APMP-related burdens of LRIs and COPD were relatively higher among countries with low and low-middle socio-demographic index (SDI), while countries with high-middle SDI showed the highest burden of TBL cancer attributable to APMP.

**Conclusions:** APMP contributed substantially to the global burden of respiratory diseases, posing a significant threat to human health. Effective actions aimed at air pollution can potentially avoid an increase in the PM_2.5_-associated disease burden, especially in highly polluted areas.

## Introduction

Ambient particulate matter pollution (APMP) is a leading risk factor for poor health and is strongly associated with cardiovascular, respiratory, and neurovascular diseases (1–3). In the Global Burden of Disease Study in 2019 (GBD 2019), APMP, which refers to particles with an aerodynamic diameter equal to or < 2.5 μm (PM_2.5_), ranks seventh among all the health risks identified (4). In 2013, PM_2.5_, a carcinogen, was recognized by the International Agency for Research on Cancer (5).

Air pollution refers to a complex mixture of gases and different particles in the air. PM_2.5_ in the air significantly contributes to the public health impact of ambient air pollution (6, 7). Due to its small size, PM_2.5_ can enter the human brain and lungs, leading to a variety of disorders, including those of the respiratory system (8). A meta-analysis revealed that ambient PM_2.5_ is associated with lower and upper respiratory infections, and chronic respiratory diseases, such as chronic obstructive pulmonary disease (COPD) and lung cancer (9). It may induce airway inflammation and aggravate wheezing symptoms in patients with COPD (10). Meanwhile, each 10 μg/m^3^ increase in PM_2.5_ concentration has been linked to a 9% increase in the risk of death from lung cancer (11). Additionally, studies have suggested the short- and long-term effects of PM_2.5_ on pneumonia (12, 13). A positive correlation was also found between PM_2.5_ concentration and the case fatality rate of coronavirus disease 2019 (14).

Given the substantial influence of ambient PM_2.5_ on human health, a comprehensive study is needed to provide a broad, global analysis. Thus, using the latest data and improved methods in the GBD 2019, we analyzed the burden of respiratory diseases attributable to APMP to provide a better understanding of the impact of APMP on human health, identify the highly affected areas, and contribute to the global and national action toward reducing the hazardous effects of air pollution.

## Methods

### Overview

Data on the global death and disease burden attributable to APMP were obtained from the GBD 2019, which provides worldwide and comprehensive assessments of health loss for 329 diseases (4). GBD studies included 204 countries and territories that were classified into 21 regions according to epidemiological similarities and geographical proximity, and into five groups based on sociodemographic index (SDI; low, low-middle, middle, high-middle, and high SDI), which is estimated based on economic growth, fertility rate, and educational attainment. The number of deaths, disability-adjusted life years (DALYs), and corresponding age-standardized rates (ASRs) attributed to APMP from 1990 to 2019 were extracted from the GBD 2019 and analyzed by age, sex, location, and respiratory causes. All data in this analysis are available from the GBD query tool (15).

### Estimation of Exposure to APMP and Its Attributable Disease Burden

The GBD studies included the risk-outcome pairs that met the criteria of the World Cancer Research Fund that was convincing or probable evidence. Convincing evidence includes biologically consistent associations between respiratory diseases and APMP reported in previous high-quality studies, while probable evidence refers to epidemiological studies that had insufficient data or other limitations (4). Respiratory diseases identified to be associated with exposure to APMP include COPD, lower respiratory infections (LRIs), upper respiratory infections (URIs), and tracheal, bronchial, and lung (TBL) cancer. Relative risks for each cause of death were estimated through integrated exposure-response functions based on the risk of death from APMP, as previously reported (4). In GBD 2019, a uniform distribution of the theoretical minimum risk exposure level between 2.4 and 5.9 μg/m^3^ for APMP represents the risk level that minimizes risk at the population level, which also indicates the risk level that captures the maximum attributable burden (4). Summary exposure values (SEVs) reported in the GBD 2019 were used to compare exposure to different risks. SEVs ranged from 0 to 100%, representing no population exposure to the risk or all population exposure to the highest level of risk (4). In GBD studies, the attribution of deaths and DALYs of each cause to APMP was calculated by applying the population-attributable fraction for age, sex, location, and year. More details of exposure to APMP and estimation of disease burden have been provided in a previous study (4).

### Joinpoint Regression Analysis

The joinpoint regression model, a set of statistically linear models, was used to evaluate the temporal trends in the ASRs of DALY and death. Changes in trends were described by connecting several different line segments on a logarithmic scale at the “joinpoints” and identifying points where the slope of the linear trend changed significantly over time. Best-fitting points representing statistically significant changes occurring in trends were identified by calculating the square sum of the residual error between the estimated and actual values (16).

### Statistical Analyses

We analyzed all the attributable burdens by age, sex, year, location, and cause to determine the impact of APMP on respiratory diseases. When comparing different populations, and even the same population in different periods, we used the ASRs of deaths and DALYs to eliminate the effects of differences in population structures. The associations between SDI and SEVs, attributable deaths, and DALYs were also analyzed to reveal the association between social development and disease burden. All cases and their corresponding ASRs per 100,000 persons were reported with 95% uncertainty intervals (UIs). The R program (R Core Team, version 3.5.2, Vienna, Austria) was used to perform all of the above analyses.

Joinpoint regression analysis was conducted using the Joinpoint software (version 4.7.0) from the Surveillance Research Program of the US National Cancer Institute. The annual percentage changes (APCs) and their 95% confidence intervals were also calculated. The *p*-value was estimated using a Monte Carlo permutation test and corrected using Bonferroni's analysis, with a significance level of 0.05.

## Results

### Global Burden of Respiratory Diseases Attributable to APMP in 2019

From 1990 to 2019, global exposure to APMP increased by 67.47% in countries in the low-middle SDI quintile, showing the highest increase, while countries with middle SDI had the highest exposure to APMP in 2019 (Supplementary Table 1). As such, the data revealed an increasing disease burden attributable to APMP worldwide. Globally, 4.1 million (95% UI: 3.5–4.8) deaths and 118.2 million (959.5–138.4) DALYs of different diseases were attributed to APMP in 2019, more than twice the number in 1990 (Table 1). Respiratory diseases contributed to 32.10% of deaths and 30.33% of DALYs attributable to APMP exposure. COPD, LRIs, and TBL cancer were among the top five leading causes of death attributable to APMP (Supplementary Figure 1). In 2019, among respiratory diseases, COPD accounted for the largest proportion, with 52.30% [695,071 (553,830–861,855)] and 43.0% [15.4 million (12.4–19.0)] of deaths and DALYs attributable to APMP, respectively. LRIs ranked second, contributing to 24.6% [326,353 (229,567–436,449)] deaths and 37.4% [13.4 million (9.2–18.3)] DALYs attributable to APMP, followed by TBL cancer, which showed a similar number of deaths as LRIs [307,681 (226,684–395,668)] but half the DALYs [7.0 million (5.2–9.0)] attributable to APMP. URIs showed the fewest APMP-attributable deaths and DALYs in 2019 [deaths: 7.8 (4.4–11.9); DALYs: 1,363 (884–1,962)] (Table 1).

**Table 1:** Global burden of respiratory diseases attributable to ambient fine particulate matter pollution in 1990 and 2019, and percent changes from 1990 to 2019.

	**Deaths, in thousands (95% UI)**			**Age-standardized death rate per 100,000 populations (95% UI)**			**DALYs, in thousands (95% UI)**			**Age-standardized DALY rate per 100,000 populations (95% UI)**		
	**1990**	**2019**	**1990–2019, Change (%)**	**1990**	**2019**	**1990–2019, Change (%)**	**1990**	**2019**	**1990–2019, Change (%)**	**1990**	**2019**	**1990-2019, Change (%)**
All causes	2047.17 (1454.74–2739.85)	4140.97 (3454.41–4800.29)	102.28	53.15(38.01–70.48)	52.67(43.99–61.13)	−0.92	70478.35 (47284.19–98903.72)	118215.37 (95948.20–138428.61)	67.73	1500.34 (1035.89–2063.41)	1504.44 (1215.20–1767.33)	0.27
**Respiratory diseases**												
Chronic obstructive pulmonary disease	351.73 (220.23–514.84)	695.07 (553.83–861.86)	97.61	10.19 (6.42–14.89)	8.95 (7.14–11.10)	−12.15	7982.47 (5057.85–11666.25)	15413.75 (12394.37–18965.15)	93.09	209.29 (133.30–305.04)	190.79 (153.52–234.76)	−8.84
Lower respiratory infections	290.36 (173.20–463.10)	326.35 (229.57–436.45)	12.40	5.85 (3.60–9.05)	4.46 (3.13–5.95)	−23.78	19463.72 (11009.97–32484.82)	13418.02 (9211.57–18297.83)	−31.06	321.70 (184.41–529.72)	191.42 (130.56–262.85)	−40.50
Upper respiratory infections	0.03 (0.01–0.06)	0.01 (0.00–0.01)	−74.12	0.00 (0.00–0.00)	0.00 (0.00–0.00)	−73.84	3.09 (1.31–6.01)	1.36 (0.88–1.96)	−55.92	0.05 (0.02–0.09)	0.02 (0.01–0.03)	−55.44
Tracheal, bronchus, and lung cancer	118.44 (79.19–163.89)	307.68 (226.68–395.67)	159.79	3.03 (2.03–4.18)	3.78 (2.79–4.86)	24.59	3011.08 (2013.12–4154.52)	7015.80 (5176.47–9024.57)	133.00	73.16 (48.94–101.24)	84.22 (62.13–108.30)	15.12

*DALYs, disability-adjusted life years; UI, uncertainty interval*.

### Age- and Sex-Specific Burden of Respiratory Diseases Attributable to APMP in 2019

**Figure 1:**
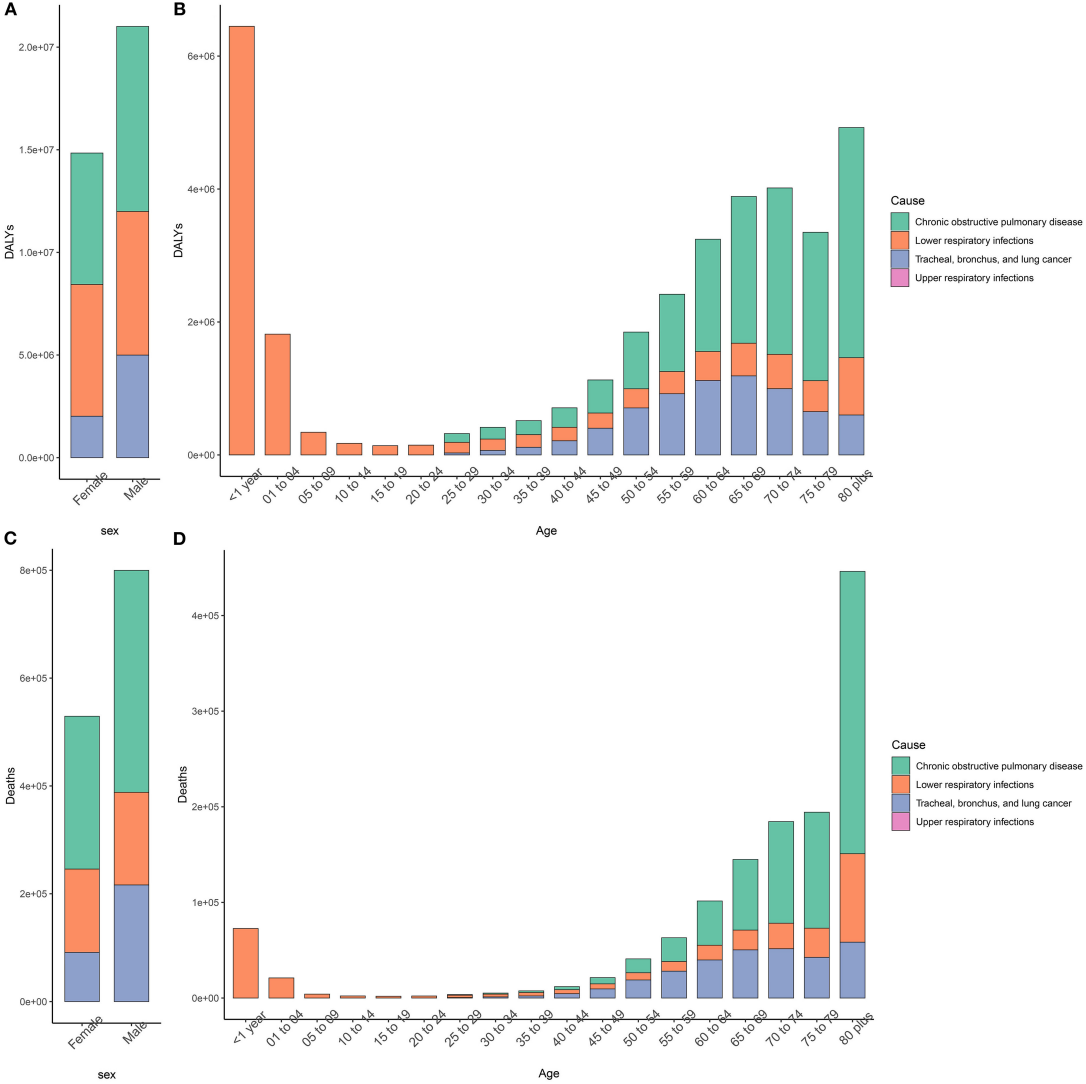
Sex- and age-specific burden of respiratory disease attributable to ambient particulate matter pollution in 2019. **(A,B)**. DALYs; **(C,D)**. Deaths. DALY, disability-adjusted life year.

Figure 1 shows the burden of respiratory diseases attributable to APMP among the sexes and age groups. In terms of sex, the APMP-related deaths and DALYs of respiratory diseases were slightly higher in men than in women. However, for both sexes, COPD was the main respiratory disease that caused an increasing burden attributable to APMP with age and contributed the most to the number of deaths among people aged > 80 years. Similarly, the APMP-related burden of TBL cancer increased with age and peaked in the 65–69 year's age group for deaths and 70–74 age group for DALYs. For those aged < 24 years, LRI was the main respiratory cause that affected their health, leading to infants becoming the main group with the second-highest number of deaths and DALYs of LRIs attributable to APMP (Figure 1, Supplementary Table 2). URI was another contributor for infants attributable to APMP, but the deaths and DALYs of URIs attributable to APMP were extremely low compared with those of LRIs among all ages (Figure 1, Supplementary Table 2).

### Global Trends of Respiratory Diseases Burden Attributable to APMP

**Figure 2:**
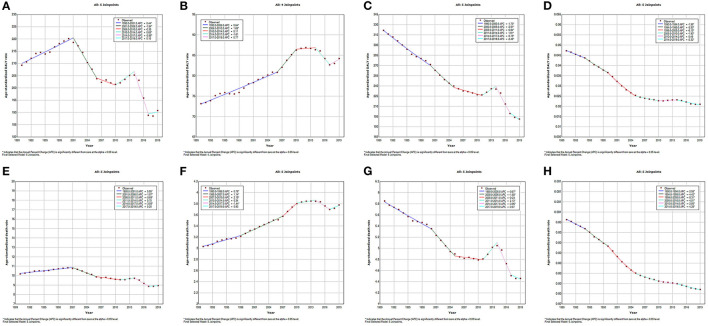
Temporal trend of global age-standardized burden in respiratory diseases attributable to ambient particulate matter pollution from 1990 to 2019. **(A)**. age-standardized DALY rate of COPD; **(B)**. age-standardized DALY rate of TBL cancer; **(C)**. age-standardized DALY rate of LRIs; **(D)**. age-standardized DALY rate of URIs; **(E)**. age-standardized death rate of COPD; **(F)**. age-standardized death rate of TBL cancer; **(G)**. age-standardized death rate of LRIs; **(H)**. age-standardized death rate of URIs. DALY, disability-adjusted life year; COPD, chronic obstructive pulmonary disease; TBL, cancer tracheal, bronchus, and lung cancer; LRIs, lower respiratory infections; URIs, upper respiratory infections.

Temporal trends in respiratory disease burden attributable to APMP globally over the past three decades were revealed by joinpoint analysis (Figure 2). For COPD, the age-standardized DALY rate attributable to APMP decreased by 9% since 1990 [from 209.29 per 100,000 persons (133.30 to 305.04) in 1990 to 190.79 per 100,000 persons (153.52 to 234.76) in 2019], and had two significantly rapid declines (APCs: 2001–2006: by 1.54%, *p* < 0.05; 2014–2017: by 2.86%, *p* < 0.05), although it increased slightly in the other two periods (APCs: 1990–2001: by 0.44%, *p* < 0.05; 2010–2014: by 0.68%, *p* < 0.05) (Figure 2A). Similarly, the age-standardized death rate (ASDR) of COPD attributable to APMP decreased from 10.19 per 100,000 persons in 1990 to 8.95 per 100,000 persons in 2019, with obvious declines in three periods (APCs: 2001–2006: by 1.87%, *p* < 0.05; 2006–2010: by 0.69%, *p* < 0.05; 2014–2017: by 3.08%, *p* < 0.05) (Figure 2E). Except for one period of increasing trend (APCs: 2011–2014: by 1.91%, *p* < 0.05), the age-standardized DALY rate of LRIs attributable to APMP decreased markedly from 321.7 per 100,000 persons in 1990 to 191.4 per 100,000 persons in 2019 with different APCs (Figure 2C). We also found similar trends in the ASDR of LRIs attributable to APMP (Figure 2G). For URIs, although the APMP-related age-standardized DALY and death rates were low globally, declining patterns were observed in the past three decades (Figures 2D,H). In contrast, the age-standardized DALY rate of TBL cancer saw two periods of significant increases attributable to APMP exposure (APCs: 1990–2006: by 0.64%, *p* < 0.05; 2006–2010: by 1.69%, *p* < 0.05), and increased from 2017 to 2019, reaching 84.22 DALYs per 100,000 persons (62.13 108.30) in 2019 (Figure 2B). Similarly, the ASDR of TBL cancer increased with different APCs from 1990 to 2010 and saw one period of decline between 2014 and 2017 (APCs: 1.44%, *p* < 0.05) (Figure 2F).

### Regional and National Burden of Respiratory Diseases Attributable to APMP

**Figure 3:**
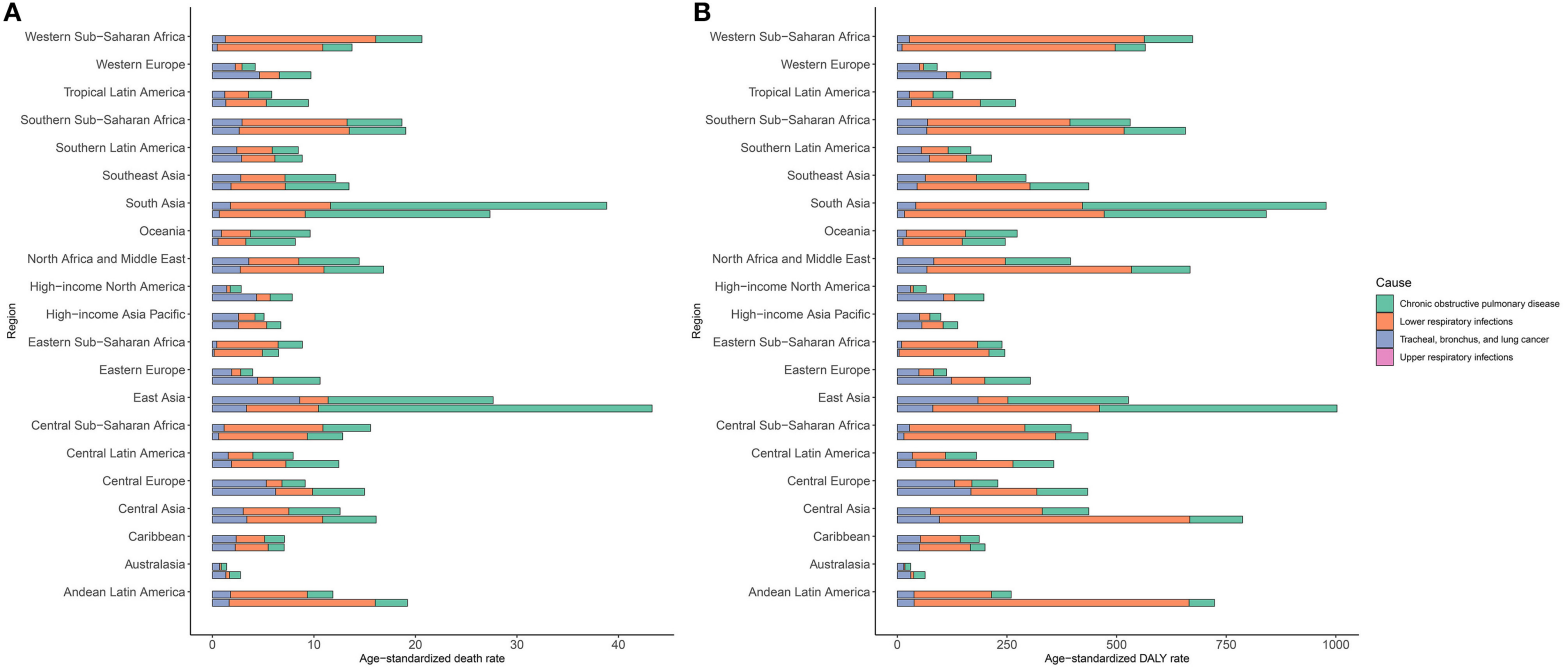
Burden of respiratory diseases attributable to ambient particulate matter pollution among 21 regions in 1990 and 2019. The upper column in each group is data for 2019 and the lower column is for 1990. **(A)**. age-standardized death rate of respiratory diseases; **(B)**. age-standardized DALY rate of respiratory diseases. DALY, disability-adjusted life year.

Regionally, over the past 30 years, South Asia had the highest increase in exposure to APMP, and then showed the highest APMP-related age-standardized death and DALY rates of COPD and LRIs worldwide (Figure 3, Supplementary Tables 1, 3, 4). East Asia had the second-highest increase in exposure to APMP; in addition, East Asia recorded the heaviest age-standardized death and DALY rates of TBL cancer attributable to APMP in 2019, followed by Central Europe (Figure 3, Supplementary Tables 1, 3, 4). For URIs, Central Asia has always had the highest burden over the past three decades (Figure 3, Supplementary Tables 1, 3, 4). In contrast, Australasia showed the lowest age-standardized death and DALY rates of COPD and LRIs attributable to APMP (Figure 3, Supplementary Tables 3, 4).

**Figure 4:**
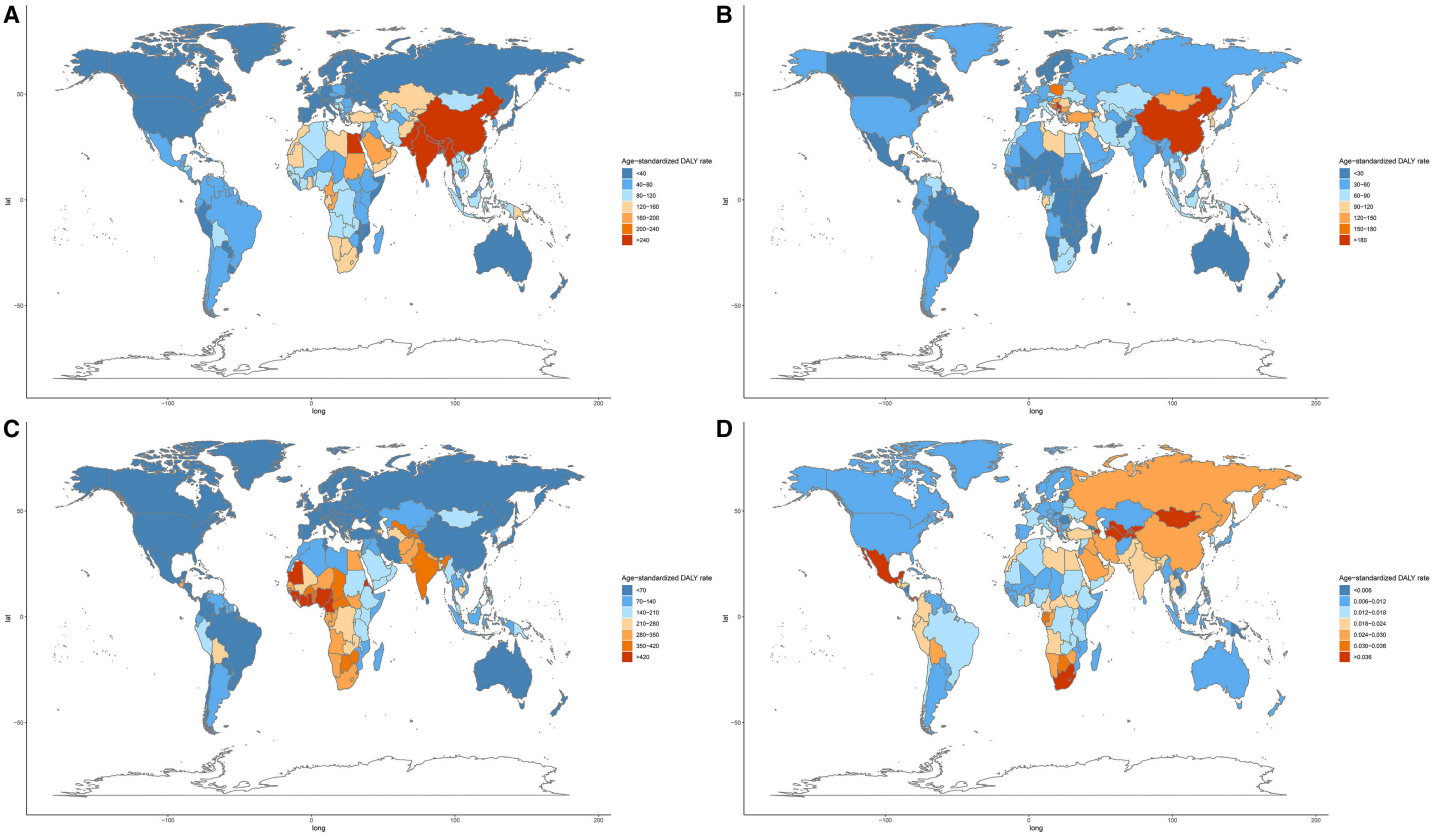
Age-standardized DALY rate of respiratory diseases attributable to ambient particulate matter pollution among 204 countries and territories in 2019. **(A)**. COPD; **(B)**. TBL cancer; **(C)**. LRIs; **(D)**. URIs. COPD, chronic obstructive pulmonary disease; DALY, disability-adjusted life year; TBL, cancer tracheal, bronchus, and lung cancer; LRIs, lower respiratory infections; URIs, upper respiratory infections.

At the national level, in 2019, the highest age-standardized DALY and death rates of COPD attributable to APMP were in Nepal, followed by India and Pakistan (Figure 4A, Supplementary Figure 2A), whereas the highest increase in APMP-related age-standardized DALY and death rates of COPD from 1990 were in Nicaragua (Supplementary Table 5). For LRIs, the age-standardized death and DALY rates attributable to APMP decreased obviously in most countries from 1990, but increased in 69 countries, among which Cameroon showed the highest age-standardized DALY and death rates (Figure 4B, Supplementary Figure 2B, Supplementary Table 5). In 2019, the ASDR of TBL cancer attributable to long-term exposure to APMP was the highest in China (Supplementary Figure 2C, Supplementary Table 5), whereas the APMP-related age-standardized DALY rate of TBL cancer was highest in Serbia (Figure 4C, Supplementary Table 5). Meanwhile, Equatorial Guinea showed the highest increase in the age-standardized death and DALY rates of TBL cancer attributable to APMP during the past 30 years (Supplementary Table 5). For URIs, age-standardized DALY and death rates attributable to APMP were at a low level, with rates of < 1 per 100,000 persons in all countries and territories; however, Uzbekistan, Azerbaijan, and Tajikistan had the highest APMP-related age-standardized death and DALY rates of URIs in 2019 (Figure 4D, Supplementary Figure 2D, Supplementary Table 5). The national relative burdens of different respiratory diseases attributable to APMP are described in Supplementary Table 6.

### Association Between SDI and Respiratory Diseases Attributable to APMP

**Figure 5:**
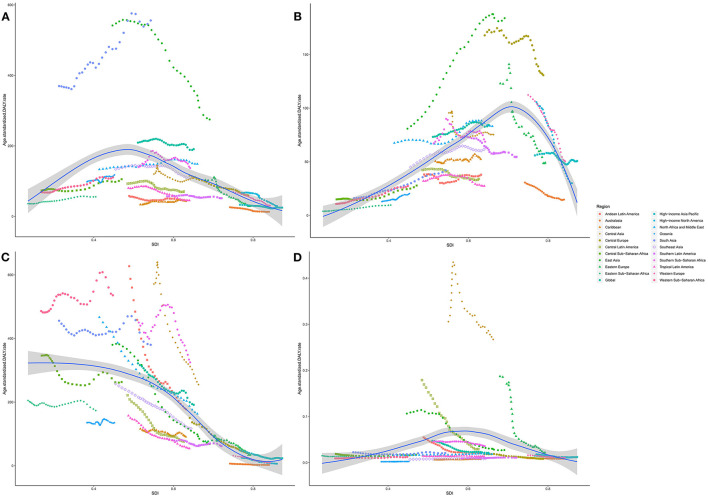
The association between SDI and age-standardized DALY rate of respiratory diseases attributable to ambient particulate matter pollution among regions. **(A)**. COPD; **(B)**. TBL cancer; **(C)**. LRIs; **(D)**. URIs. SDI, socio-demographic index; COPD, chronic obstructive pulmonary disease; DALY, disability-adjusted life year; TBL, cancer tracheal, bronchus, and lung cancer; LRIs, lower respiratory infections; URIs, upper respiratory infections.

Age-standardized DALY and death rates of respiratory diseases attributable to APMP also varied substantially with SDI (Figure 5, Supplementary Figure 3). The APMP-related age-standardized DALY and death rates of COPD showed the highest levels when SDI was approximately 0.5, above which the rates decreased with SDI. Similarly, for TBL cancer, the age-standardized DALY and death rates attributable to APMP increased dramatically with SDI, peaking when SDI was 0.66, and then decreased accordingly. However, for LRIs, the age-standardized DALY and death rates attributable to APMP decreased steadily with SDI, whereas for URIs, the APMP-related age-standardized death and DALY rates increased with SDI, showing higher levels in countries in the middle SDI quintile, and then declined in the high SDI quintile.

Over the past 30 years, the APMP-attributable burden of respiratory diseases also differed in five SDI quintiles (Supplementary Figure 4). From 1990, age-standardized death and DALY rates of COPD attributable to APMP exposure decreased significantly in high-middle and high-SDI quintiles, whereas they increased significantly in low- and low-middle SDI quintiles (Supplementary Figure 4). For LRIs, countries with low and low-middle SDI always showed higher age-standardized death and DALY rates attributable to APMP, although they underwent a great fluctuation and saw obvious declines in the last 5 years. In contrast, the age-standardized death and DALY rates of LRIs attributable to APMP decreased dramatically in middle and high-middle SDI quintiles. In terms of TBL cancer, increasing age-standardized death and DALY rates attributable to APMP were observed in low-to-middle SDI quintiles, whereas the highest rates were always found in high-middle SDI quintile. For high SDI quintile, APMP-associated age-standardized death and DALY rates of TBL cancer declined from 1990 to 2019, and showed lower rates compared with countries with middle SDI in 2019. However, the age-standardized death and DALY rates of URIs attributable to APMP decreased regardless of SDI (Supplementary Figure 4).

## Discussion

In this study, we presented a comprehensive assessment of the spatiotemporal patterns of respiratory disease burden attributed to APMP across 204 countries and territories. Our estimations showed that the total deaths and DALYs of respiratory diseases attributable to APMP increased in the past three decades, but the corresponding age-standardized death and DALY rates had different trends. There was a decrease in the age-standardized death and DALY rates of COPD, LRIs, and URIs attributable to APMP, while there was an increasing trend in the age-standardized rates of TBL cancer. Thus, APMP exposure remains a significant threat to public health worldwide.

Lung cancer is the most common cause of cancer deaths in both sexes in 2020 (17). However, compared with TBL cancer (lung cancer), the total numbers of deaths and DALYs of COPD and LRIs were much higher (18). Multiple risk factors contribute to the substantial respiratory disease burden to varying extents. As smoking is another risk factor for TBL cancer, tobacco smoking-associated TBL cancer burden has decreased over the past 30 years, especially among men in the United States, United Kingdom, and Australia, mainly due to the tobacco control policies in these countries (19, 20). However, in recent years, substantial cases have been diagnosed among non-smokers, for whom ambient air pollution may play a vital role as the second risk for TBL cancer (4, 21).

Our results indicated that the APMP-related deaths and DALYs of LRIs were obviously higher among children aged < 5 years, predominantly among infants, whereas APMP contributed substantially to the deaths and DALYs among older people with COPD and TBL cancer. Among children, exposure to PM_2.5_ may contribute partially to the susceptibility to respiratory syncytial virus and influenza (22, 23), potentially interpreting the higher deaths and DALYs of LRIs attributable to APMP in our analysis. For the elderly population, the high APMP-related burden of respiratory diseases may be explained by the higher susceptibility to ambient PM_2.5_ among older people and the accumulating effects of exposure to outdoor PM_2.5_ with age (24, 25). The potential mechanism of how PM_2.5_ increases the risk of deaths among older people is poorly studied, but it may involve systematic inflammation that may aggravate the severity of respiratory diseases (26).

We also found that the burden of respiratory diseases from exposure to APMP varied substantially with SDI and across regions. The age-standardized APMP-related burdens of LRIs and COPD were highest in countries with low-middle SDI, especially in countries in South Asia, North Africa, and the Middle East. However, the relative burdens of LRIs and COPD attributable to APMP were the highest in Qatar and Egypt. For TBL cancer, countries with high-middle SDI showed the highest burden, which may be due to their improved screening system (27). Nonetheless, across different regions, East Asia had the highest age-standardized burden of TBL cancer, especially China. A study has demonstrated that each 10 μg/m^3^ increment of PM_2.5_ exposure in China is linked to a 12% increase in lung cancer mortality among Chinese men (28). There are several reasons for the pronounced disease burden in these regions. First, the burning of coal, disposal of agricultural residue, and increasing number of vehicles and industrial factories in countries with lower SDI have led to an increase in ambient PM_2.5_ pollution (29–31). Second, compared with developed countries, the high population and limited medical support in lower SDI countries can help to interpret the higher mortality of respiratory diseases (32, 33). However, genetic differences among different populations and the changing patterns of population structure and lifestyles may also be the causes for the imbalanced burden in different countries (34–36), which were not assessed in this study. Therefore, in the future, more studies are needed to investigate the underlying causes and mechanisms of ambient PM_2.5_ leading to different diseases.

Great efforts have been made in recent years to reduce air pollution and the corresponding disease burden. In 2013, the World Health Organization designated PM_2.5_ as a carcinogen, and recommended that population-weighted ambient PM_2.5_ pollution should be lower than 10 μg/m^3^. Nonetheless, the concentration of PM_2.5_ remains pronounced in some areas, especially in China and India (37). China has implemented systematic regulations since 2013, and annual average concentrations of PM_2.5_ dropped by 33.3% from 2013 to 2017 (38). However, 81% of the Chinese population remains exposed to a mean PM_2.5_ concentration of >35 μg/m^3^, the recommended PM_2.5_ concentration by Chinese air quality management (39). Certainly, ambient PM_2.5_ pollution continues to be a salient issue for all countries, particularly in developing countries. In higher-income countries, variable strategies have been implemented to reduce air pollution, including switching to cleaner fuels, improving technologies in emission control, and strengthening public education on the environment (40, 41). More sustainable and eco-friendly strategies are needed to reduce air pollution levels and attributable disease burden, as well as meet climate goals simultaneously.

The main strength of the present study is that it provides the first comprehensive and recent review on respiratory disease burden attributable to APMP globally using the latest estimated data of the GBD 2019. However, the limitations of our study should be acknowledged. First, the burden of respiratory diseases attributable to APMP might be underestimated because of the insufficient evidence and research in lower-income countries and territories where the health registration systems might only cover a small number of people. Second, the joint effects of PM_2.5_ and other risk factors, such as ambient temperature, household air pollution, and high blood pressure, could generate more complex interactions, which were not evaluated in the present study (42, 43). Third, ASRs were used to enhance the comparability of disease burden among different populations; however, this might be affected by the population size and the choice of the standard population (44). Therefore, our results should be interpreted with caution. Finally, we analyzed only the PM_2.5_-related burden of respiratory diseases. Future research on this topic needs to be undertaken to shed more light on the association between APMP and different diseases.

## Conclusion

This study identified the substantial and increasing contribution of APMP to the global burden of respiratory diseases in the past 30 years. Countries and territories with low and low-middle SDI showed the highest but decreasing burden of LRIs and COPD in most older people and children aged < 5 years, respectively; these two age groups also had the highest mortality. People in countries with high-middle SDI, especially older people, also bore a huge and rising burden of TBL cancer. Consequently, strengthened efforts and management programs for reducing pollution levels are needed to avoid increases in burden, especially in people aged > 50 years and children aged < 5 years, as well as in countries with lower SDI. Our study provides insights that can assist in the development of targeted programs and policies for future air quality management in different regions.

## Data Availability Statement

All data in this analysis can be available through GBD query tool (http://ghdx.healthdata.org/gbd-results-tool), and the original contributions presented in the study are included in the article/Supplementary Material, further inquiries can be directed to the corresponding authors.

## Author Contributions

JG: conceptualization, supervision, formal analysis, visualization, and writing—review and editing. ZD: conceptualization, data curation, and formal analysis. YW: writing—original draft, methodology, and visualization. PS: writing—original draft and methodology. SL: writing—review and editing and software. LP: methodology, visulisation, and writing—review and editing. YL, YD, XD, YZe, and JH: methodology and writing—review and editing. WL, SY, and DX: writing—review and editing and visualization. YZu, MW, ZZ, and DZ: writing—review and editing. All authors contributed to the article and approved the submitted version.

## Conflict of Interest

The authors declare that the research was conducted in the absence of any commercial or financial relationships that could be construed as a potential conflict of interest.

## Publisher's Note

All claims expressed in this article are solely those of the authors and do not necessarily represent those of their affiliated organizations, or those of the publisher, the editors and the reviewers. Any product that may be evaluated in this article, or claim that may be made by its manufacturer, is not guaranteed or endorsed by the publisher.

## Abbreviations

APMP: ambient particulate matter pollution
ASDR: age-standardized death rate
COPD: chronic obstructive pulmonary disease
LRI: lower respiratory infection
URI: upper respiratory infection
TBL cancer, tracheal, bronchus: and lung cancer
SDI: socio-demographic index
GBD: global burden of disease
DALY: disability-adjusted life year.
