# Supplementary material for: Global Burden of Respiratory Diseases Attributable to Ambient Particulate Matter Pollution: Findings From the Global Burden of Disease Study 2019
Source: Front Public Health. 2021 Nov 23;9:740800. doi: 10.3389/fpubh.2021.740800 (PMC8650086; doi:10.3389/fpubh.2021.740800)
Supplement: Supplementary file 1 [file Data_Sheet_1.docx]

**Supplementary materials:**

**
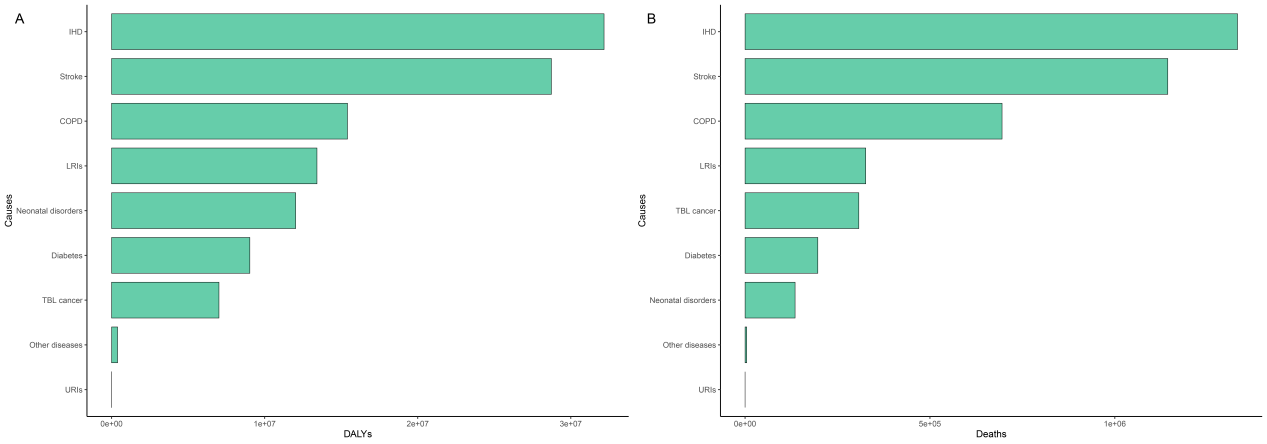
**

**Figure S1 Global burden of all causes attributable to ambient particulate matter pollution.** A. DALY; B. Deaths.

Abbreviation: DALY disability-adjusted life-year.


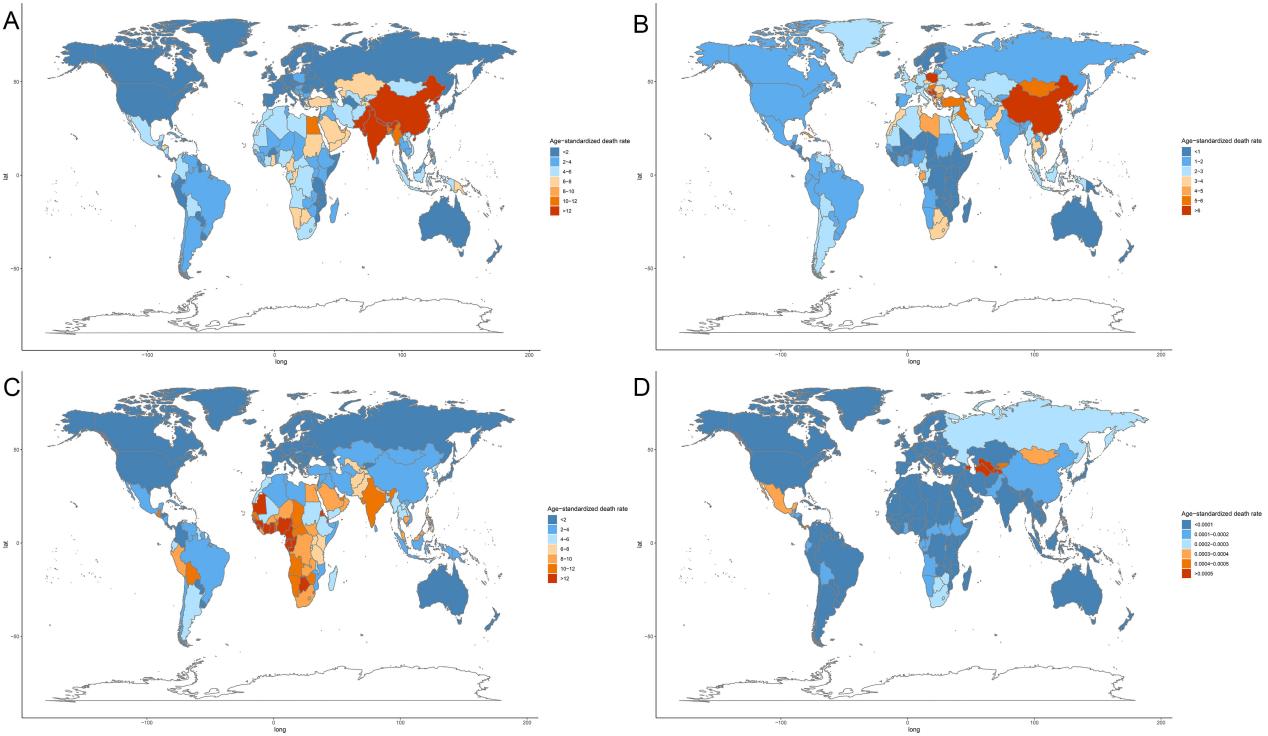


**Figure S2 Age-standardized death rate of respiratory diseases attributable to ambient particulate matter pollution among 204 countries and territories in 2019.** A. COPD; B. TBL cancer; C. LRIs; D. URIs.

Abbreviations: COPD chronic obstructive pulmonary disease; LRIs lower respiratory infections; TBL cancer tracheal, bronchus, and lung cancer; URIs upper respiratory infections.


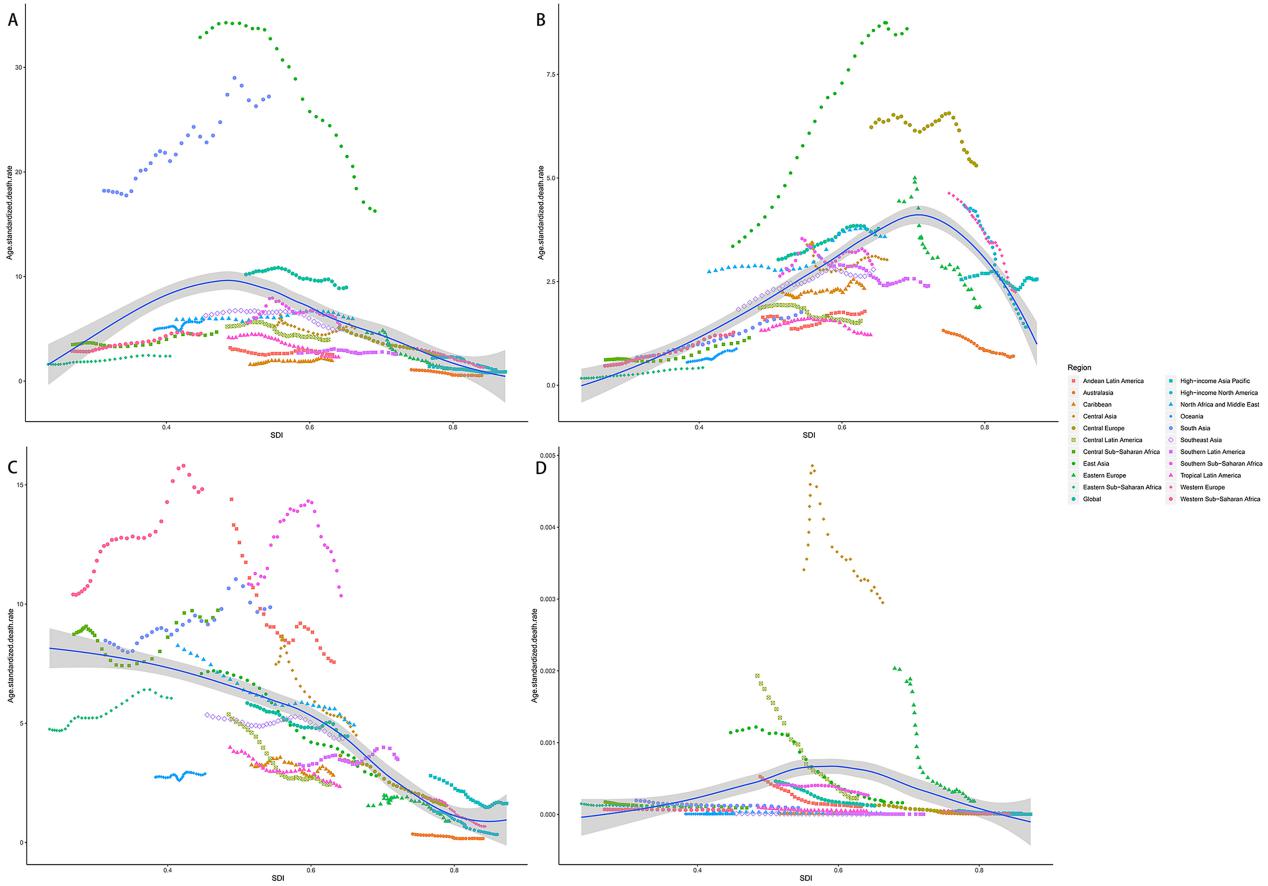


**Figure S3 Association between SDI and age-standardized death rate of respiratory diseases among regions.** A. COPD; B. TBL cancer; C. LRIs; d. URIs.

Abbreviations: COPD chronic obstructive pulmonary disease; LRIs lower respiratory infections; TBL cancer tracheal, bronchus, and lung cancer; SDI socio-demographic index; URIs upper respiratory infections.


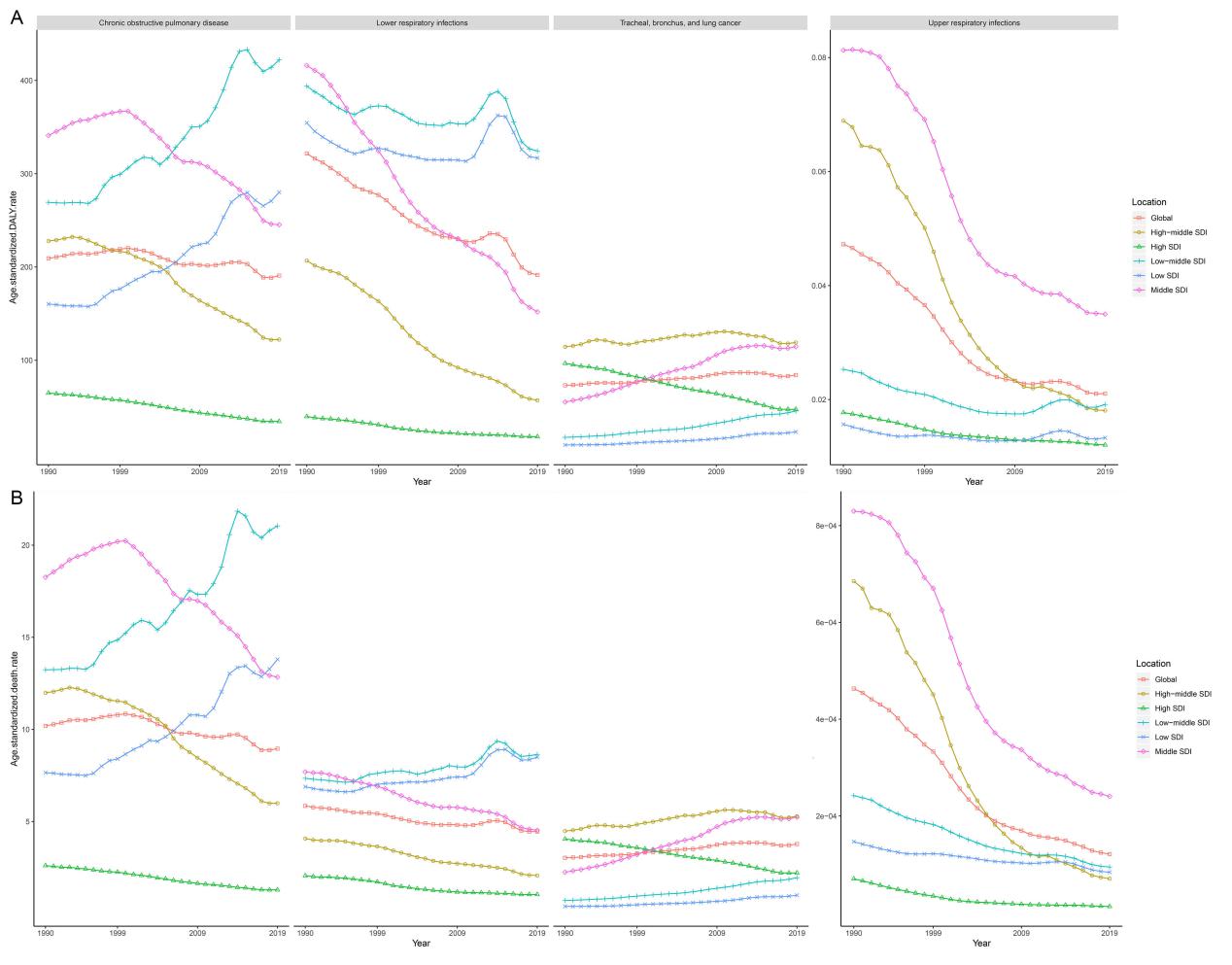


**Figure S4 Temporal trends in age-standardized burden of respiratory diseases attributable to ambient fine particulate matter pollution among five SDI regions from 1990 to 2019.** A. age-standardized DALY rate; B. age-standardized death rate.

Abbreviations: SDI socio-demographic index; DALY disability-adjusted life-year.

**Table S1 Global and regional age-standardized SEV of ambient fine particulate matter pollution from 1990 to 2019.**

| **Regions** | **Age-standardized SEV (95% UI)** | | | |
| --- | --- | --- | --- | --- |
|  | 1990 | 2010 | 2019 | Percentage change from 1990 to 2019 (%) |
| Global | 15.65(10.62 to 21.58) | 22.98(18.28 to 27.62) | 26.22(21.57 to 30.50) | 67.47(31.54 to 119.53) |
| High SDI | 15.43(9.51 to 22.60) | 13.88(10.92 to 17.43) | 12.98(10.03 to 16.40) | -15.88(-40.00 to 23.93) |
| High-middle SDI | 20.03(14.19 to 26.98) | 27.89(23.85 to 32.44) | 27.72(24.15 to 31.99) | 38.44(6.69 to 86.38) |
| Middle SDI | 18.42(11.63 to 26.25) | 30.57(24.83 to 35.59) | 33.50(28.84 to 38.24) | 81.92(31.00 to 168.94) |
| Low-middle SDI | 9.64(4.14 to 18.36) | 18.94(11.81 to 26.43) | 27.34(19.59 to 34.22) | 183.75(66.41 to 476.94) |
| Low SDI | 6.69(2.21 to 14.92) | 11.51(5.69 to 19.86) | 16.74(10.00 to 24.72) | 150.39(51.61 to 433.64) |
| Andean Latin America | 18.23(8.34 to 34.00) | 21.00(15.04 to 27.88) | 23.14(16.68 to 30.56) | 26.94(-27.41 to 162.94) |
| Australasia | 4.90(0.54 to 12.23) | 3.68(1.06 to 6.80) | 3.57(0.89 to 6.77) | -27.04(-79.52 to 302.44) |
| Caribbean | 11.29(4.27 to 22.42) | 12.22(6.80 to 19.92) | 13.04(6.84 to 21.89) | 15.48(-40.62 to 166.81) |
| Central Asia | 17.78(8.19 to 34.82) | 22.35(14.71 to 31.03) | 25.42(16.68 to 36.41) | 42.98(-22.78 to 175.77) |
| Central Europe | 23.30(11.38 to 38.00) | 22.34(18.91 to 26.48) | 19.05(15.97 to 22.74) | -18.27(-49.70 to 59.35) |
| Central Latin America | 18.50(8.98 to 32.98) | 18.53(14.49 to 22.92) | 18.26(14.22 to 22.80) | -1.30(-42.83 to 101.19) |
| Central Sub-Saharan Africa | 5.67(1.68 to 13.59) | 7.33(3.00 to 14.51) | 12.26(6.06 to 20.72) | 115.99(41.11 to 313.23) |
| East Asia | 16.87(7.58 to 30.55) | 34.41(27.45 to 40.63) | 37.05(31.55 to 42.63) | 119.60(20.92 to 367.62) |
| Eastern Europe | 18.21(7.43 to 32.64) | 15.22(10.35 to 20.54) | 10.78(6.54 to 15.41) | -40.80(-67.55 to 20.27) |
| Eastern Sub-Saharan Africa | 3.27(1.19 to 7.18) | 6.19(2.87 to 11.12) | 7.89(4.18 to 12.75) | 141.31(58.66 to 301.93) |
| High-income Asia Pacific | 15.07(5.60 to 27.86) | 13.64(10.27 to 17.60) | 17.03(12.50 to 22.27) | 13.01(-39.99 to 184.96) |
| High-income North America | 11.54(3.99 to 23.32) | 6.79(4.33 to 9.62) | 4.87(2.56 to 7.55) | -57.74(-81.28 to 11.15) |
| North Africa and Middle East | 26.68(20.91 to 32.63) | 37.92(32.28 to 44.22) | 37.38(31.91 to 43.63) | 40.10(25.23 to 63.70) |
| Oceania | 3.64(0.98 to 9.76) | 4.78(1.48 to 12.13) | 5.39(1.61 to 13.54) | 48.07(-24.13 to 212.46) |
| South Asia | 14.07(5.71 to 26.99) | 26.99(18.59 to 36.02) | 38.56(29.62 to 46.35) | 173.98(55.89 to 511.31) |
| Southeast Asia | 12.39(5.47 to 22.14) | 16.92(12.67 to 21.33) | 16.23(12.49 to 20.12) | 30.98(-26.95 to 175.63) |
| Southern Latin America | 13.87(5.42 to 25.56) | 14.06(10.11 to 18.26) | 14.52(9.96 to 19.68) | 4.68(-45.11 to 141.53) |
| Southern Sub-Saharan Africa | 16.15(11.77 to 21.26) | 21.53(17.08 to 26.80) | 22.35(17.62 to 28.04) | 38.37(19.04 to 66.91) |
| Tropical Latin America | 9.41(4.28 to 17.96) | 11.75(9.30 to 14.55) | 9.78(6.84 to 13.06) | 3.91(-48.74 to 128.93) |
| Western Europe | 18.01(7.30 to 31.86) | 13.82(10.78 to 17.41) | 9.85(7.11 to 13.06) | -45.30(-71.00 to24.81) |
| Western Sub-Saharan Africa | 9.09(3.81 to 18.47) | 14.77(8.27 to 24.19) | 21.53(13.71 to 32.08) | 136.98(57.07 to 288.13) |

Abbreviations: SEV summary exposure value; SDI socio-demographic index.

**Table S2 Burden of respiratory diseases attributable to ambient particulate matter pollution in different age groups in 2019.**

| **Age group** | **COPD** | | **LRIs** | | **TBL cancer** | | **URIs** | |
| --- | --- | --- | --- | --- | --- | --- | --- | --- |
|  | Deaths (95% UI) | DALYs (95% UI) | Deaths (95% UI) | DALYs (95% UI) | Deaths (95% UI) | DALYs (95% UI) | Deaths (95% UI) | DALYs (95% UI) |
| <1 year | – | – | 72731.06(48222 to 102809.64) | 6446844.03(4275552.74 to 9109848.06) | – | – | 7.85(4.37 to 11.94) | 1363.01(883.89 to 1961.72) |
| 01 to 04 | – | – | 21043.6(12105.68 to 32297.67) | 1817345.95(1046986.65 to 2785568.75) | – | – | – | – |
| 05 to 09 | – | – | 4087.54(2729.22 to 5730.53) | 341389.45(230700.06 to 474035.85) | – | – | – | – |
| 10 to 14 | – | – | 2198.38(1512.23 to 3015.13) | 174440.91(121959.36 to 237170.35) | – | – | – | – |
| 15 to 19 | – | – | 1873.53(1295.6 to 2615.08) | 139402.13(97188.34 to 192576.04) | – | – | – | – |
| 20 to 24 | – | – | 2141.73(1475.28 to 2934.08) | 147608.76(102742.98 to 201837.52) | – | – | – | – |
| 25 to 29 | 605.66(464.64 to 781.81) | 131221.47(102036.15 to 162013.51) | 2512.65(1757.99 to 3437.99) | 160049.99(112562.56 to 219225.13) | 487.98(355.81 to 620.45) | 30261.16(22104.76 to 38478.39) | – | – |
| 30 to 34 | 1060.8(805.15 to 1361.71) | 175826.47(137443.59 to 219865.95) | 2915.67(2043.77 to 3982.07) | 170444.29(120548.82 to 232417.49) | 1210.39(896.73 to 1546.79) | 68948.87(51062.93 to 88022.27) | – | – |
| 35 to 39 | 1703.38(1288.98 to 2205.4) | 211005.04(167846.01 to 262052.51) | 3581.81(2523.37 to 4840.61) | 190038.64(133644.6 to 255431.56) | 2218.8(1652.5 to 2828.51) | 115354.26(85883.35 to 147118.02) | – | – |
| 40 to 44 | 3160.65(2410.83 to 4065.46) | 292181.35(233182.9 to 359038.18) | 4239.21(3003.04 to 5733.99) | 203378.67(143812.27 to 276016.06) | 4528.19(3331.02 to 5741.1) | 213177.66(156775.32 to 270330.18) | – | – |
| 45 to 49 | 6478.62(4949.97 to 8233.35) | 497182.84(392210.44 to 615407.82) | 5314.89(3720.2 to 7196.17) | 228591.94(159953.27 to 310440.53) | 9530.39(6989.8 to 12287.46) | 402388.9(295730.8 to 520318.86) | – | – |
| 50 to 54 | 14494.37(11005.18 to 18312.48) | 852427.15(674243.78 to 1056298.36) | 7591.81(5304.86 to 10147.31) | 288220.38(201203.96 to 385536.95) | 18884.97(13687.49 to 24617.11) | 707237.52(513565.01 to 919654.87) | – | – |
| 55 to 59 | 24840.25(18909.63 to 31475.3) | 1161967.48(916442.76 to 1426692.11) | 10131.63(7168.76 to 13700.74) | 334880.64(237161.27 to 452062.37) | 28105.66(20550.82 to 36383.61) | 920511.67(675097.79 to 1188836.29) | – | – |
| 60 to 64 | 46230.11(36175.83 to 58196.02) | 1688267.7(1347287.75 to 2084406.23) | 15443.89(10793.42 to 20459.78) | 436706.48(305610.17 to 577214.33) | 39807.39(29300.05 to 51024.07) | 1119950.49(824365.33 to 1435651.96) | – | – |
| 65 to 69 | 73786.08(57942.72 to 92027.14) | 2207536.02(1772998.43 to 2709146.4) | 20749.42(14879.82 to 27643.56) | 492321.76(352233.85 to 655869.57) | 50355.35(36548.43 to 64670.45) | 1189047.36(863772.95 to 1527377.86) | – | – |
| 70 to 74 | 106258.52(83954.37 to 131545.35) | 2502418.92(2010602.08 to 3087509.33) | 26737.47(19188.04 to 35512.59) | 517531(371638.1 to 687927.8) | 51559.09(37884.23 to 66236.58) | 996728(732126.51 to 1283659.48) | – | – |
| 75 to 79 | 121239.89(95987.66 to 150338.66) | 2232090.85(1784164.63 to 2766460.63) | 30539.85(21824.39 to 40957.16) | 466334.57(332789.03 to 624956.21) | 42545.95(31360.61 to 54605.5) | 651076.6(479905.6 to 834504.8) | – | – |
| 80 plus | 295212.82(231479.22 to 365776.6) | 3461620.84(2755563.65 to 4321684.63) | 92519.06(63147.97 to 127991.36) | 862491.63(593326.72 to 1183381.43) | 58446.7(42804.47 to 74956.75) | 601121.18(441637.26 to 770060.25) | – | – |

Abbreviations: DALY disability-adjusted life year; COPD chronic obstructive pulmonary disease; TBL cancer tracheal, bronchus, and lung cancer; LRIs lower respiratory infections; URIs upper respiratory infections; UI uncertainty interval.

**Table S3 Regional burden of respiratory diseases attributable to ambient fine particulate matter pollution in 1990 and 2019.**

| **Regions** | **COPD** | | | | **TBL cancer** | | | | **LRIs** | | | | **URIs** | | | |
| --- | --- | --- | --- | --- | --- | --- | --- | --- | --- | --- | --- | --- | --- | --- | --- | --- |
|  | Deaths, in thousands (95% UI) | | DALYs, in thousands (95% UI) | | Deaths, in thousands (95% UI) | | DALYs, in thousands (95% UI) | | Deaths, in thousands (95% UI) | | DALYs, in thousands (95% UI) | | Deaths, in thousands (95% UI) | | DALYs, in thousands (95% UI) | |
|  | 1990 | 2019 | 1990 | 2019 | 1990 | 2019 | 1990 | 2019 | 1990 | 2019 | 1990 | 2019 | 1990 | 2019 | 1990 | 2019 |
| Global | 351.73(220.23 to 514.84) | 695.07(553.83 to 861.86) | 7982.47(5057.85 to 11666.25) | 15413.75(12394.37 to 18965.15) | 118.44(79.18 to 163.89) | 307.68(226.68 to 395.67) | 3011.08(2013.12 to 4154.52) | 7015.80(5176.47 to 9024.57) | 290.36(173.20 to 463.10) | 326.35(229.57 to 436.45) | 19463(11009.97 to 32484.82) | 13418.02(9211.57 to 18297.83) | 30.32(11.30 to 60.88) | 7.85(4.37 to 11.94) | 3.09(1.31 to 6.01) | 1363.01(883.89 to 1961.72) |
| High SDI | 27.93(14.99 to 43.36) | 28.01(17.38 to 41.37) | 683.48(378.38 to 1049.81) | 647.61(403.15 to 948.12) | 42.38(24.39 to 64.06) | 42.86(28.13 to 61.53) | 981.83(570.91 to 1478.22) | 840.34(549.59 to 1202.27) | 20.60(9.98 to 35.01) | 22.92(13.15 to 36.94) | 361.60(189.54 to 592.95) | 308.74(182.00 to 486.94) | 0.40(0.26 to 0.56) | 0.07(0.04 to 0.13) | 0.10(0.07 to 0.15) | 0.06(0.03 to 0.10) |
| High-middle SDI | 110.10(69.00 to 161.64) | 118.09(88.91 to 156.74) | 2316.25(1479.94 to 3313.36) | 2460.40(1889.86 to 3123.35) | 48.48(32.58 to 66.34) | 108.54(80.17 to 139.68) | 1277.05(858.09 to 1745.62) | 2448.76(1813.61 to 3157.18) | 38.53(25.54 to 56.29) | 36.46(23.78 to 52.43) | 2115.87(1393.63 to 3114.13) | 805.00(530.58 to 1135.06) | 6.82(3.27 to 11.53) | 0.53(0.31 to 1.48) | 0.69(0.36 to 1.14) | 0.14(0.08 to 0.23) |
| Middle SDI | 139.68(81.02 to 216.66) | 257.35(204.04 to 322.92) | 3152.38(1872.32 to 4830.73) | 5599.12(4493.53 to 6988.68) | 22.52(12.50 to 34.57) | 125.48(90.30 to 162.73) | 615.66(337.84 to 954.22) | 2946.08(2113.90 to 3821.86) | 110.32(71.60 to 161.88) | 88.08(61.51 to 120.26) | 7891.03(5101.77 to 11663.40) | 3033.71(2113.52 to 4156.66) | 17.12(6.47 to 31.77) | 4.14(6.86 to 2.03) | 1.68(0.70 to 3.04) | 0.60(0.36 to 0.91) |
| Low-middle SDI | 60.52(26.33 to 103.86) | 238.33(173.36 to 304.96) | 1480.53(648.98 to 2560.47) | 5401.02(4080.37 to 6828.73) | 4.13(1.82 to 7.39) | 25.84(16.85 to 34.97) | 112.15(49.32 to 199.58) | 650.91(422.69 to 882.25) | 81.10(34.86 to 155.73) | 113.05(80.13 to 147.73) | 60.45(2545.93 to 11738.23) | 5148.12(3528.02 to 6871.46) | 4.35(0.29 to 13.65) | 1.60(0.49 to 3.04) | 0.45(0.07 to 1.32) | 0.32(0.17 to 0.53) |
| Low SDI | 13.45(4.88 to 26.32) | 53.18(37.33 to 71.55) | 348.45(127.16 to 676.17) | 1302.65(922.30 to 1719.74) | 0.87(0.27 to 1.91) | 4.86(2.86 to 7.31) | 23.15(7.09 to 50.80) | 127.43(74.68 to 191.45) | 39.70(13.27 to 87.91) | 65.72(37.12 to 103.27) | 3043.64(999.73 6908.07) | 4117.66(2234.64 to 6794.38) | 1.64(0.03 to 6.04) | 1.50(0.12 to 4.02) | 0.17(0.02 to 0.60) | 0.24(0.08 to 0.51) |
| Andean Latin America | 0.55(0.26 to 0.98) | 1.32(0.84 to 1.92) | 11.55(5.62 to 20.89) | 24.53(16.32 to 35.41) | 0.32(0.14 to 0.56) | 0.98(0.62 to 1.43) | 8.14(3.49 to 14.26) | 21.46(13.45 to 31.41) | 4.61(2.12 to 8.29) | 4.10(2.31 to 6.57) | 297.49(136.15 to 542.79) | 104.43(59.22 to 167.62) | 0.30(0.03 to 0.91) | 0.07(0.02 to 0.15) | 0.03(0.01 to 0.09) | 13.36(6.52 to 24.35) |
| Australasia | 0.25(0.03 to 0.67) | 0.30(0.07 to 0.61) | 6.19(0.66 to 16.01) | 6.67(1.61 to 13.68) | 0.31(0.03 to 0.80) | 0.36(0.09 to 0.69) | 7.16(0.75 to 18.13) | 7.01(1.67 to 13.71) | 0.07(0.01 to 0.20) | 0.10(0.02 to 0.21) | 1.32(0.20 to 3.59) | 1.13(0.30 to 2.47) | 0.00(0.00 to 0.01) | 0.00(0.00 to 0.00) | 0.00(0.00 to 0.00) | 1.46(0.17 to 3.95) |
| Caribbean | 0.38(0.16 to 0.70) | 1.01(0.53 to 1.70) | 8.63(3.55 to 16.25) | 22.31(11.69 to 37.51) | 0.57(0.23 to 1.04) | 1.21(0.62 to 2.00) | 13.32(5.29 to 24.28) | 27.25(13.88 to 45.33) | 0.92(0.36 to 1.86) | 1.39(0.68 to 2.50) | 42.72(16.03 to 89.74) | 40.61(19.59 to 76.08) | 0.01(0.00 to 0.02) | 0.00(0.00 to 0.01) | 0.00(0.00 to 0.01) | 3.05(1.39 to 5.70) |
| Central Asia | 2.20(1.10 to 3.96) | 2.78(1.78 to 4.07) | 55.82(28.16 to 101.75) | 72.36(45.95 to 104.67) | 1.65(0.82 to 2.71) | 2.23(1.53 to 3.05) | 48.49(23.96 to 79.63) | 61.71(42.03 to 85.45) | 6.37(2.82 to 12.44) | 3.62(2.00 to 6.01) | 522.13(231.84 to 1021.28) | 231.55(125.13 to 393.93) | 3.19(1.08 to 6.45) | 2.68(0.76 to 5.14) | 0.29(0.10 to 0.58) | 242.51(71.07 to 461.85) |
| Central Europe | 6.98(3.57 to 11.05) | 5.14(3.66 to 6.86) | 167.79(87.07 to 263.28) | 124.79(89.48 to 167.41) | 9.34(4.77 to 14.33) | 11.28(8.12 to 14.92) | 252.10(128.59 to 385.92) | 263.47(189.52 to 350.34) | 4.22(1.95 to 7.33) | 3.09(1.92 to 4.70) | 155.36(71.49 to 272.00) | 59.63(37.19 to 90.85) | 0.11(0.05 to 0.17) | 0.01(0.00 to 0.03) | 0.01(0.01 to 0.02) | 4.24(2.14 to 7.65) |
| Central Latin America | 3.45(1.73 to 5.66) | 8.73(5.89 to 12.05) | 72.08(36.67 to 118.25) | 161.17(110.24 to 220.40) | 1.48(0.75 to 2.38) | 3.58(2.45 to 4.87) | 36.61(18.47 to 58.94) | 81.02(55.47 to 111.67) | 6.72(3.20 to 12.06) | 5.48(3.40 to 8.32) | 423.22(201.23 to 754.65) | 173.96(108.58 to 268.51) | 4.58(2.41 to 7.12) | 0.48(0.32 to 0.70) | 0.42(0.22 to 0.66) | 61.38(41.42 to 86.94) |
| Central Sub-Saharan Africa | 0.54(0.18 to 1.17) | 1.74(0.88 to 3.23) | 15.47(5.25 to 33.47) | 52.73(28.27 to 93.86) | 0.13(0.03 to 0.37) | 0.58(0.27 to 1.18) | 3.73(0.91 to 10.32) | 16.41(7.43 to 33.67) | 3.96(1.18 to 9.36) | 5.86(2.93 to 10.40) | 281.83(81.58 to 675.55) | 292.03(140.90 to 520.88) | 0.22(0.00 to 0.84) | 0.20(0.02 to 0.60) | 0.02(0.00 to 0.08) | 36.47(12.10 to 82.54) |
| East Asia | 194.94(96.97 to 323.13) | 270.50(208.79 to 354.47) | 4028.19(2032.12 to 6717.24) | 5194.06(4075.74 to 6550.52) | 28.50(12.74 to 48.13) | 174.51(124.63 to 231.51) | 761.27(337.16 to 1289.14) | 3930.34(2782.27 to 5273.70) | 63.42(29.01 to 110.73) | 39.46(26.62 to 54.75) | 4366.30(1967.33 to 7766.68) | 843.55(573.56 to 1150.88) | 13.73(1.19 to 31.41) | 1.21(0.57 to 3.57) | 1.28(0.14 to 2.88) | 203.08(113.71 to 408.04) |
| Eastern Europe | 11.93(5.05 to 21.20) | 4.13(2.24 to 6.63) | 285.67(121.51 to 507.63) | 99.56(54.11 to 157.02) | 12.79(5.84 to 21.38) | 6.56(3.77 to 9.70) | 354.19(162.00 to 592.53) | 165.39(95.96 to 246.98) | 3.17(1.25 to 5.84) | 2.55(1.32 to 4.38) | 141.54(58.99 to 257.36) | 81.71(42.87 to 138.44) | 2.87(1.88 to 3.70) | 0.20(0.13 to 0.28) | 0.26(0.17 to 0.34) | 23.28(15.78 to 32.21) |
| Eastern Sub-Saharan Africa | 0.93(0.35 to 1.90) | 2.97(1.75 to 4.60) | 26.33(10.29 to 53.63) | 89.28(53.87 to 136.17) | 0.12(0.04 to 0.27) | 0.64(0.32 to 1.07) | 3.07(0.97 to 7.12) | 16.53(8.15 to 27.73) | 8.08(2.63 to 18.98) | 12.49(6.81 to 20.64) | 589.54(185.22 to 1403.75) | 670.16(346.92 to 1132.50) | 0.63(0.01 to 2.74) | 0.65(0.04 to 1.95) | 0.06(0.00 to 0.26) | 85.44(21.38 to 227.66) |
| High-income Asia Pacific | 2.44(0.88 to 4.59) | 5.33(3.23 to 8.02) | 63.79(22.49 to 122.34) | 118.39(75.96 to 172.36) | 5.05(1.82 to 9.13) | 12.57(8.28 to 17.95) | 114.79(42.14 to 204.91) | 213.45(143.41 to 301.49) | 4.56(1.33 to 9.39) | 10.05(5.44 to 16.40) | 79.89(24.68 to 159.71) | 112.13(61.48 to 179.16) | 0.04(0.02 to 0.09) | 0.00(0.00 to 0.00) | 0.01(0.01 to 0.02) | 8.06(3.77 to 15.30) |
| High-income North America | 8.10(2.51 to 16.44) | 7.40(3.44 to 12.56) | 236.59(75.14 to 468.40) | 186.86(86.24 to 312.32) | 15.18(5.64 to 29.83) | 9.06(4.33 to 15.16) | 351.17(130.96 to 688.00) | 183.82(88.27 to 309.61) | 4.85(1.42 to 10.58) | 2.32(1.02 to 4.50) | 83.26(25.06 to 178.23) | 34.55(15.62 to 65.93) | 0.13(0.09 to 0.17) | 0.03(0.02 to 0.05) | 0.04(0.02 to 0.06) | 22.21(12.70 to 39.00) |
| North Africa and Middle East | 7.83(5.82 to 10.20) | 20.71(15.16 to 26.89) | 222.35(170.04 to 282.85) | 631.04(472.87 to 809.01) | 4.60(3.14 to 6.26) | 14.83(10.90 to 19.16) | 125.52(85.29 to 171.92) | 382.38(280.19 to 495.61) | 29.72(19.77 to 43.35) | 20.24(13.78 to 28.64) | 2275.64(1506.34 to 3372.02) | 864.47(580.60 to 1230.88) | 0.24(0.01 to 0.64) | 0.09(0.01 to 0.18) | 0.09(0.04 to 0.15) | 108.34(61.47 to 183.17) |
| Oceania | 0.10(0.03 to 0.30) | 0.30(0.76 to 0.08) | 2.74(0.66 to 7.80) | 7.74(2.19 to 19.32) | 0.02(0.00 to 0.04) | 0.06(0.02 to 0.13) | 0.41(0.13 to 1.07) | 1.54(0.47 to 3.72) | 0.16(0.03 to 0.44) | 0.31(0.08 to 0.80) | 11.53(2.33 to 33.89) | 21.81(5.35 to 57.34) | 0.00(0.00 to 0.00) | 0.00(0.00 to 0.00) | 0.00(0.00 to 0.00) | 0.49(0.12 to 1.37) |
| South Asia | 72.57(31.14 to 128.58) | 307.63(223.92 to 392.82) | 1855.76(816.75 to 3298.51) | 7254.90(5550.88 to 9125.89) | 3.59(1.52 to 6.64) | 24.14(16.64 to 32.15) | 98.83(42.33 to 181.85) | 621.07(427.86 to 830.05) | 88.60(35.33 to 171.51) | 127.43(91.40 to 164.48) | 6668.71(2678.18 to 12848.76) | 5865.63(4113.53 to 7742.29) | 3.31(0.06 to 12.21) | 1.45(0.22 to 3.13) | 0.38(0.04 to 1.25) | 331.23(157.62 to 563.42) |
| Southeast Asia | 12.29(5.77 to 21.85) | 24.27(17.58 to 31.98) | 322.43(157.16 to 572.97) | 642.77(470.54 to 845.82) | 4.55(2.03 to 8.02) | 16.24(11.14 to 22.25) | 124.68(55.86 to 219.09) | 408.89(280.52 to 567.19) | 19.93(9.19 to 37.82) | 21.20(13.56 to 31.38) | 1348.85(596.03 to 2636.37) | 649.93(422.78 to 954.76) | 0.07(0.00 to 0.19) | 0.03(0.00 to 0.06) | 0.04(0.02 to 0.08) | 45.87(23.23 to 81.68) |
| Southern Latin America | 1.13(0.47 to 2.07) | 2.22(1.32 to 3.21) | 25.80(10.88 to 46.92) | 43.37(25.96 to 63.23) | 1.33(0.53 to 2.44) | 2.01(1.31 to 2.88) | 34.23(13.50 to 62.63) | 44.75(28.79 to 64.49) | 1.36(0.54 to 2.65) | 2.96(1.58 to 4.92) | 40.01(16.04 to 76.94) | 47.89(25.97 to 80.65) | 0.01(0.00 to 0.01) | 0.00(0.00 to 0.00) | 0.00(0.00 to 0.01) | 5.05(1.34 to 11.78) |
| Southern Sub-Saharan Africa | 1.28(0.91 to 1.76) | 2.50(1.75 to 3.37) | 38.17(27.84 to 50.62) | 75.37(53.30 to 100.85) | 0.72(0.46 to 1.08) | 1.59(1.12 to 2.19) | 19.84(12.86 to 29.65) | 40.74(28.54 to 56.27) | 4.26(2.90 to 6.05) | 5.72(3.62 to 8.40) | 249.79(165.33 to 358.55) | 227.82(144.44 to 333.86) | 0.31(0.11 to 0.61) | 0.21(0.09 to 0.43) | 0.03(0.01 to 0.06) | 28.37(15.01 to 49.97) |
| Tropical Latin America | 3.00(1.41 to 5.28) | 5.20(3.25 to 7.72) | 69.05(32.22 to 121.67) | 107.44(68.37 to 157.84) | 1.18(0.52 to 2.13) | 2.91(1.87 to 4.10) | 31.23(13.68 to 56.49) | 67.74(43.99 to 95.69) | 4.12(1.85 to 7.60) | 5.19(2.94 to 8.38) | 231.57(102.16 to 430.16) | 115.41(67.88 to 187.22) | 0.17(0.09 to 0.28) | 0.07(0.04 to 0.10) | 0.02(0.01 to 0.04) | 20.79(11.62 to 34.95) |
| Western Europe | 18.84(7.21 to 34.12) | 14.44(8.90 to 21.15) | 409.02(157.35 to 735.93) | 291.65(178.60 to 427.53) | 26.63(11.42 to 44.28) | 20.24(13.24 to 29.45) | 612.57(261.77 to 1016.16) | 408.08(266.62 to 591.20) | 10.94(3.62 to 21.41) | 7.77(4.22 to 12.93) | 160.70(53.80 to 316.13) | 88.52(49.57 to 148.31) | 0.12(0.05 to 0.23) | 0.02(0.01 to 0.04) | 0.04(0.02 to 0.06) | 21.85(10.99 to 39.68) |
| Western Sub-Saharan Africa | 1.99(0.92 to 3.61) | 6.46(4.27 to 9.32) | 59.06(27.92 to 108.12) | 206.78(137.79 to 296.84) | 0.39(0.15 to 0.77) | 2.13(1.21 to 3.16) | 9.72(3.68 to 19.37) | 52.75(29.19 to 79.40) | 20.31(7.69 to 44.00) | 45.04(25.54 to 73.18) | 1429.30(549.79 to 3274.97) | 2891.13(1513.24 to 4891.86) | 0.30(0.02 to 1.04) | 0.45(0.09 to 1.13) | 0.04(0.01 to 0.12) | 96.48(42.47 to 182.10) |

Abbreviations: DALY disability-adjusted life year; COPD chronic obstructive pulmonary disease; TBL cancer tracheal, bronchus, and lung cancer; LRIs lower respiratory infections; URIs upper respiratory infections; UI uncertainty interval.

**Table S4 Age-standardized death and DALY rates of respiratory diseases attributable to ambient particulate matter pollution among GBD regions in 1990 and 2019.**

| **Regions** | **COPD** | | | | **TBL cancer** | | | | **LRIs** | | | | **URIs** | | | |
| --- | --- | --- | --- | --- | --- | --- | --- | --- | --- | --- | --- | --- | --- | --- | --- | --- |
|  | ASDR, per 100,000 populations (95% UI) | | Age-standardized DALY rate, per 100,000 populations (95% UI) | | ASDR, per 100,000 populations (95% UI) | | Age-standardized DALY rate, per 100,000 populations (95% UI) | | ASDR, per 100,000 populations (95% UI) | | Age-standardized DALY rate, per 100,000 populations (95% UI) | | ASDR, per 100,000 populations (95% UI) | | Age-standardized DALY rate, per 100,000 populations (95% UI) | |
|  | 1990 | 2019 | 1990 | 2019 | 1990 | 2019 | 1990 | 2019 | 1990 | 2019 | 1990 | 2019 | 1990 | 2019 | 1990 | 2019 |
| Global | 10.19(6.42 to 14.89) | 8.95(7.14 to 11.10) | 209.29(133.30 to 305.04) | 190.79(153.52 to 234.76) | 3.03(2.03 to 4.18) | 3.78(2.79 to 4.86) | 73.16(48.94 to 101.24) | 84.22(62.13 to 108.30) | 5.85(3.60 to 9.05) | 4.46(3.13 to 5.95) | 321.70(184.41 to 529.72) | 191.42(130.56 to 262.85) | 0.00(0.00 to 0.00) | 0.00(0.00 to 0.00) | 0.05(0.02 to 0.09) | 0.02(0.01 to 0.03) |
| High SDI | 2.61(1.40 to 4.04) | 1.29(0.80 to 1.90) | 64.93(35.92 to 99.82) | 34.41(21.58 to 50.38) | 4.05(2.33 to 6.12) | 2.21(1.45 to 3.16) | 96.70(56.40 to 145.51) | 47.43(31.33 to 67.69) | 2.06(1.00 to 3.48) | 1.04(0.60 to 1.66) | 39.72(21.68 to 64.16) | 18.08(10.93 to 27.98) | 0.00(0.00 to 0.00) | 0.00(0.00 to 0.00) | 0.02(0.01 to 0.03) | 0.01(0.01 to 0.02) |
| High-middle SDI | 11.99(7.51 to 17.74) | 5.99(4.49 to 7.96) | 227.85(146.32 to 327.43) | 122.22(93.83 to 155.20) | 4.48(3.01 to 6.11) | 5.28(3.90 to 6.80) | 114.51(76.87 to 156.39) | 119.07(88.28 to 153.40) | 4.08(5.95 to 2.71) | 2.07(1.36 to 2.95) | 206.80(136.09 to 304.56) | 57.09(37.78 to 79.56) | 0.00(0.00 to 0.00) | 0.00(0.00 to 0.00) | 0.07(0.04 to 0.11) | 0.02(0.01 to 0.03) |
| Middle SDI | 18.26(10.67 to 28.32) | 12.84(10.17 to 16.27) | 340.93(201.42 to 521.77) | 245.28(196.83 to 305.61) | 2.25(1.26 to 3.44) | 5.24(3.79 to 6.77) | 55.23(30.56 to 84.96) | 114.61(82.42 to 148.60) | 7.69(5.06 to 11.19) | 4.53(3.15 to 6.15) | 415.96(269.15 to 610.37) | 151.91(105.85 to 207.90) | 0.00(0.00 to 0.00) | 0.00(0.00 to 0.00) | 0.08(0.03 to 0.15) | 0.03(0.02 to 0.05) |
| Low-middle SDI | 13.24(5.89 to 22.72) | 21.04(15.44 to 26.80) | 269.28(118.32 to 464.54) | 422.01(319.15 to 533.35) | 0.72(0.32 to 1.28) | 1.95(1.28 to 2.63) | 17.40(7.68 to 31.15) | 45.68(29.71 to 61.98) | 7.35(3.30 to 13.69) | 8.64(6.18 to 11.32) | 393.90(169.44 to 759.87) | 324.41(223.63 to 430.31) | 0.00(0.00 to 0.00) | 0.00(0.00 to 0.00) | 0.03(0.00 to 0.07) | 0.02(0.01 to 0.03) |
| Low SDI | 7.65(2.78 to 14.89) | 13.80(9.69 to 18.74) | 160.30(58.34 to 310.70) | 280.13(197.71 to 372.36) | 0.39(0.12 to 0.86) | 1.00(0.59 to 1.50) | 9.23(2.84 to 20.20) | 22.24(13.68 to 34.87) | 6.89(2.48 to 14.83) | 8.49(4.94 to 12.77) | 354.54(118.69 to 778.03) | 316.84(178.33 to 499.83) | 0.00(0.00 to 0.00) | 0.00(0.00 to 0.00) | 0.02(0.00 to 0.05) | 0.01(0.00 to 0.03) |
| Andean Latin America | 3.18(1.54 to 5.70) | 2.52(1.60 to 3.68) | 58.09(28.22 to 105.75) | 44.77(29.75 to 64.80) | 1.64(0.72 to 2.86) | 1.78(1.13 to 2.62) | 38.16(16.46 to 66.42) | 37.95(23.88 to 55.28) | 14.40(6.76 to 25.67) | 7.57(4.29 to 12.12) | 627.10(290.27 to 1137.11) | 176.91(100.30 to 283.85) | 0.00(0.00 to 0.00) | 0.00(0.00 to 0.00) | 0.05(0.01 to 0.15) | 0.02(0.01 to 0.04) |
| Australasia | 1.09(0.11 to 2.84) | 0.53(0.13 to 1.09) | 26.10(2.80 to 67.38) | 13.14(3.17 to 27.02) | 1.32(0.14 to 3.35) | 0.70(0.17 to 1.37) | 30.66(3.19 to 77.60) | 14.77(3.51 to 29.01) | 0.36(0.04 to 1.01) | 0.17(0.04 to 0.37) | 6.61(1.11 to 17.71) | 2.51(0.73 to 5.31) | 0.00(0.00 to 0.00) | 0.00(0.00 to 0.00) | 0.01(0.00 to 0.03) | 0.01(0.00 to 0.02) |
| Caribbean | 1.58(0.66 to 2.94) | 1.94(1.03 to 3.28) | 33.53(13.88 to 62.69) | 43.16(22.62 to 72.59) | 2.24(0.89 to 4.08) | 2.33(1.19 to 3.86) | 50.53(20.05 to 92.03) | 52.40(26.70 to 87.21) | 3.25(1.29 to 6.57) | 2.82(1.37 to 5.05) | 115.90(44.52 to 238.34) | 91.11(43.38 to 170.85) | 0.00(0.00 to 0.00) | 0.00(0.00 to 0.00) | 0.01(0.00 to 0.01) | 0.01(0.00 to 0.01) |
| Central Asia | 5.26(2.64 to 9.46) | 5.05(3.24 to 7.37) | 120.17(60.53 to 218.96) | 106.09(67.50 to 152.90) | 3.39(1.68 to 5.57) | 3.03(2.06 to 4.12) | 95.57(47.26 to 157.03) | 75.27(51.38 to 103.66) | 7.48(3.34 to 14.55) | 4.50(2.50 to 7.40) | 571.11(252.93 to 1115.87) | 255.06(137.97 to 432.21) | 0.00(0.00 to 0.01) | 0.00(0.00 to 0.01) | 0.31(0.10 to 0.62) | 0.27(0.08 to 0.51) |
| Central Europe | 5.11(2.62 to 8.08) | 2.28(1.62 to 3.05) | 115.84(60.06 to 181.99) | 59.16(42.39 to 79.20) | 6.23(3.18 to 9.57) | 5.30(3.82 to 7.03) | 167.82(85.59 to 257.06) | 130.66(93.70 to 174.04) | 3.64(1.68 to 6.35) | 1.55(0.97 to 2.36) | 150.38(69.53 to 261.36) | 39.37(24.60 to 59.49) | 0.00(0.00 to 0.00) | 0.00(0.00 to 0.00) | 0.02(0.01 to 0.03) | 0.01(0.00 to 0.01) |
| Central Latin America | 5.21(2.62 to 8.57) | 3.97(2.68 to 5.47) | 93.02(47.18 to 153.22) | 70.49(48.21 to 96.59) | 1.87(0.94 to 3.00) | 1.55(1.06 to 2.11) | 42.26(21.30 to 68.03) | 33.97(23.22 to 46.75) | 5.37(2.56 to 9.69) | 2.43(1.51 to 3.70) | 221.31(106.11 to 397.23) | 75.97(47.47 to 117.56) | 0.00(0.00 to 0.00) | 0.00(0.00 to 0.00) | 0.18(0.09 to 0.28) | 0.03(0.02 to 0.04) |
| Central Sub-Saharan Africa | 3.49(1.16 to 7.55) | 4.69(2.35 to 8.84) | 73.77(24.99 to 158.47) | 105.43(55.81 to 189.21) | 0.61(0.16 to 1.70) | 1.14(0.54 to 2.26) | 14.93(3.69 to 41.31) | 27.83(12.84 to 56.29) | 8.73(2.71 to 20.41) | 9.74(4.90 to 17.55) | 346.11(104.16 to 822.43) | 263.05(132.09 to 467.82) | 0.00(0.00 to 0.00) | 0.00(0.00 to 0.00) | 0.02(0.00 to 0.07) | 0.02(0.01 to 0.04) |
| East Asia | 32.87(16.50 to 54.14) | 16.26(12.58 to 21.29) | 541.63(274.26 to 904.11) | 274.92(215.30 to 347.68) | 3.35(1.51 to 5.67) | 8.60(6.15 to 11.34) | 80.82(35.89 to 136.80) | 183.82(130.34 to 244.95) | 7.09(3.32 12.24) | 2.80(1.88 to 3.85) | 380.10(172.01 to 669.94) | 68.35(46.32 to 93.43) | 0.00(0.00 to 0.00) | 0.00(0.00 to 0.00) | 0.11(0.01 to 0.24) | 0.03(0.02 to 0.05) |
| Eastern Europe | 4.63(1.93 to 8.21) | 1.18(0.64 to 1.90) | 103.82(44.12 to 184.30) | 29.46(16.04 to 46.57) | 4.44(2.03 to 7.42) | 1.89(1.09 to 2.81) | 122.92(56.24 to 205.43) | 49.09(28.49 to 73.28) | 1.54(0.62 to 2.81) | 0.89(0.47 to 1.52) | 76.44(33.01 to 137.86) | 33.71(17.91 to 57.24) | 0.00(0.00 to 0.00) | 0.00(0.00 to 0.00) | 0.19(0.12 to 0.24) | 0.02(0.01 to 0.03) |
| Eastern Sub-Saharan Africa | 1.60(0.62 to 3.24) | 2.39(1.41 to 3.69) | 36.10(14.17 to 73.04) | 55.56(33.58 to 84.41) | 0.17(0.05 to 0.39) | 0.42(0.21 to 0.71) | 3.90(1.24 to 9.10) | 9.67(4.78 to 16.23) | 4.75(1.64 to 10.42) | 6.05(3.34 to 9.41) | 205.13(67.05 to 478.00) | 173.45(94.27 to 285.45) | 0.00(0.00 to 0.00) | 0.00(0.00 to 0.00) | 0.01(0.00 to 0.06) | 0.01(0.00 to 0.03) |
| High-income Asia Pacific | 1.39(0.50 to 2.63) | 0.89(0.55 to 1.34) | 33.24(11.69 to 63.77) | 24.94(16.10 to 35.94) | 2.55(0.91 to 4.63) | 2.56(1.71 to 3.63) | 55.92(20.46 to 99.92) | 50.75(34.50 to 71.43) | 2.80(0.82 to 5.77) | 1.64(0.89 to 2.63) | 48.48(15.39 to 96.53) | 23.41(13.10 to 37.15) | 0.00(0.00 to 0.00) | 0.00(0.00 to 0.00) | 0.01(0.01 to 0.03) | 0.01(0.01 to 0.02) |
| High-income North America | 2.18(0.68 to 4.42) | 1.09(0.51 to 1.85) | 66.60(21.16 to 131.85) | 29.51(13.64 to 49.38) | 4.35(1.62 to 8.55) | 1.41(0.67 to 2.36) | 105.54(39.36 to 207.03) | 30.01(14.41 to 50.47) | 1.34(0.39 to 2.91) | 0.34(0.15 to 0.65) | 25.28(7.77 to 53.87) | 6.27(2.89 to 11.96) | 0.00(0.00 to 0.00) | 0.00(0.00 to 0.00) | 0.02(0.01 to 0.03) | 0.01(0.01 to 0.02) |
| North Africa and Middle East | 5.87(4.40 to 7.70) | 5.95(4.34 to 7.73) | 133.44(101.99 to 170.80) | 148.76(111.34 to 190.83) | 2.74(1.85 to 3.68) | 3.58(2.64 to 4.60) | 67.44(46.24 to 91.87) | 83.09(60.92 to 107.52) | 8.26(5.64 to 11.63) | 4.92(3.40 to 6.90) | 466.69(309.58 to 683.41) | 163.18(109.75 to 232.00) | 0.00(0.00 to 0.00) | 0.00(0.00 to 0.00) | 0.02(0.01 to 0.03) | 0.02(0.01 to 0.03) |
| Oceania | 4.87(1.19 to 13.89) | 5.87(1.71 to 14.98) | 98.14(24.33 to 278.52) | 117.77(33.96 to 295.58) | 0.55(0.17 to 1.42) | 0.88(0.29 to 2.07) | 12.88(4.01 to 33.24) | 20.29(6.31 to 48.34) | 2.74(0.71 to 7.52) | 2.88(0.81 to 7.06) | 135.18(29.74 to 384.47) | 135.17(33.86 to 348.69) | 0.00(0.00 to 0.00) | 0.00(0.00 to 0.00) | 0.00(0.00 to 0.01) | 0.00(0.00 to 0.01) |
| South Asia | 18.20(7.86 to 31.94) | 27.21(19.77 to 34.71) | 369.68(163.70 to 652.33) | 555.77(424.16 to 698.89) | 0.67(0.28 to 1.25) | 1.76(1.22 to 2.36) | 16.08(6.82 to 29.74) | 42.00(28.98 to 56.18) | 8.47(3.45 to 16.00) | 9.87(7.12 to 12.69) | 455.72(181.23 to 878.43) | 380.07(267.23 to 501.46) | 0.00(0.00 to 0.00) | 0.00(0.00 to 0.00) | 0.02(0.00 to 0.07) | 0.02(0.01 to 0.04) |
| Southeast Asia | 6.27(2.98 to 11.11) | 5.00(3.61 to 6.61) | 134.05(65.00 to 236.47) | 112.80(83.30 to 148.10) | 1.83(0.81 to 3.22) | 2.79(1.90 to 3.83) | 44.99(20.08 to 79.16) | 63.77(43.71 to 88.01) | 5.36(2.54 to 9.90) | 4.36(2.77 to 6.44) | 257.62(116.45 to 495.03) | 116.84(76.05 to 171.18) | 0.00(0.00 to 0.00) | 0.00(0.00 to 0.00) | 0.01(0.00 to 0.01) | 0.01(0.00 to 0.02) |
| Southern Latin America | 2.69(1.12 to 4.91) | 2.57(1.53 to 3.73) | 56.99(24.07 to 103.70) | 51.70(30.90 to 75.41) | 2.88(1.14 to 5.22) | 2.40(1.56 to 3.43) | 72.92(28.75 to 133.49) | 54.63(35.09 to 78.85) | 3.28(1.32 to 6.39) | 3.49(1.87 to 5.80) | 84.88(34.03 to 163.37) | 61.24(33.39 to 103.03) | 0.00(0.00 to 0.00) | 0.00(0.00 to 0.00) | 0.01(0.00 to 0.02) | 0.01(0.00 to 0.03) |
| Southern Sub-Saharan Africa | 5.57(3.94 to 7.72) | 5.40(3.78 to 7.29) | 139.98(101.78 to 186.16) | 137.30(97.12 to 183.77) | 2.63(1.68 to 4.00) | 2.92(2.06 to 4.03) | 67.11(43.61 to 101.05) | 68.70(48.18 to 94.80) | 10.84(7.38 to 15.19) | 10.35(6.49 to 15.07) | 450.02(305.28 to 638.17) | 324.88(206.05 to 475.87) | 0.00(0.00 to 0.00) | 0.00(0.00 to 0.00) | 0.05(0.02 to 0.08) | 0.04(0.02 to 0.06) |
| Tropical Latin America | 4.16(1.96 to 7.37) | 2.29(1.43 to 3.40) | 80.22(37.27 to 141.18) | 45.19(28.71 to 66.32) | 1.32(0.58 to 2.40) | 1.21(0.78 to 1.71) | 32.10(14.07 to 58.14) | 27.41(17.74 to 38.64) | 3.99(1.76 to 7.42) | 2.34(1.33 to 3.79) | 157.22(70.25 to 290.22) | 54.06(31.77 to 87.92) | 0.00(0.00 to 0.00) | 0.00(0.00 to 0.00) | 0.01(0.00 to 0.03) | 0.01(0.01 to 0.02) |
| Western Europe | 3.12(1.20 to 5.65) | 1.30(0.80 to 1.92) | 69.74(26.79 to 125.80) | 30.79(18.75 to 45.04) | 4.63(1.98 to 7.70) | 2.25(1.47 to 3.25) | 112.23(47.90 to 186.14) | 50.52(33.01 to 73.09) | 1.95(0.65 to 3.81) | 0.67(0.37 to 1.12) | 31.59(10.80 to 61.91) | 9.57(5.43 to 15.85) | 0.00(0.00 to 0.00) | 0.00(0.00 to 0.00) | 0.02(0.01 to 0.03) | 0.01(0.01 to 0.02) |
| Western Sub-Saharan Africa | 2.88(1.34 to 5.15) | 4.55(3.03 to 6.53) | 68.66(32.43 to 125.56) | 110.39(74.05 to 158.26) | 0.47(0.18 to 0.93) | 1.27(0.72 to 1.88) | 10.65(4.07 to 21.22) | 27.37(15.42 to 40.95) | 10.41(4.27 to 20.82) | 14.82(9.21 to 22.67) | 486.08(184.72 to 1048.25) | 535.71(304.34 to 867.25) | 0.00(0.00 to 0.00) | 0.00(0.00 to 0.00) | 0.01(0.00 to 0.03) | 0.01(0.01 to 0.02) |

Abbreviations: ASDR age-standardized death rate; DALY disability-adjusted life-year; TBL cancer tracheal, bronchus, and lung cancer; LRIs lower respiratory infections; URIs upper respiratory infections; UI uncertainty interval.

**Table S5 National trend in age-standardized burden of respiratory diseases attributable to ambient particulate matter pollution from 1990 to 2019.**

| **Nation** | **COPD** | | | | | | **LRIs** | | | | | | **TBL cancer** | | | | | | **URIs** | | | | | |
| --- | --- | --- | --- | --- | --- | --- | --- | --- | --- | --- | --- | --- | --- | --- | --- | --- | --- | --- | --- | --- | --- | --- | --- | --- |
|  | ASDR, per 100,000 populations | | Percent change in rate from 1990 to 2017 | Age-standardized DALY rate, per 100,000 populations | | Percent change in rate from 1990 to 2017 | ASDR, per 100,000 populations | | Percent change in rate from 1990 to 2017 | Age-standardized DALY rate, per 100,000 populations | | Percent change in rate from 1990 to 2017 | ASDR, per 100,000 populations | | Percent change in rate from 1990 to 2017 | Age-standardized DALY rate, per 100,000 populations | | Percent change in rate from 1990 to 2017 | ASDR, per 100,000 populations | | Percent change in rate from 1990 to 2017 | Age-standardized DALY rate, per 100,000 populations | | Percent change in rate from 1990 to 2017 |
|  | 1990 | 2019 |  | 1990 | 2019 |  | 1990 | 2019 |  | 1990 | 2019 |  | 1990 | 2019 |  | 1990 | 2019 |  | 1990 | 2019 |  | 1990 | 2019 |  |
| Afghanistan | 3.01 | 5.82 | 93.50 | 67.08 | 134.29 | 100.18 | 6.29 | 6.92 | 9.96 | 389.32 | 286.35 | -26.45 | 0.49 | 1.10 | 126.03 | 11.68 | 26.42 | 126.19 | 0.00001 | 0.00001 | 63.12659 | 0.00326 | 0.00794 | 143.47 |
| Albania | 5.35 | 1.78 | -66.71 | 93.79 | 36.29 | -61.31 | 7.25 | 1.33 | -81.70 | 365.40 | 51.08 | -86.02 | 3.00 | 3.20 | 6.67 | 72.21 | 73.18 | 1.34 | 0.00053 | 0.00039 | -27.87899 | 0.05145 | 0.03989 | -22.48 |
| Algeria | 5.42 | 4.40 | -18.87 | 102.79 | 96.17 | -6.44 | 7.20 | 3.86 | -46.37 | 267.65 | 84.45 | -68.45 | 2.11 | 2.16 | 2.26 | 47.18 | 47.69 | 1.09 | 0.00001 | 0.00000 | -22.85170 | 0.00945 | 0.01790 | 89.38 |
| American Samoa | 2.99 | 1.52 | -49.09 | 58.09 | 30.47 | -47.54 | 1.28 | 0.78 | -39.35 | 35.87 | 20.65 | -42.43 | 1.16 | 1.01 | -12.58 | 26.25 | 22.88 | -12.84 | 0.00000 | 0.00000 | -39.46510 | 0.00260 | 0.00333 | 28.03 |
| Andorra | 2.85 | 1.05 | -63.06 | 58.63 | 24.26 | -58.62 | 1.15 | 0.43 | -62.45 | 19.43 | 6.10 | -68.59 | 4.23 | 1.87 | -55.88 | 97.90 | 42.75 | -56.33 | 0.00000 | 0.00000 | -67.12487 | 0.00999 | 0.00922 | -7.69 |
| Angola | 2.25 | 4.29 | 90.80 | 50.04 | 99.96 | 99.75 | 6.25 | 10.09 | 61.36 | 301.76 | 282.53 | -6.37 | 0.41 | 1.59 | 283.39 | 10.53 | 38.35 | 264.27 | 0.00017 | 0.00012 | -30.46492 | 0.01784 | 0.02317 | 29.84 |
| Antigua and Barbuda | 0.88 | 0.98 | 11.34 | 17.76 | 20.15 | 13.46 | 3.30 | 3.21 | -2.80 | 71.35 | 63.08 | -11.59 | 1.19 | 1.17 | -1.65 | 28.04 | 25.58 | -8.75 | 0.00001 | 0.00000 | -47.46286 | 0.00948 | 0.00998 | 5.22 |
| Argentina | 2.71 | 2.57 | -5.43 | 58.63 | 53.06 | -9.50 | 2.29 | 4.25 | 85.85 | 62.96 | 75.73 | 20.29 | 3.08 | 2.38 | -22.65 | 79.32 | 56.01 | -29.38 | 0.00001 | 0.00000 | -66.52016 | 0.00772 | 0.00935 | 21.21 |
| Armenia | 8.15 | 6.63 | -18.59 | 166.31 | 122.43 | -26.38 | 4.90 | 2.85 | -41.89 | 354.36 | 117.42 | -66.86 | 5.89 | 6.43 | 9.09 | 165.67 | 156.22 | -5.70 | 0.00092 | 0.00013 | -85.90209 | 0.08805 | 0.02164 | -75.42 |
| Austria | 2.27 | 1.17 | -48.61 | 54.08 | 29.85 | -44.81 | 1.28 | 0.31 | -75.75 | 25.68 | 5.01 | -80.50 | 4.10 | 2.03 | -50.47 | 100.51 | 47.25 | -52.98 | 0.00004 | 0.00001 | -77.45828 | 0.01583 | 0.01060 | -33.04 |
| Azerbaijan | 3.88 | 4.19 | 8.05 | 92.58 | 87.00 | -6.03 | 10.08 | 4.67 | -53.64 | 823.92 | 272.79 | -66.89 | 3.00 | 3.86 | 28.33 | 86.43 | 99.94 | 15.63 | 0.00384 | 0.00340 | -11.32976 | 0.34388 | 0.30776 | -10.50 |
| Bahamas | 1.12 | 0.99 | -11.93 | 24.71 | 22.26 | -9.92 | 3.53 | 2.21 | -37.56 | 95.04 | 53.50 | -43.71 | 1.91 | 1.56 | -18.22 | 48.08 | 38.04 | -20.88 | 0.00002 | 0.00001 | -62.29026 | 0.01091 | 0.00923 | -15.45 |
| Bahrain | 19.69 | 9.56 | -51.44 | 368.00 | 181.96 | -50.55 | 7.90 | 5.73 | -27.50 | 152.36 | 85.32 | -44.00 | 12.44 | 6.25 | -49.74 | 243.91 | 110.80 | -54.58 | 0.00000 | 0.00000 | -62.74539 | 0.02342 | 0.02681 | 14.44 |
| Bangladesh | 9.78 | 11.47 | 17.30 | 212.28 | 259.63 | 22.31 | 5.60 | 5.89 | 5.04 | 327.46 | 232.75 | -28.92 | 0.47 | 1.21 | 156.97 | 11.50 | 28.03 | 143.75 | 0.00021 | 0.00006 | -69.93066 | 0.02259 | 0.01885 | -16.55 |
| Barbados | 1.05 | 1.18 | 12.86 | 21.59 | 24.59 | 13.91 | 3.31 | 3.83 | 15.69 | 79.10 | 71.91 | -9.09 | 1.45 | 1.42 | -1.87 | 33.76 | 31.78 | -5.87 | 0.00002 | 0.00001 | -55.24425 | 0.01346 | 0.01209 | -10.12 |
| Belarus | 7.47 | 1.52 | -79.69 | 151.19 | 36.55 | -75.82 | 1.67 | 0.67 | -59.55 | 81.66 | 21.86 | -73.23 | 5.37 | 2.59 | -51.73 | 149.90 | 66.73 | -55.48 | 0.00103 | 0.00008 | -92.47023 | 0.09943 | 0.01358 | -86.34 |
| Belgium | 4.74 | 1.99 | -57.96 | 103.75 | 49.38 | -52.40 | 2.43 | 1.26 | -48.35 | 37.26 | 16.80 | -54.90 | 7.27 | 3.10 | -57.42 | 171.53 | 69.86 | -59.27 | 0.00001 | 0.00001 | -60.42012 | 0.01299 | 0.01009 | -22.32 |
| Belize | 1.78 | 2.82 | 58.62 | 37.62 | 61.02 | 62.21 | 4.58 | 4.56 | -0.26 | 144.95 | 107.74 | -25.67 | 0.86 | 1.75 | 102.81 | 20.07 | 42.25 | 110.58 | 0.00002 | 0.00002 | -5.67635 | 0.00668 | 0.01080 | 61.69 |
| Benin | 2.43 | 3.24 | 33.39 | 56.10 | 83.20 | 48.30 | 8.02 | 11.10 | 38.54 | 350.62 | 368.20 | 5.02 | 0.37 | 0.86 | 131.38 | 8.47 | 19.09 | 125.28 | 0.00005 | 0.00004 | -31.97300 | 0.00634 | 0.00761 | 20.15 |
| Bermuda | 0.73 | 0.28 | -61.97 | 15.11 | 6.07 | -59.81 | 1.11 | 0.31 | -72.26 | 21.63 | 5.56 | -74.32 | 2.46 | 0.87 | -64.86 | 56.31 | 18.73 | -66.74 | 0.00000 | 0.00000 | -22.28743 | 0.00538 | 0.00562 | 4.41 |
| Bhutan | 6.85 | 18.40 | 168.71 | 142.18 | 347.47 | 144.38 | 2.67 | 4.45 | 66.77 | 161.94 | 150.50 | -7.07 | 0.20 | 1.07 | 435.95 | 4.92 | 23.70 | 381.41 | 0.00014 | 0.00008 | -41.80144 | 0.01385 | 0.01457 | 5.16 |
| Bolivia (Plurinational State of) | 7.94 | 5.79 | -27.16 | 137.44 | 92.58 | -32.64 | 23.28 | 10.09 | -56.67 | 1140.84 | 260.24 | -77.19 | 1.69 | 2.01 | 19.47 | 39.08 | 42.82 | 9.58 | 0.00100 | 0.00018 | -81.93881 | 0.09487 | 0.02432 | -74.36 |
| Bosnia and Herzegovina | 5.35 | 3.60 | -32.70 | 112.98 | 80.52 | -28.73 | 2.04 | 1.17 | -42.94 | 50.59 | 23.95 | -52.66 | 4.83 | 6.80 | 40.74 | 125.20 | 164.75 | 31.59 | 0.00000 | 0.00000 | -23.94325 | 0.00568 | 0.00922 | 62.30 |
| Botswana | 5.22 | 6.00 | 15.01 | 118.81 | 149.24 | 25.61 | 9.34 | 13.24 | 41.66 | 284.31 | 408.64 | 43.73 | 1.21 | 3.10 | 155.69 | 30.39 | 75.02 | 146.89 | 0.00028 | 0.00026 | -7.83293 | 0.03106 | 0.03565 | 14.75 |
| Brazil | 4.25 | 2.30 | -45.81 | 81.78 | 45.54 | -44.31 | 4.06 | 2.36 | -41.93 | 160.15 | 54.42 | -66.02 | 1.34 | 1.21 | -9.40 | 32.54 | 27.40 | -15.80 | 0.00010 | 0.00004 | -55.48775 | 0.01486 | 0.01360 | -8.44 |
| Brunei Darussalam | 4.28 | 1.77 | -58.63 | 71.71 | 30.25 | -57.81 | 1.56 | 1.38 | -11.56 | 29.41 | 23.32 | -20.69 | 2.26 | 1.82 | -19.31 | 46.64 | 35.18 | -24.57 | 0.00004 | 0.00001 | -61.04952 | 0.00846 | 0.00853 | 0.81 |
| Bulgaria | 5.40 | 2.54 | -52.93 | 120.64 | 64.82 | -46.27 | 6.27 | 1.66 | -73.46 | 236.50 | 55.04 | -76.73 | 4.42 | 4.52 | 2.36 | 125.81 | 123.84 | -1.56 | 0.00009 | 0.00001 | -83.72994 | 0.01422 | 0.00669 | -52.99 |
| Burkina Faso | 1.16 | 1.80 | 55.67 | 27.20 | 46.69 | 71.64 | 6.84 | 9.78 | 42.86 | 296.45 | 373.48 | 25.98 | 0.24 | 0.57 | 134.30 | 5.53 | 12.79 | 131.49 | 0.00008 | 0.00006 | -24.11683 | 0.00923 | 0.00896 | -2.95 |
| Burundi | 2.96 | 2.82 | -4.51 | 66.55 | 64.12 | -3.66 | 6.22 | 5.56 | -10.61 | 249.45 | 155.72 | -37.58 | 0.24 | 0.29 | 21.66 | 5.73 | 6.68 | 16.63 | 0.00019 | 0.00010 | -46.81628 | 0.01841 | 0.01107 | -39.85 |
| Cabo Verde | 3.97 | 4.82 | 21.49 | 93.88 | 125.32 | 33.49 | 5.27 | 14.82 | 180.93 | 196.99 | 306.03 | 55.35 | 0.71 | 4.05 | 470.37 | 16.57 | 85.34 | 414.98 | 0.00002 | 0.00001 | -26.07803 | 0.00520 | 0.01455 | 180.07 |
| Cambodia | 2.44 | 3.65 | 49.47 | 51.43 | 78.00 | 51.67 | 8.20 | 8.46 | 3.17 | 422.10 | 245.31 | -41.88 | 0.77 | 1.77 | 131.28 | 18.47 | 40.54 | 119.48 | 0.00001 | 0.00001 | -51.71381 | 0.00290 | 0.00437 | 50.48 |
| Cameroon | 5.46 | 6.04 | 10.58 | 131.14 | 166.17 | 26.71 | 15.00 | 21.21 | 41.42 | 547.59 | 667.39 | 21.88 | 0.98 | 2.40 | 144.94 | 22.61 | 53.99 | 138.79 | 0.00006 | 0.00005 | -19.49304 | 0.01073 | 0.02030 | 89.24 |
| Canada | 1.42 | 0.62 | -56.62 | 31.32 | 13.77 | -56.03 | 0.92 | 0.25 | -72.52 | 14.25 | 3.83 | -73.14 | 2.91 | 1.18 | -59.56 | 69.49 | 24.70 | -64.46 | 0.00001 | 0.00000 | -55.11018 | 0.01055 | 0.00919 | -12.89 |
| Central African Republic | 3.59 | 4.58 | 27.63 | 81.82 | 105.50 | 28.94 | 8.66 | 10.83 | 25.09 | 372.38 | 406.06 | 9.04 | 0.50 | 0.67 | 32.10 | 13.35 | 17.59 | 31.74 | 0.00027 | 0.00017 | -35.71116 | 0.02790 | 0.02122 | -23.95 |
| Chad | 1.88 | 3.04 | 62.25 | 42.91 | 71.95 | 67.68 | 6.64 | 10.66 | 60.53 | 299.54 | 399.05 | 33.22 | 0.24 | 0.68 | 186.71 | 5.47 | 15.16 | 177.15 | 0.00006 | 0.00004 | -41.10526 | 0.00710 | 0.00605 | -14.73 |
| Chile | 3.13 | 2.87 | -8.34 | 60.98 | 53.16 | -12.82 | 7.70 | 2.40 | -68.87 | 167.12 | 37.39 | -77.63 | 2.30 | 2.58 | 12.11 | 54.07 | 53.85 | -0.41 | 0.00002 | 0.00001 | -69.42382 | 0.01238 | 0.01598 | 29.08 |
| China | 33.82 | 16.59 | -50.94 | 554.81 | 278.62 | -49.78 | 7.18 | 2.73 | -61.95 | 386.84 | 67.45 | -82.56 | 3.38 | 8.75 | 159.20 | 81.37 | 186.77 | 129.54 | 0.00116 | 0.00016 | -85.90142 | 0.10800 | 0.02743 | -74.60 |
| Colombia | 5.55 | 4.30 | -22.64 | 106.78 | 76.94 | -27.95 | 3.79 | 1.69 | -55.32 | 153.33 | 55.42 | -63.86 | 1.87 | 1.70 | -9.01 | 43.09 | 36.42 | -15.47 | 0.00034 | 0.00010 | -70.57494 | 0.03723 | 0.02100 | -43.60 |
| Comoros | 1.39 | 1.84 | 32.66 | 30.03 | 43.10 | 43.50 | 3.90 | 5.09 | 30.29 | 175.53 | 157.32 | -10.37 | 0.17 | 0.39 | 131.61 | 3.87 | 8.85 | 128.74 | 0.00011 | 0.00006 | -47.70107 | 0.01141 | 0.00940 | -17.61 |
| Congo | 5.43 | 6.83 | 25.67 | 122.17 | 160.41 | 31.30 | 11.12 | 14.05 | 26.32 | 360.29 | 325.28 | -9.72 | 1.21 | 2.68 | 121.47 | 30.91 | 64.04 | 107.19 | 0.00016 | 0.00011 | -33.65104 | 0.02164 | 0.02841 | 31.30 |
| Cook Islands | 1.80 | 0.72 | -60.04 | 42.17 | 20.60 | -51.16 | 2.21 | 0.93 | -57.66 | 68.24 | 20.08 | -70.57 | 1.25 | 0.94 | -24.76 | 28.22 | 20.90 | -25.94 | 0.00000 | 0.00000 | -59.60056 | 0.00290 | 0.00438 | 51.40 |
| Costa Rica | 2.51 | 2.74 | 9.50 | 45.06 | 46.12 | 2.36 | 1.68 | 1.17 | -30.55 | 47.89 | 27.97 | -41.59 | 0.99 | 1.18 | 19.32 | 22.05 | 24.75 | 12.21 | 0.00000 | 0.00001 | 153.84539 | 0.00552 | 0.00951 | 72.11 |
| Croatia | 2.86 | 2.32 | -19.01 | 71.27 | 53.64 | -24.74 | 2.17 | 0.61 | -71.78 | 49.44 | 11.17 | -77.41 | 6.66 | 4.34 | -34.84 | 176.30 | 105.38 | -40.23 | 0.00000 | 0.00000 | 520.41054 | 0.00718 | 0.00634 | -11.65 |
| Cuba | 2.05 | 2.51 | 22.18 | 46.01 | 57.92 | 25.90 | 3.48 | 3.10 | -10.86 | 74.16 | 54.14 | -27.00 | 4.31 | 4.18 | -3.09 | 98.32 | 93.28 | -5.13 | 0.00000 | 0.00000 | 16.61733 | 0.00988 | 0.01035 | 4.76 |
| Cyprus | 5.22 | 2.75 | -47.24 | 84.01 | 49.01 | -41.66 | 2.48 | 0.89 | -64.14 | 37.33 | 11.25 | -69.86 | 2.44 | 2.62 | 7.28 | 53.64 | 55.51 | 3.50 | 0.00000 | 0.00000 | -51.88544 | 0.00868 | 0.00937 | 7.88 |
| Czechia | 3.39 | 1.86 | -45.22 | 84.01 | 49.20 | -41.44 | 2.52 | 1.35 | -46.64 | 60.14 | 24.97 | -58.48 | 7.99 | 3.49 | -56.30 | 210.80 | 79.42 | -62.33 | 0.00020 | 0.00002 | -90.56335 | 0.02474 | 0.00737 | -70.21 |
| Cote d'Ivoire | 3.85 | 4.42 | 14.90 | 88.14 | 109.98 | 24.79 | 12.58 | 16.06 | 27.71 | 502.78 | 517.31 | 2.89 | 0.71 | 1.54 | 118.34 | 16.45 | 34.49 | 109.61 | 0.00008 | 0.00005 | -29.00988 | 0.01030 | 0.01250 | 21.37 |
| Democratic People's Republic of Korea | 18.47 | 19.52 | 5.71 | 342.97 | 375.98 | 9.63 | 5.64 | 4.23 | -24.96 | 294.88 | 108.47 | -63.22 | 2.03 | 3.94 | 94.09 | 52.57 | 98.52 | 87.43 | 0.00088 | 0.00022 | -75.32246 | 0.08197 | 0.02704 | -67.02 |
| Democratic Republic of the Congo | 3.59 | 4.57 | 27.51 | 74.10 | 100.92 | 36.19 | 9.08 | 9.01 | -0.80 | 352.30 | 238.66 | -32.26 | 0.59 | 0.79 | 34.37 | 14.09 | 19.44 | 38.03 | 0.00017 | 0.00008 | -55.20858 | 0.01820 | 0.01357 | -25.43 |
| Denmark | 3.85 | 1.85 | -52.00 | 97.08 | 42.21 | -56.52 | 1.85 | 0.74 | -59.86 | 28.16 | 9.29 | -67.02 | 6.01 | 2.28 | -62.02 | 146.55 | 48.46 | -66.93 | 0.00000 | 0.00000 | -50.66354 | 0.01114 | 0.00914 | -17.96 |
| Djibouti | 3.06 | 4.89 | 59.81 | 70.49 | 122.22 | 73.39 | 9.26 | 16.88 | 82.38 | 368.52 | 514.15 | 39.52 | 0.57 | 2.17 | 279.17 | 13.53 | 49.53 | 266.02 | 0.00027 | 0.00036 | 32.74738 | 0.02788 | 0.04849 | 73.96 |
| Dominica | 1.69 | 2.09 | 23.33 | 33.21 | 41.35 | 24.51 | 3.27 | 3.33 | 1.76 | 74.96 | 83.97 | 12.01 | 1.57 | 2.21 | 40.74 | 36.98 | 50.41 | 36.33 | 0.00003 | 0.00003 | -7.13364 | 0.00970 | 0.01223 | 26.07 |
| Dominican Republic | 0.99 | 1.86 | 88.62 | 18.61 | 38.02 | 104.32 | 1.93 | 2.00 | 3.70 | 94.57 | 55.74 | -41.06 | 0.61 | 1.79 | 192.96 | 14.26 | 41.23 | 189.17 | 0.00001 | 0.00001 | -6.04116 | 0.00370 | 0.00849 | 129.42 |
| Ecuador | 3.28 | 3.01 | -8.10 | 51.37 | 43.81 | -14.73 | 6.44 | 4.28 | -33.56 | 218.96 | 98.57 | -54.98 | 0.95 | 1.47 | 54.95 | 21.52 | 29.79 | 38.45 | 0.00037 | 0.00011 | -71.19905 | 0.03898 | 0.01881 | -51.74 |
| Egypt | 9.42 | 10.06 | 6.79 | 221.23 | 270.34 | 22.20 | 22.80 | 9.74 | -57.31 | 1448.85 | 337.57 | -76.70 | 1.76 | 2.78 | 58.38 | 45.47 | 69.99 | 53.94 | 0.00001 | 0.00001 | -62.34416 | 0.02313 | 0.02245 | -2.93 |
| El Salvador | 2.30 | 2.63 | 14.58 | 42.25 | 47.78 | 13.09 | 3.83 | 3.96 | 3.35 | 143.76 | 93.51 | -34.95 | 0.60 | 1.43 | 137.22 | 14.56 | 32.15 | 120.75 | 0.00005 | 0.00002 | -65.93368 | 0.00878 | 0.01015 | 15.62 |
| Equatorial Guinea | 2.95 | 7.68 | 160.64 | 66.09 | 174.86 | 164.57 | 6.71 | 15.99 | 138.32 | 296.56 | 345.54 | 16.52 | 0.42 | 3.84 | 806.57 | 10.97 | 87.66 | 699.27 | 0.00015 | 0.00009 | -38.87004 | 0.01632 | 0.03572 | 118.92 |
| Eritrea | 2.74 | 4.72 | 72.17 | 69.05 | 118.23 | 71.22 | 9.85 | 17.05 | 73.16 | 439.29 | 480.62 | 9.41 | 0.26 | 0.81 | 215.04 | 6.70 | 19.90 | 197.12 | 0.00021 | 0.00015 | -28.15471 | 0.02091 | 0.02031 | -2.83 |
| Estonia | 0.74 | 0.21 | -71.08 | 18.90 | 5.31 | -71.93 | 0.63 | 0.17 | -72.94 | 26.21 | 5.24 | -80.01 | 2.52 | 0.75 | -70.46 | 67.64 | 17.27 | -74.47 | 0.00002 | 0.00000 | -95.74978 | 0.00538 | 0.00349 | -35.00 |
| Eswatini | 5.00 | 5.56 | 11.23 | 118.52 | 141.34 | 19.26 | 8.14 | 10.89 | 33.75 | 315.16 | 378.50 | 20.10 | 0.96 | 2.06 | 115.04 | 23.98 | 51.57 | 115.04 | 0.00026 | 0.00022 | -17.89990 | 0.02717 | 0.02768 | 1.88 |
| Ethiopia | 1.40 | 2.32 | 65.21 | 33.46 | 50.14 | 49.85 | 4.61 | 5.42 | 17.76 | 200.38 | 142.47 | -28.90 | 0.12 | 0.33 | 174.24 | 2.82 | 7.07 | 150.83 | 0.00022 | 0.00014 | -37.88151 | 0.02062 | 0.01472 | -28.64 |
| Fiji | 1.89 | 1.95 | 2.86 | 37.23 | 38.09 | 2.31 | 1.64 | 1.90 | 16.28 | 52.88 | 62.55 | 18.28 | 0.37 | 0.73 | 94.39 | 8.68 | 16.54 | 90.49 | 0.00000 | 0.00000 | 73.44777 | 0.00183 | 0.00411 | 125.28 |
| Finland | 0.64 | 0.18 | -71.21 | 16.97 | 4.96 | -70.80 | 1.29 | 0.06 | -95.31 | 19.05 | 0.95 | -95.02 | 1.52 | 0.38 | -75.00 | 35.55 | 8.00 | -77.51 | 0.00000 | 0.00000 | 40.07669 | 0.00754 | 0.00643 | -14.63 |
| France | 2.43 | 0.69 | -71.77 | 43.89 | 15.53 | -64.61 | 1.87 | 0.59 | -68.25 | 27.90 | 7.84 | -71.91 | 4.16 | 2.42 | -41.76 | 106.71 | 59.88 | -43.88 | 0.00018 | 0.00003 | -83.23202 | 0.02626 | 0.01235 | -52.98 |
| Gabon | 5.20 | 5.84 | 12.26 | 116.82 | 138.47 | 18.53 | 12.08 | 13.54 | 12.07 | 396.56 | 308.28 | -22.26 | 1.73 | 4.23 | 143.64 | 44.02 | 101.29 | 130.11 | 0.00022 | 0.00011 | -48.21345 | 0.03023 | 0.03485 | 15.25 |
| Gambia | 2.71 | 4.79 | 76.70 | 64.74 | 118.74 | 83.42 | 8.47 | 15.10 | 78.26 | 310.56 | 365.16 | 17.58 | 0.27 | 0.76 | 179.81 | 6.28 | 17.08 | 171.86 | 0.00005 | 0.00003 | -40.82969 | 0.00552 | 0.00637 | 15.34 |
| Georgia | 1.65 | 1.84 | 11.47 | 37.54 | 49.58 | 32.07 | 3.82 | 1.23 | -67.67 | 273.78 | 39.88 | -85.43 | 3.13 | 3.55 | 13.34 | 90.08 | 95.70 | 6.24 | 0.00009 | 0.00001 | -86.07883 | 0.01074 | 0.00501 | -53.36 |
| Germany | 3.19 | 1.33 | -58.27 | 78.14 | 37.34 | -52.22 | 1.54 | 0.59 | -61.87 | 27.35 | 9.09 | -66.77 | 4.24 | 2.33 | -44.90 | 105.72 | 53.75 | -49.15 | 0.00001 | 0.00000 | -58.72002 | 0.01360 | 0.01030 | -24.28 |
| Ghana | 4.06 | 6.19 | 52.45 | 92.17 | 156.36 | 69.64 | 14.69 | 19.48 | 32.62 | 470.93 | 523.57 | 11.18 | 0.67 | 1.63 | 142.52 | 16.07 | 37.68 | 134.51 | 0.00003 | 0.00013 | 354.78823 | 0.00663 | 0.02153 | 224.60 |
| Greece | 2.24 | 1.75 | -21.73 | 55.63 | 42.71 | -23.23 | 1.44 | 1.30 | -9.56 | 25.91 | 19.46 | -24.88 | 5.73 | 3.79 | -33.82 | 132.63 | 86.21 | -35.00 | 0.00000 | 0.00000 | -67.02795 | 0.01105 | 0.01097 | -0.70 |
| Greenland | 2.90 | 1.32 | -54.44 | 60.41 | 28.54 | -52.75 | 1.29 | 0.50 | -61.60 | 30.22 | 8.72 | -71.13 | 3.17 | 2.33 | -26.48 | 78.58 | 52.74 | -32.88 | 0.00000 | 0.00000 | 5.47536 | 0.00686 | 0.00835 | 21.60 |
| Grenada | 1.80 | 2.05 | 13.88 | 37.71 | 45.46 | 20.56 | 6.18 | 5.77 | -6.58 | 153.78 | 123.61 | -19.62 | 1.41 | 2.08 | 47.69 | 34.04 | 48.75 | 43.19 | 0.00001 | 0.00001 | -26.92505 | 0.00726 | 0.01135 | 56.38 |
| Guam | 2.45 | 0.90 | -63.24 | 44.98 | 19.98 | -55.58 | 1.45 | 0.64 | -56.07 | 36.34 | 22.04 | -39.34 | 2.28 | 1.55 | -32.03 | 50.21 | 36.62 | -27.06 | 0.00000 | 0.00000 | 102.76347 | 0.00439 | 0.00522 | 18.80 |
| Guatemala | 2.71 | 3.04 | 12.25 | 45.31 | 48.37 | 6.77 | 16.18 | 10.27 | -36.50 | 523.92 | 280.55 | -46.45 | 0.54 | 0.93 | 72.76 | 12.35 | 20.31 | 64.42 | 0.00061 | 0.00017 | -72.79263 | 0.05707 | 0.02151 | -62.30 |
| Guinea | 2.30 | 3.44 | 49.89 | 52.36 | 85.95 | 64.15 | 9.55 | 13.09 | 37.11 | 490.80 | 454.56 | -7.38 | 0.31 | 0.68 | 123.54 | 7.25 | 16.29 | 124.67 | 0.00008 | 0.00004 | -47.69906 | 0.00893 | 0.00696 | -22.02 |
| Guinea-Bissau | 3.52 | 4.29 | 21.85 | 84.81 | 109.79 | 29.46 | 10.50 | 13.60 | 29.43 | 401.67 | 360.19 | -10.33 | 0.54 | 1.00 | 83.83 | 13.14 | 23.48 | 78.64 | 0.00008 | 0.00004 | -52.14467 | 0.00933 | 0.00769 | -17.58 |
| Guyana | 1.98 | 1.99 | 0.24 | 39.80 | 43.29 | 8.76 | 6.52 | 5.16 | -20.95 | 181.47 | 126.47 | -30.31 | 0.91 | 1.17 | 28.15 | 22.49 | 28.77 | 27.91 | 0.00001 | 0.00001 | -23.83904 | 0.00864 | 0.01070 | 23.90 |
| Haiti | 1.69 | 2.63 | 55.37 | 33.46 | 51.08 | 52.65 | 3.98 | 4.20 | 5.68 | 203.44 | 158.50 | -22.09 | 0.33 | 0.58 | 73.73 | 8.38 | 13.67 | 63.21 | 0.00004 | 0.00002 | -36.19604 | 0.00543 | 0.00622 | 14.60 |
| Honduras | 3.11 | 6.53 | 109.88 | 59.52 | 117.03 | 96.62 | 1.93 | 1.83 | -5.21 | 91.03 | 45.12 | -50.44 | 0.59 | 1.93 | 225.68 | 14.50 | 44.07 | 204.00 | 0.00055 | 0.00019 | -64.45578 | 0.05150 | 0.02298 | -55.39 |
| Hungary | 5.63 | 3.11 | -44.78 | 128.22 | 82.40 | -35.74 | 1.47 | 0.55 | -62.41 | 55.51 | 13.69 | -75.34 | 7.16 | 5.57 | -22.18 | 194.22 | 141.16 | -27.32 | 0.00028 | 0.00003 | -89.93130 | 0.03165 | 0.00792 | -74.98 |
| India | 21.57 | 29.42 | 36.39 | 419.79 | 598.44 | 42.56 | 9.87 | 10.57 | 7.17 | 511.34 | 400.28 | -21.72 | 0.67 | 1.71 | 156.65 | 15.93 | 40.48 | 154.08 | 0.00018 | 0.00008 | -55.98317 | 0.02161 | 0.02059 | -4.68 |
| Indonesia | 5.85 | 5.55 | -5.16 | 124.19 | 119.10 | -4.09 | 4.75 | 2.64 | -44.43 | 268.11 | 76.25 | -71.56 | 1.35 | 2.80 | 107.67 | 32.70 | 63.55 | 94.34 | 0.00001 | 0.00000 | -68.47630 | 0.00665 | 0.00954 | 43.53 |
| Iran (Islamic Republic of) | 5.13 | 4.78 | -6.79 | 116.91 | 117.93 | 0.87 | 5.62 | 3.09 | -45.02 | 263.47 | 68.53 | -73.99 | 2.31 | 2.84 | 23.08 | 53.19 | 62.60 | 17.71 | 0.00008 | 0.00006 | -24.56967 | 0.02010 | 0.02409 | 19.82 |
| Iraq | 3.08 | 2.82 | -8.57 | 87.21 | 73.05 | -16.24 | 7.19 | 2.91 | -59.56 | 408.58 | 98.07 | -76.00 | 3.46 | 5.02 | 45.15 | 82.91 | 113.76 | 37.21 | 0.00026 | 0.00003 | -89.33826 | 0.03989 | 0.02419 | -39.36 |
| Ireland | 4.05 | 1.03 | -74.51 | 82.54 | 21.76 | -73.63 | 3.05 | 0.59 | -80.76 | 41.76 | 6.91 | -83.45 | 3.36 | 1.22 | -63.81 | 75.69 | 25.06 | -66.89 | 0.00000 | 0.00000 | 492.43777 | 0.00852 | 0.00746 | -12.36 |
| Israel | 3.52 | 1.84 | -47.73 | 72.78 | 44.84 | -38.38 | 1.97 | 1.35 | -31.50 | 35.35 | 19.73 | -44.18 | 3.68 | 3.04 | -17.48 | 84.90 | 66.78 | -21.34 | 0.00003 | 0.00001 | -77.30733 | 0.01564 | 0.01449 | -7.34 |
| Italy | 3.61 | 1.51 | -58.21 | 78.93 | 32.79 | -58.45 | 1.53 | 0.53 | -65.24 | 30.44 | 7.94 | -73.92 | 6.27 | 2.88 | -53.99 | 153.50 | 61.78 | -59.75 | 0.00011 | 0.00001 | -93.01456 | 0.02360 | 0.01208 | -48.81 |
| Jamaica | 1.12 | 1.72 | 54.39 | 25.54 | 38.84 | 52.06 | 1.50 | 1.11 | -25.78 | 44.31 | 27.01 | -39.06 | 1.04 | 2.01 | 94.26 | 25.87 | 48.96 | 89.24 | 0.00000 | 0.00000 | 72.69004 | 0.00454 | 0.00753 | 65.62 |
| Japan | 1.02 | 0.64 | -37.96 | 25.44 | 17.10 | -32.78 | 2.64 | 1.41 | -46.67 | 39.13 | 19.95 | -49.01 | 2.24 | 1.98 | -11.47 | 46.39 | 38.12 | -17.82 | 0.00004 | 0.00000 | -96.19672 | 0.01309 | 0.01003 | -23.39 |
| Jordan | 4.91 | 2.49 | -49.38 | 110.67 | 67.57 | -38.95 | 4.85 | 2.91 | -39.91 | 171.42 | 80.33 | -53.14 | 2.46 | 3.02 | 22.59 | 58.83 | 67.23 | 14.28 | 0.00000 | 0.00000 | -66.19751 | 0.01025 | 0.01127 | 9.88 |
| Kazakhstan | 5.95 | 7.72 | 29.83 | 138.34 | 156.64 | 13.23 | 3.90 | 2.57 | -33.99 | 267.61 | 87.86 | -67.17 | 4.69 | 2.84 | -39.44 | 131.62 | 71.02 | -46.04 | 0.00013 | 0.00005 | -65.11385 | 0.01599 | 0.00942 | -41.10 |
| Kenya | 1.71 | 2.78 | 62.05 | 39.23 | 65.81 | 67.76 | 4.74 | 6.75 | 42.28 | 171.36 | 170.54 | -0.48 | 0.17 | 0.45 | 162.20 | 3.88 | 10.31 | 165.37 | 0.00006 | 0.00005 | -16.75378 | 0.00851 | 0.01139 | 33.77 |
| Kiribati | 2.28 | 2.52 | 10.58 | 50.48 | 52.17 | 3.35 | 1.90 | 1.86 | -2.36 | 77.19 | 55.66 | -27.90 | 0.45 | 0.63 | 41.02 | 11.78 | 16.05 | 36.24 | 0.00004 | 0.00003 | -29.42944 | 0.00502 | 0.00445 | -11.44 |
| Kuwait | 3.17 | 2.82 | -10.99 | 77.85 | 69.59 | -10.61 | 7.80 | 9.66 | 23.80 | 201.63 | 161.63 | -19.84 | 3.53 | 3.09 | -12.54 | 80.51 | 61.45 | -23.67 | 0.00000 | 0.00000 | 48.56010 | 0.02594 | 0.02834 | 9.29 |
| Kyrgyzstan | 9.77 | 5.87 | -39.97 | 209.74 | 120.14 | -42.72 | 5.26 | 1.59 | -69.85 | 397.77 | 82.52 | -79.25 | 2.37 | 1.64 | -30.82 | 67.22 | 40.78 | -39.33 | 0.00061 | 0.00042 | -30.91407 | 0.05633 | 0.04120 | -26.86 |
| Lao People's Democratic Republic | 4.02 | 4.65 | 15.86 | 87.45 | 101.18 | 15.70 | 7.01 | 5.16 | -26.42 | 428.06 | 178.09 | -58.39 | 0.77 | 1.52 | 96.96 | 19.37 | 35.35 | 82.52 | 0.00001 | 0.00001 | -53.78478 | 0.00297 | 0.00468 | 57.79 |
| Latvia | 2.08 | 0.65 | -68.82 | 50.65 | 16.00 | -68.42 | 1.35 | 0.59 | -56.39 | 54.10 | 19.38 | -64.18 | 4.76 | 2.02 | -57.58 | 129.55 | 49.31 | -61.94 | 0.00000 | 0.00000 | -51.21489 | 0.00613 | 0.00521 | -14.94 |
| Lebanon | 3.56 | 3.26 | -8.48 | 87.00 | 99.02 | 13.81 | 3.55 | 2.70 | -23.92 | 103.20 | 53.87 | -47.80 | 3.40 | 5.29 | 55.76 | 81.39 | 120.90 | 48.54 | 0.00000 | 0.00000 | -39.20868 | 0.01102 | 0.01538 | 39.60 |
| Lesotho | 6.36 | 9.35 | 46.90 | 143.04 | 219.31 | 53.33 | 8.05 | 15.07 | 87.16 | 321.13 | 541.69 | 68.68 | 0.67 | 1.93 | 189.92 | 16.89 | 49.68 | 194.14 | 0.00027 | 0.00026 | -2.03762 | 0.02787 | 0.03208 | 15.11 |
| Liberia | 1.58 | 2.37 | 50.58 | 37.58 | 61.57 | 63.82 | 10.36 | 8.98 | -13.27 | 534.30 | 237.52 | -55.55 | 0.42 | 0.78 | 83.70 | 9.53 | 16.90 | 77.35 | 0.00007 | 0.00003 | -62.21365 | 0.00844 | 0.00765 | -9.36 |
| Libya | 3.73 | 4.28 | 14.58 | 94.27 | 125.32 | 32.94 | 4.41 | 3.54 | -19.79 | 162.63 | 83.79 | -48.48 | 3.72 | 4.42 | 18.59 | 89.10 | 103.99 | 16.71 | 0.00000 | 0.00000 | -61.33280 | 0.01344 | 0.01860 | 38.37 |
| Lithuania | 3.42 | 0.69 | -79.90 | 78.68 | 16.63 | -78.86 | 0.84 | 0.48 | -43.34 | 30.03 | 15.17 | -49.50 | 3.96 | 1.60 | -59.60 | 105.84 | 39.01 | -63.14 | 0.00007 | 0.00000 | -93.72822 | 0.01163 | 0.00488 | -58.01 |
| Luxembourg | 2.87 | 1.01 | -64.85 | 65.87 | 25.97 | -60.57 | 1.44 | 0.49 | -66.07 | 24.37 | 6.81 | -72.05 | 4.85 | 1.88 | -61.32 | 118.53 | 42.17 | -64.42 | 0.00004 | 0.00000 | -89.35076 | 0.01415 | 0.00890 | -37.06 |
| Madagascar | 1.49 | 2.44 | 63.99 | 32.90 | 56.63 | 72.10 | 3.51 | 4.48 | 27.40 | 162.02 | 136.40 | -15.82 | 0.13 | 0.25 | 92.38 | 3.12 | 5.92 | 89.79 | 0.00009 | 0.00005 | -50.92871 | 0.00962 | 0.00675 | -29.80 |
| Malawi | 1.04 | 1.53 | 46.27 | 23.82 | 37.72 | 58.37 | 3.93 | 4.66 | 18.76 | 170.99 | 144.49 | -15.50 | 0.11 | 0.24 | 122.30 | 2.47 | 5.52 | 123.33 | 0.00016 | 0.00006 | -59.50164 | 0.01566 | 0.00924 | -40.99 |
| Malaysia | 8.42 | 3.23 | -61.65 | 178.01 | 73.47 | -58.73 | 7.74 | 8.36 | 8.01 | 193.64 | 150.50 | -22.28 | 3.02 | 2.42 | -19.80 | 69.12 | 53.18 | -23.06 | 0.00001 | 0.00000 | -76.69020 | 0.01223 | 0.00980 | -19.83 |
| Maldives | 7.62 | 2.79 | -63.44 | 154.53 | 61.24 | -60.37 | 1.52 | 0.71 | -53.51 | 51.46 | 15.04 | -70.78 | 0.65 | 0.69 | 6.32 | 13.60 | 13.41 | -1.36 | 0.00000 | 0.00000 | -66.54030 | 0.00246 | 0.00494 | 100.46 |
| Mali | 2.12 | 3.46 | 63.46 | 55.78 | 93.60 | 67.80 | 3.94 | 5.39 | 36.77 | 194.41 | 245.45 | 26.26 | 0.19 | 0.43 | 121.19 | 4.56 | 9.73 | 113.26 | 0.00009 | 0.00005 | -47.50985 | 0.00917 | 0.00664 | -27.66 |
| Malta | 2.32 | 0.99 | -57.42 | 51.81 | 25.46 | -50.85 | 2.07 | 1.15 | -44.76 | 35.89 | 17.03 | -52.54 | 3.10 | 1.88 | -39.12 | 71.99 | 42.14 | -41.46 | 0.00013 | 0.00003 | -77.57745 | 0.02253 | 0.01236 | -45.14 |
| Marshall Islands | 4.21 | 4.03 | -4.21 | 89.57 | 82.65 | -7.73 | 2.57 | 2.75 | 6.84 | 86.57 | 82.78 | -4.38 | 0.75 | 1.54 | 105.78 | 18.12 | 35.73 | 97.22 | 0.00000 | 0.00000 | -9.75087 | 0.00127 | 0.00277 | 119.07 |
| Mauritania | 4.41 | 4.95 | 12.19 | 109.42 | 130.95 | 19.67 | 15.58 | 17.76 | 13.99 | 545.09 | 453.56 | -16.79 | 0.92 | 2.12 | 130.57 | 21.68 | 45.46 | 109.70 | 0.00007 | 0.00005 | -31.11147 | 0.01087 | 0.01726 | 58.77 |
| Mauritius | 2.93 | 1.87 | -35.95 | 68.05 | 47.43 | -30.30 | 3.65 | 1.44 | -60.43 | 83.63 | 33.68 | -59.72 | 1.34 | 1.27 | -4.94 | 31.63 | 28.80 | -8.95 | 0.00000 | 0.00000 | 697.80805 | 0.00632 | 0.00850 | 34.48 |
| Mexico | 6.78 | 4.17 | -38.45 | 114.03 | 72.25 | -36.64 | 6.44 | 2.12 | -67.03 | 273.18 | 61.87 | -77.35 | 2.10 | 1.24 | -41.00 | 45.85 | 26.43 | -42.35 | 0.00359 | 0.00036 | -90.00389 | 0.32701 | 0.04055 | -87.60 |
| Micronesia (Federated States of) | 5.05 | 4.88 | -3.30 | 108.66 | 101.07 | -6.99 | 3.25 | 3.30 | 1.44 | 117.63 | 89.22 | -24.15 | 0.94 | 1.90 | 102.60 | 23.17 | 45.65 | 97.01 | 0.00000 | 0.00000 | -41.69894 | 0.00176 | 0.00329 | 87.00 |
| Monaco | 0.87 | 0.99 | 13.08 | 23.84 | 26.98 | 13.16 | 0.78 | 1.02 | 31.39 | 14.09 | 14.38 | 2.08 | 2.79 | 5.09 | 82.31 | 68.46 | 117.41 | 71.49 | 0.00000 | 0.00000 | -46.19552 | 0.00849 | 0.00973 | 14.61 |
| Mongolia | 3.98 | 4.17 | 4.72 | 81.14 | 85.38 | 5.22 | 9.64 | 3.12 | -67.61 | 694.68 | 161.59 | -76.74 | 3.47 | 5.94 | 71.42 | 80.30 | 129.56 | 61.35 | 0.00042 | 0.00037 | -10.63438 | 0.03911 | 0.03903 | -0.20 |
| Montenegro | 1.68 | 1.20 | -28.48 | 37.85 | 27.28 | -27.93 | 1.61 | 0.92 | -43.29 | 65.24 | 20.79 | -68.14 | 8.01 | 7.53 | -5.91 | 208.79 | 189.21 | -9.38 | 0.00005 | 0.00001 | -76.68594 | 0.01224 | 0.00763 | -37.70 |
| Morocco | 2.92 | 5.38 | 84.55 | 65.79 | 125.53 | 90.81 | 4.65 | 4.32 | -7.21 | 256.50 | 119.86 | -53.27 | 1.45 | 3.56 | 146.11 | 36.97 | 87.75 | 137.38 | 0.00001 | 0.00001 | 24.97094 | 0.00663 | 0.01934 | 191.56 |
| Mozambique | 0.60 | 1.24 | 106.89 | 13.60 | 31.14 | 128.91 | 2.30 | 3.74 | 62.56 | 112.41 | 111.53 | -0.79 | 0.07 | 0.25 | 255.38 | 1.55 | 5.67 | 266.47 | 0.00012 | 0.00006 | -46.24757 | 0.01107 | 0.00731 | -33.99 |
| Myanmar | 9.01 | 11.79 | 30.81 | 188.21 | 242.44 | 28.81 | 7.68 | 5.71 | -25.69 | 445.06 | 207.26 | -53.43 | 1.18 | 2.50 | 111.38 | 29.37 | 58.16 | 98.03 | 0.00001 | 0.00001 | -39.81864 | 0.00429 | 0.00819 | 91.18 |
| Namibia | 6.12 | 6.48 | 5.74 | 135.93 | 149.24 | 9.79 | 10.33 | 11.30 | 9.32 | 339.41 | 313.97 | -7.49 | 0.45 | 1.03 | 128.92 | 10.65 | 23.63 | 121.83 | 0.00028 | 0.00019 | -30.27698 | 0.03124 | 0.02791 | -10.67 |
| Nauru | 3.82 | 1.90 | -50.29 | 84.03 | 42.68 | -49.21 | 2.51 | 1.42 | -43.28 | 89.85 | 48.10 | -46.47 | 1.26 | 1.08 | -13.99 | 30.33 | 25.34 | -16.46 | 0.00000 | 0.00000 | 13.95016 | 0.00173 | 0.00362 | 109.00 |
| Nepal | 18.02 | 47.11 | 161.48 | 352.71 | 851.90 | 141.53 | 6.76 | 8.59 | 27.12 | 429.05 | 317.26 | -26.05 | 0.38 | 1.40 | 269.64 | 9.11 | 31.48 | 245.71 | 0.00018 | 0.00009 | -50.57515 | 0.01902 | 0.01942 | 2.10 |
| Netherlands | 4.15 | 1.99 | -52.15 | 93.96 | 48.00 | -48.91 | 2.02 | 0.90 | -55.40 | 27.34 | 11.20 | -59.05 | 6.57 | 3.15 | -52.03 | 153.23 | 68.28 | -55.44 | 0.00011 | 0.00003 | -74.69634 | 0.02134 | 0.01201 | -43.72 |
| New Zealand | 1.00 | 0.50 | -49.62 | 23.87 | 11.99 | -49.76 | 0.66 | 0.15 | -77.95 | 11.11 | 2.23 | -79.90 | 1.20 | 0.59 | -50.57 | 27.85 | 12.86 | -53.82 | 0.00008 | 0.00001 | -92.35433 | 0.01477 | 0.00848 | -42.59 |
| Nicaragua | 1.29 | 4.00 | 210.22 | 23.13 | 62.36 | 169.56 | 1.93 | 2.10 | 8.87 | 112.96 | 69.01 | -38.90 | 0.29 | 0.81 | 175.78 | 6.50 | 16.58 | 154.92 | 0.00000 | 0.00000 | -54.18966 | 0.00222 | 0.00521 | 135.10 |
| Niger | 2.14 | 2.76 | 28.71 | 49.77 | 64.59 | 29.78 | 9.35 | 8.16 | -12.74 | 515.08 | 301.94 | -41.38 | 0.27 | 0.48 | 73.95 | 6.36 | 10.27 | 61.53 | 0.00008 | 0.00006 | -33.11431 | 0.00892 | 0.00736 | -17.52 |
| Nigeria | 2.73 | 4.97 | 81.79 | 66.09 | 114.58 | 73.37 | 10.86 | 16.13 | 48.53 | 569.88 | 653.43 | 14.66 | 0.44 | 1.35 | 207.12 | 9.62 | 27.70 | 187.76 | 0.00007 | 0.00006 | -15.05871 | 0.00958 | 0.01415 | 47.71 |
| Niue | 2.51 | 1.42 | -43.41 | 57.73 | 34.53 | -40.19 | 1.72 | 1.09 | -37.03 | 56.33 | 34.68 | -38.42 | 0.94 | 1.11 | 17.66 | 22.50 | 25.17 | 11.90 | 0.00000 | 0.00000 | 17.72828 | 0.00221 | 0.00452 | 104.14 |
| North Macedonia | 7.74 | 4.33 | -44.12 | 152.59 | 87.89 | -42.40 | 4.02 | 1.29 | -68.01 | 231.12 | 33.48 | -85.51 | 5.56 | 7.17 | 28.83 | 151.02 | 183.83 | 21.73 | 0.00000 | 0.00000 | -35.59484 | 0.00738 | 0.00779 | 5.52 |
| Northern Mariana Islands | 4.04 | 1.87 | -53.68 | 80.65 | 36.25 | -55.06 | 2.17 | 1.10 | -49.26 | 56.47 | 28.49 | -49.56 | 3.73 | 2.37 | -36.43 | 85.36 | 52.04 | -39.04 | 0.00000 | 0.00000 | 88.29278 | 0.00406 | 0.00443 | 9.19 |
| Norway | 1.00 | 0.57 | -42.97 | 27.79 | 13.59 | -51.10 | 2.03 | 0.29 | -85.66 | 26.07 | 3.32 | -87.28 | 1.91 | 0.69 | -63.67 | 45.49 | 14.70 | -67.68 | 0.00002 | 0.00000 | -85.35409 | 0.01119 | 0.00806 | -27.93 |
| Oman | 6.86 | 6.26 | -8.80 | 132.76 | 120.30 | -9.38 | 8.90 | 8.75 | -1.65 | 226.87 | 148.35 | -34.61 | 1.44 | 2.58 | 78.57 | 33.49 | 52.04 | 55.40 | 0.00000 | 0.00000 | -35.49035 | 0.01045 | 0.02184 | 109.02 |
| Pakistan | 10.91 | 19.54 | 79.06 | 221.71 | 408.18 | 84.11 | 4.43 | 7.27 | 64.16 | 251.52 | 345.87 | 37.51 | 0.93 | 3.04 | 226.27 | 22.82 | 75.37 | 230.26 | 0.00026 | 0.00014 | -45.62141 | 0.02671 | 0.02215 | -17.06 |
| Palestine | 3.94 | 3.39 | -13.83 | 89.22 | 83.17 | -6.78 | 4.04 | 4.12 | 2.07 | 114.23 | 81.67 | -28.50 | 2.40 | 4.77 | 98.28 | 55.26 | 108.68 | 96.67 | 0.00000 | 0.00000 | -58.50125 | 0.00722 | 0.01576 | 118.25 |
| Panama | 1.82 | 1.43 | -21.16 | 34.52 | 25.67 | -25.65 | 1.60 | 1.26 | -21.43 | 53.48 | 41.62 | -22.19 | 1.19 | 0.97 | -19.00 | 28.19 | 20.95 | -25.70 | 0.00011 | 0.00049 | 334.63004 | 0.01474 | 0.05023 | 240.76 |
| Papua New Guinea | 6.01 | 7.52 | 25.16 | 119.83 | 147.45 | 23.05 | 2.85 | 2.94 | 3.19 | 159.62 | 145.94 | -8.57 | 0.44 | 0.80 | 80.18 | 10.46 | 18.55 | 77.24 | 0.00001 | 0.00001 | -16.97253 | 0.00185 | 0.00236 | 27.55 |
| Paraguay | 1.23 | 1.54 | 25.84 | 23.06 | 29.90 | 29.66 | 1.85 | 1.69 | -8.76 | 70.31 | 40.79 | -41.98 | 0.60 | 1.24 | 106.11 | 14.15 | 27.73 | 96.00 | 0.00000 | 0.00000 | -48.00965 | 0.00559 | 0.00872 | 55.96 |
| Peru | 2.12 | 1.73 | -18.45 | 41.52 | 34.62 | -16.62 | 15.12 | 8.25 | -45.43 | 637.73 | 181.69 | -71.51 | 1.94 | 1.87 | -3.41 | 45.39 | 40.55 | -10.67 | 0.00043 | 0.00008 | -82.17156 | 0.04708 | 0.02087 | -55.67 |
| Philippines | 6.48 | 3.75 | -42.19 | 134.17 | 98.26 | -26.76 | 8.49 | 7.69 | -9.46 | 314.52 | 176.71 | -43.82 | 2.78 | 1.97 | -29.28 | 68.82 | 47.29 | -31.29 | 0.00001 | 0.00001 | -33.22054 | 0.00936 | 0.00743 | -20.63 |
| Poland | 5.11 | 2.09 | -59.07 | 124.04 | 57.67 | -53.50 | 2.83 | 1.96 | -30.90 | 86.16 | 40.88 | -52.55 | 8.02 | 6.74 | -15.94 | 214.12 | 159.79 | -25.37 | 0.00016 | 0.00000 | -97.60665 | 0.02183 | 0.00758 | -65.26 |
| Portugal | 2.05 | 0.85 | -58.61 | 44.77 | 17.44 | -61.05 | 1.55 | 0.83 | -46.58 | 34.65 | 11.79 | -65.96 | 1.51 | 0.96 | -36.24 | 37.39 | 23.45 | -37.29 | 0.00002 | 0.00000 | -91.38000 | 0.00914 | 0.00704 | -22.95 |
| Qatar | 10.42 | 7.77 | -25.46 | 225.80 | 153.75 | -31.91 | 8.59 | 7.73 | -10.04 | 184.46 | 111.49 | -39.56 | 5.86 | 6.36 | 8.45 | 125.65 | 114.37 | -8.97 | 0.00001 | 0.00000 | -79.17029 | 0.03026 | 0.03103 | 2.55 |
| Republic of Korea | 4.03 | 2.46 | -38.97 | 78.37 | 56.99 | -27.28 | 2.92 | 2.81 | -3.92 | 63.88 | 35.71 | -44.10 | 3.91 | 4.88 | 24.79 | 97.09 | 92.35 | -4.89 | 0.00006 | 0.00001 | -88.16009 | 0.01787 | 0.01657 | -7.31 |
| Republic of Moldova | 5.61 | 1.44 | -74.42 | 128.34 | 33.95 | -73.55 | 3.10 | 1.35 | -56.54 | 185.16 | 62.32 | -66.34 | 3.32 | 1.67 | -49.60 | 95.72 | 45.08 | -52.91 | 0.00016 | 0.00020 | 22.42147 | 0.01897 | 0.02274 | 19.90 |
| Romania | 7.01 | 1.97 | -71.96 | 139.24 | 50.07 | -64.04 | 5.10 | 1.71 | -66.40 | 302.43 | 67.59 | -77.65 | 3.15 | 3.43 | 8.74 | 93.83 | 90.69 | -3.35 | 0.00006 | 0.00001 | -87.23807 | 0.01028 | 0.00566 | -44.98 |
| Russian Federation | 3.59 | 1.11 | -69.02 | 81.97 | 27.22 | -66.79 | 1.57 | 0.87 | -44.85 | 81.04 | 32.05 | -60.45 | 3.97 | 1.74 | -56.15 | 108.84 | 44.06 | -59.52 | 0.00288 | 0.00022 | -92.40187 | 0.26219 | 0.02453 | -90.64 |
| Rwanda | 3.90 | 4.02 | 3.07 | 88.04 | 93.09 | 5.74 | 8.50 | 7.88 | -7.30 | 384.32 | 230.39 | -40.05 | 0.34 | 0.64 | 91.12 | 8.08 | 14.57 | 80.35 | 0.00014 | 0.00014 | -0.38379 | 0.01427 | 0.01903 | 33.38 |
| Saint Kitts and Nevis | 0.89 | 0.74 | -17.30 | 18.24 | 16.49 | -9.63 | 2.69 | 1.46 | -45.91 | 60.98 | 31.52 | -48.31 | 0.66 | 0.59 | -9.89 | 15.68 | 13.54 | -13.64 | 0.00003 | 0.00002 | -22.45725 | 0.00610 | 0.00788 | 29.19 |
| Saint Lucia | 3.00 | 3.09 | 2.99 | 57.95 | 64.36 | 11.07 | 4.06 | 3.08 | -24.01 | 89.52 | 65.50 | -26.83 | 1.38 | 1.77 | 28.20 | 32.69 | 41.30 | 26.33 | 0.00003 | 0.00002 | -43.72660 | 0.00900 | 0.01209 | 34.40 |
| Saint Vincent and the Grenadines | 1.14 | 1.59 | 39.64 | 22.13 | 32.93 | 48.80 | 3.79 | 3.65 | -3.60 | 97.70 | 78.71 | -19.44 | 0.92 | 1.48 | 59.83 | 21.78 | 34.60 | 58.85 | 0.00004 | 0.00004 | 9.17889 | 0.00907 | 0.01406 | 54.93 |
| Samoa | 5.00 | 3.63 | -27.50 | 100.55 | 70.85 | -29.54 | 2.94 | 2.30 | -21.48 | 98.54 | 58.01 | -41.13 | 0.51 | 0.61 | 20.75 | 12.43 | 14.76 | 18.80 | 0.00000 | 0.00000 | -54.78697 | 0.00274 | 0.00311 | 13.50 |
| San Marino | 1.04 | 0.64 | -38.95 | 29.67 | 19.24 | -35.17 | 0.70 | 0.37 | -46.40 | 11.48 | 5.42 | -52.83 | 2.87 | 2.03 | -29.06 | 65.75 | 45.65 | -30.57 | 0.00008 | 0.00004 | -48.13393 | 0.01591 | 0.01267 | -20.39 |
| Sao Tome and Principe | 4.13 | 8.68 | 110.26 | 91.62 | 205.16 | 123.92 | 7.95 | 13.08 | 64.47 | 307.62 | 307.17 | -0.15 | 0.62 | 2.26 | 266.69 | 13.76 | 49.81 | 261.96 | 0.00003 | 0.00003 | -20.92332 | 0.00509 | 0.00818 | 60.74 |
| Saudi Arabia | 7.21 | 6.64 | -7.86 | 144.10 | 168.68 | 17.05 | 7.16 | 8.72 | 21.83 | 175.70 | 179.22 | 2.00 | 1.35 | 2.59 | 91.09 | 31.26 | 58.69 | 87.74 | 0.00000 | 0.00000 | -73.09820 | 0.01091 | 0.02780 | 154.88 |
| Senegal | 3.69 | 4.42 | 19.68 | 83.67 | 108.29 | 29.43 | 9.04 | 10.48 | 16.02 | 374.43 | 304.04 | -18.80 | 0.64 | 1.37 | 112.58 | 14.81 | 30.11 | 103.23 | 0.00009 | 0.00006 | -30.40233 | 0.01126 | 0.01166 | 3.47 |
| Serbia | 5.27 | 3.76 | -28.78 | 118.96 | 89.07 | -25.12 | 2.39 | 1.48 | -38.22 | 103.44 | 29.28 | -71.70 | 6.31 | 7.56 | 19.80 | 171.16 | 194.38 | 13.56 | 0.00000 | 0.00000 | -56.54396 | 0.00739 | 0.00749 | 1.31 |
| Seychelles | 3.28 | 2.60 | -20.63 | 74.80 | 63.88 | -14.59 | 6.42 | 6.39 | -0.42 | 148.34 | 135.23 | -8.84 | 1.96 | 2.03 | 3.71 | 46.52 | 45.73 | -1.70 | 0.00001 | 0.00000 | -49.99960 | 0.00763 | 0.00965 | 26.46 |
| Sierra Leone | 2.51 | 3.17 | 26.17 | 56.78 | 79.65 | 40.27 | 10.76 | 12.11 | 12.53 | 539.38 | 443.18 | -17.83 | 0.40 | 0.77 | 94.01 | 9.05 | 17.22 | 90.25 | 0.00008 | 0.00004 | -53.93771 | 0.00850 | 0.00629 | -26.08 |
| Singapore | 6.87 | 1.15 | -83.30 | 136.20 | 31.01 | -77.23 | 8.75 | 4.79 | -45.23 | 149.19 | 66.97 | -55.11 | 5.74 | 2.79 | -51.43 | 125.17 | 54.12 | -56.76 | 0.00000 | 0.00000 | -9.99464 | 0.01252 | 0.01278 | 2.07 |
| Slovakia | 2.76 | 1.38 | -49.98 | 65.91 | 37.34 | -43.34 | 7.07 | 2.04 | -71.22 | 182.79 | 42.98 | -76.49 | 6.99 | 3.57 | -48.97 | 187.25 | 85.65 | -54.26 | 0.00001 | 0.00000 | -67.87594 | 0.00769 | 0.00586 | -23.81 |
| Slovenia | 4.37 | 1.30 | -70.31 | 94.59 | 31.41 | -66.79 | 3.19 | 1.12 | -64.76 | 59.10 | 15.44 | -73.88 | 5.74 | 3.63 | -36.77 | 149.54 | 85.52 | -42.81 | 0.00019 | 0.00001 | -97.14697 | 0.02386 | 0.00634 | -73.44 |
| Solomon Islands | 2.51 | 4.55 | 81.02 | 54.53 | 93.99 | 72.35 | 4.23 | 5.36 | 26.61 | 131.21 | 148.82 | 13.42 | 0.39 | 1.01 | 156.81 | 9.97 | 25.19 | 152.56 | 0.00000 | 0.00000 | -5.81935 | 0.00088 | 0.00188 | 113.42 |
| Somalia | 0.99 | 1.01 | 1.79 | 22.16 | 23.55 | 6.25 | 2.57 | 2.50 | -3.04 | 123.21 | 95.35 | -22.61 | 0.07 | 0.08 | 11.70 | 1.78 | 1.99 | 12.09 | 0.00014 | 0.00009 | -32.49279 | 0.01307 | 0.00933 | -28.61 |
| South Africa | 6.05 | 5.66 | -6.46 | 156.22 | 145.70 | -6.73 | 11.71 | 9.87 | -15.69 | 524.83 | 304.10 | -42.06 | 3.22 | 3.28 | 2.04 | 82.97 | 77.47 | -6.63 | 0.00048 | 0.00028 | -40.32701 | 0.05327 | 0.04050 | -23.98 |
| South Sudan | 2.40 | 2.81 | 17.12 | 54.14 | 64.67 | 19.44 | 8.08 | 8.65 | 7.07 | 414.70 | 343.44 | -17.18 | 0.39 | 0.62 | 59.12 | 9.09 | 13.64 | 50.06 | 0.00027 | 0.00019 | -30.62636 | 0.02677 | 0.02136 | -20.22 |
| Spain | 3.15 | 1.35 | -57.21 | 61.86 | 26.23 | -57.60 | 1.25 | 0.44 | -64.56 | 23.11 | 6.41 | -72.26 | 2.89 | 1.64 | -43.44 | 72.17 | 38.73 | -46.33 | 0.00001 | 0.00000 | -76.60283 | 0.00911 | 0.00786 | -13.77 |
| Sri Lanka | 3.15 | 2.97 | -5.86 | 72.28 | 74.14 | 2.57 | 4.80 | 2.21 | -53.90 | 109.68 | 48.18 | -56.07 | 0.62 | 1.14 | 84.24 | 15.19 | 26.79 | 76.33 | 0.00002 | 0.00000 | -83.44724 | 0.00742 | 0.00896 | 20.71 |
| Sudan | 3.03 | 7.16 | 135.89 | 66.23 | 164.04 | 147.68 | 4.14 | 5.90 | 42.31 | 228.46 | 189.31 | -17.14 | 0.38 | 1.63 | 333.93 | 8.88 | 37.42 | 321.59 | 0.00001 | 0.00001 | 21.09871 | 0.00331 | 0.01436 | 333.76 |
| Suriname | 2.68 | 2.15 | -19.82 | 54.82 | 46.56 | -15.07 | 4.60 | 3.28 | -28.73 | 149.22 | 86.95 | -41.73 | 1.66 | 2.11 | 26.96 | 39.96 | 50.16 | 25.55 | 0.00002 | 0.00001 | -66.52275 | 0.01017 | 0.01043 | 2.51 |
| Sweden | 0.60 | 0.26 | -57.18 | 19.22 | 7.04 | -63.35 | 1.02 | 0.13 | -87.32 | 15.26 | 1.80 | -88.22 | 1.23 | 0.39 | -68.20 | 29.04 | 8.11 | -72.07 | 0.00000 | 0.00000 | 426.77644 | 0.00792 | 0.00847 | 6.94 |
| Switzerland | 2.20 | 0.71 | -67.60 | 55.44 | 18.74 | -66.20 | 2.09 | 0.34 | -83.53 | 33.08 | 4.63 | -86.00 | 4.66 | 1.40 | -70.06 | 113.95 | 30.99 | -72.81 | 0.00000 | 0.00000 | 59.24311 | 0.01130 | 0.00864 | -23.56 |
| Syrian Arab Republic | 4.40 | 4.26 | -3.15 | 108.10 | 106.85 | -1.16 | 4.42 | 3.79 | -14.25 | 213.58 | 104.90 | -50.89 | 1.93 | 2.33 | 20.32 | 47.46 | 54.34 | 14.50 | 0.00002 | 0.00000 | -87.01278 | 0.01544 | 0.01548 | 0.25 |
| Taiwan (Province of China) | 4.19 | 3.31 | -21.01 | 82.85 | 56.85 | -31.37 | 3.70 | 3.89 | 5.22 | 72.00 | 60.47 | -16.02 | 3.38 | 4.89 | 44.50 | 79.40 | 102.69 | 29.32 | 0.00037 | 0.00003 | -91.59069 | 0.03982 | 0.01201 | -69.84 |
| Tajikistan | 4.76 | 6.71 | 41.17 | 115.45 | 141.72 | 22.75 | 10.17 | 7.40 | -27.19 | 728.20 | 351.20 | -51.77 | 1.69 | 2.11 | 25.40 | 47.82 | 50.03 | 4.62 | 0.00097 | 0.00086 | -11.41398 | 0.08749 | 0.07961 | -9.01 |
| Thailand | 9.38 | 3.53 | -62.41 | 211.20 | 90.58 | -57.11 | 4.14 | 4.57 | 10.52 | 158.06 | 99.78 | -36.88 | 3.81 | 3.84 | 0.84 | 94.61 | 85.38 | -9.76 | 0.00002 | 0.00000 | -79.87529 | 0.01503 | 0.01823 | 21.27 |
| Timor-Leste | 2.01 | 4.11 | 104.32 | 40.96 | 83.70 | 104.36 | 3.24 | 4.29 | 32.37 | 195.56 | 120.14 | -38.56 | 0.33 | 1.14 | 247.04 | 7.78 | 25.89 | 232.94 | 0.00001 | 0.00000 | -59.66730 | 0.00178 | 0.00393 | 120.98 |
| Togo | 3.06 | 3.73 | 21.86 | 72.82 | 98.93 | 35.84 | 9.70 | 12.98 | 33.81 | 352.09 | 359.11 | 1.99 | 0.53 | 1.09 | 107.19 | 12.20 | 25.07 | 105.54 | 0.00005 | 0.00002 | -53.50074 | 0.00732 | 0.00846 | 15.65 |
| Tokelau | 2.81 | 1.20 | -57.33 | 62.49 | 28.68 | -54.10 | 1.72 | 0.77 | -54.98 | 60.09 | 19.80 | -67.04 | 0.83 | 0.77 | -7.32 | 19.14 | 17.35 | -9.36 | 0.00000 | 0.00000 | -70.38910 | 0.00491 | 0.00561 | 14.37 |
| Tonga | 3.02 | 2.69 | -10.98 | 58.47 | 52.29 | -10.57 | 2.28 | 2.21 | -2.70 | 64.25 | 54.25 | -15.57 | 1.19 | 1.85 | 54.92 | 26.54 | 41.34 | 55.73 | 0.00000 | 0.00000 | -40.72848 | 0.00250 | 0.00389 | 55.81 |
| Trinidad and Tobago | 2.09 | 1.50 | -28.29 | 42.53 | 33.31 | -21.70 | 4.77 | 1.88 | -60.64 | 122.09 | 50.23 | -58.85 | 1.68 | 1.52 | -9.76 | 40.99 | 37.07 | -9.57 | 0.00000 | 0.00000 | 26.65968 | 0.00970 | 0.01002 | 3.33 |
| Tunisia | 3.46 | 3.38 | -2.30 | 79.18 | 89.10 | 12.52 | 4.75 | 2.72 | -42.71 | 221.22 | 60.49 | -72.66 | 3.36 | 3.99 | 18.78 | 77.17 | 93.30 | 20.89 | 0.00001 | 0.00000 | -39.38244 | 0.01103 | 0.01643 | 48.92 |
| Turkey | 7.99 | 6.34 | -20.73 | 178.49 | 149.16 | -16.43 | 6.55 | 2.56 | -60.87 | 415.83 | 56.22 | -86.48 | 5.95 | 6.00 | 0.91 | 152.62 | 144.41 | -5.38 | 0.00006 | 0.00001 | -87.49267 | 0.01784 | 0.01802 | 1.02 |
| Turkmenistan | 5.98 | 1.95 | -67.36 | 132.16 | 49.23 | -62.75 | 11.36 | 3.51 | -69.13 | 886.49 | 239.77 | -72.95 | 2.60 | 1.67 | -35.73 | 75.31 | 44.63 | -40.74 | 0.00115 | 0.00071 | -38.41297 | 0.10556 | 0.06850 | -35.11 |
| Tuvalu | 2.97 | 2.03 | -31.74 | 67.51 | 45.84 | -32.09 | 2.38 | 1.34 | -43.51 | 113.21 | 37.40 | -66.96 | 0.46 | 0.84 | 81.51 | 11.31 | 19.50 | 72.46 | 0.00000 | 0.00000 | -43.81705 | 0.00102 | 0.00298 | 193.83 |
| Uganda | 1.90 | 3.19 | 67.74 | 40.70 | 74.23 | 82.37 | 3.38 | 7.30 | 115.93 | 126.32 | 196.39 | 55.48 | 0.15 | 0.50 | 247.69 | 3.42 | 11.73 | 243.35 | 0.00010 | 0.00008 | -22.02532 | 0.01094 | 0.01390 | 27.12 |
| Ukraine | 6.80 | 1.41 | -79.20 | 154.22 | 37.15 | -75.91 | 1.32 | 1.06 | -19.80 | 56.31 | 42.18 | -25.10 | 5.61 | 2.29 | -59.11 | 158.70 | 63.31 | -60.11 | 0.00031 | 0.00007 | -77.14280 | 0.03555 | 0.01216 | -65.81 |
| United Arab Emirates | 11.21 | 8.36 | -25.47 | 261.15 | 232.41 | -11.00 | 17.65 | 10.71 | -39.30 | 278.22 | 153.89 | -44.69 | 5.21 | 5.03 | -3.48 | 105.28 | 100.30 | -4.73 | 0.00000 | 0.00000 | -50.35065 | 0.01932 | 0.02181 | 12.87 |
| United Kingdom | 3.94 | 1.69 | -57.20 | 89.74 | 37.95 | -57.71 | 3.63 | 1.14 | -68.50 | 51.85 | 15.51 | -70.08 | 5.54 | 2.01 | -63.81 | 124.57 | 40.67 | -67.35 | 0.00000 | 0.00000 | -54.54077 | 0.01203 | 0.00966 | -19.73 |
| United Republic of Tanzania | 1.03 | 1.74 | 68.60 | 23.56 | 42.13 | 78.80 | 4.94 | 6.01 | 21.68 | 223.38 | 194.40 | -12.97 | 0.19 | 0.52 | 168.87 | 4.53 | 11.94 | 163.66 | 0.00012 | 0.00008 | -28.28398 | 0.01231 | 0.01248 | 1.41 |
| United States of America | 2.26 | 1.15 | -49.01 | 70.29 | 31.43 | -55.29 | 1.38 | 0.35 | -74.81 | 26.42 | 6.56 | -75.18 | 4.50 | 1.44 | -67.96 | 109.42 | 30.65 | -71.99 | 0.00006 | 0.00002 | -71.81198 | 0.01875 | 0.01112 | -40.69 |
| United States Virgin Islands | 0.69 | 0.57 | -17.91 | 13.98 | 12.29 | -12.07 | 0.84 | 0.59 | -30.17 | 20.12 | 11.03 | -45.18 | 1.09 | 1.30 | 19.45 | 25.47 | 29.78 | 16.90 | 0.00000 | 0.00000 | -61.19700 | 0.00639 | 0.00661 | 3.48 |
| Uruguay | 1.67 | 1.67 | -0.31 | 37.29 | 36.25 | -2.77 | 1.16 | 1.00 | -13.70 | 31.76 | 19.01 | -40.16 | 2.82 | 1.98 | -29.82 | 73.07 | 48.02 | -34.28 | 0.00000 | 0.00000 | -77.75659 | 0.00677 | 0.00779 | 15.11 |
| Uzbekistan | 5.41 | 4.08 | -24.70 | 126.38 | 85.67 | -32.22 | 8.63 | 6.79 | -21.27 | 659.54 | 389.90 | -40.88 | 2.47 | 2.53 | 2.15 | 69.72 | 62.23 | -10.74 | 0.00750 | 0.00653 | -12.88405 | 0.66846 | 0.58559 | -12.40 |
| Vanuatu | 4.46 | 4.73 | 6.08 | 90.24 | 95.76 | 6.12 | 2.31 | 2.86 | 24.04 | 77.90 | 89.90 | 15.41 | 0.57 | 1.14 | 99.11 | 13.24 | 26.38 | 99.29 | 0.00000 | 0.00000 | -27.43117 | 0.00157 | 0.00273 | 74.27 |
| Venezuela (Bolivarian Republic of) | 2.51 | 3.41 | 35.77 | 49.99 | 67.71 | 35.45 | 3.38 | 2.32 | -31.33 | 112.46 | 71.44 | -36.48 | 2.53 | 2.96 | 16.91 | 62.37 | 69.02 | 10.66 | 0.00005 | 0.00001 | -86.57501 | 0.01337 | 0.01104 | -17.45 |
| Viet Nam | 4.19 | 4.80 | 14.70 | 81.55 | 103.30 | 26.68 | 2.93 | 2.80 | -4.42 | 127.41 | 72.47 | -43.12 | 1.16 | 3.06 | 163.84 | 29.42 | 75.96 | 158.22 | 0.00001 | 0.00000 | -37.57545 | 0.00314 | 0.00671 | 113.45 |
| Yemen | 3.08 | 6.89 | 123.82 | 68.70 | 159.58 | 132.28 | 3.56 | 5.88 | 65.15 | 192.05 | 191.34 | -0.37 | 0.42 | 1.66 | 294.00 | 10.40 | 38.73 | 272.21 | 0.00001 | 0.00001 | 81.23778 | 0.00418 | 0.01645 | 293.76 |
| Zambia | 2.13 | 3.32 | 55.84 | 47.76 | 80.17 | 67.86 | 7.77 | 8.44 | 8.59 | 358.30 | 246.46 | -31.21 | 0.36 | 0.96 | 164.70 | 8.45 | 22.69 | 168.54 | 0.00013 | 0.00009 | -31.68532 | 0.01506 | 0.01574 | 4.47 |
| Zimbabwe | 2.15 | 2.20 | 2.33 | 54.69 | 59.23 | 8.30 | 8.96 | 11.31 | 26.16 | 290.04 | 358.49 | 23.60 | 0.73 | 0.96 | 31.93 | 17.76 | 23.88 | 34.43 | 0.00034 | 0.00024 | -29.78208 | 0.03443 | 0.02648 | -23.08 |
| Puerto Rico | 0.77 | 0.49 | -36.24 | 14.78 | 10.13 | -31.47 | 1.04 | 0.33 | -68.60 | 24.08 | 7.68 | -68.12 | 0.68 | 0.36 | -46.76 | 15.26 | 7.90 | -48.25 | 0.00000 | 0.00000 |  | 0.00589 | 0.00589 |  |
| Iceland | 0.64 | 0.27 | -58.09 | 16.50 | 7.20 | -56.36 | 0.77 | 0.18 | -76.78 | 11.56 | 2.60 | -77.52 | 1.16 | 0.52 | -54.91 | 27.00 | 11.56 | -57.17 | 0.00001 | 0.00000 |  | 0.00767 | 0.00725 |  |
| Australia | 1.11 | 0.54 | -51.35 | 26.55 | 13.35 | -49.73 | 0.29 | 0.17 | -41.69 | 5.63 | 2.56 | -54.58 | 1.35 | 0.72 | -46.36 | 31.21 | 15.13 | -51.54 | 0.00002 | 0.00000 |  | 0.01013 | 0.00817 |  |
| Palau | 2.88 | 1.98 | -31.11 | 59.23 | 41.20 | -30.45 | 3.63 | 2.49 | -31.53 | 127.46 | 72.48 | -43.13 | 1.45 | 1.45 | -0.19 | 34.28 | 33.68 | -1.73 | 0.00000 | 0.00000 |  | 0.00510 | 0.00576 |  |

Abbreviations: DALY disability-adjusted life year; COPD chronic obstructive pulmonary disease; TBL cancer tracheal, bronchus, and lung cancer; LRIs lower respiratory infections; URIs upper respiratory infections.

**Table S6 Relative burden of respiratory diseases attributable to ambient particulate matter pollution among 204 countries in 2019.**

| **Nations** | **COPD** | | **TBL cancer** | | **LRIs** | | **URIs** | |
| --- | --- | --- | --- | --- | --- | --- | --- | --- |
|  | Deaths (%), 2019 (95% UI) | DALY (%), 2019 (95% UI) | Deaths (%), 2019 (95% UI) | DALY (%), 2019 (95% UI) | Deaths (%), 2019 (95% UI) | DALY (%), 2019 (95% UI) | Deaths (%), 2019 (95% UI) | DALY (%), 2019 (95% UI) |
| Afghanistan | 13.92(7.57 to 22.35) | 12.59(6.77 to 20.33) | 8.67(3.67 to 15.41) | 8.5(3.62 to 15.14) | 10.78(4.87 to 18.86) | 10.68(4.77 to 18.95) | 0.09(0.02 to 0.26) | 0.02(0.01 to 0.03) |
| Albania | 12.4(9 to 16.3) | 12.19(8.84 to 16.07) | 12.06(8.51 to 15.84) | 11.99(8.45 to 15.76) | 9.47(5.87 to 14.13) | 9.49(5.95 to 14.14) | 0.06(0 to 0.26) | 0.03(0 to 0.12) |
| Algeria | 21.31(14.11 to 30.24) | 20.45(13.53 to 29.01) | 20.62(14.03 to 27.76) | 20.55(13.99 to 27.69) | 16.63(9.3 to 25.55) | 16.74(9.61 to 25.66) | 0.07(0.02 to 0.2) | 0.02(0.01 to 0.04) |
| American Samoa | 3.54(1.23 to 7.16) | 3.42(1.2 to 6.9) | 3.61(1.35 to 7.66) | 3.57(1.34 to 7.57) | 2.68(0.93 to 6.09) | 2.72(1.01 to 6.14) | 0.02(0 to 0.06) | 0(0 to 0.01) |
| Andorra | 4.87(2.48 to 7.83) | 4.85(2.47 to 7.8) | 5.46(2.84 to 8.69) | 5.46(2.84 to 8.68) | 3.53(1.66 to 6.25) | 3.53(1.67 to 6.26) | 0(0 to 0) | 0(0 to 0.01) |
| Angola | 13.94(8.1 to 21.8) | 12.78(7.44 to 20) | 10.68(4.96 to 17.25) | 10.62(4.93 to 17.14) | 11.03(5.85 to 17.97) | 10.91(5.81 to 17.83) | 0.07(0.02 to 0.22) | 0.03(0.01 to 0.07) |
| Antigua and Barbuda | 11.68(4.01 to 21.82) | 11.39(3.88 to 21.35) | 12.25(4.5 to 21.76) | 12.22(4.49 to 21.71) | 8.75(2.79 to 18.44) | 8.81(2.95 to 18.44) | 0.02(0 to 0.05) | 0.01(0 to 0.02) |
| Argentina | 8.74(4.99 to 13.43) | 8.61(4.91 to 13.24) | 9.25(5.47 to 13.83) | 9.23(5.46 to 13.78) | 6.47(3.4 to 11.06) | 6.48(3.4 to 11.07) | 0.02(0 to 0.07) | 0.01(0 to 0.02) |
| Armenia | 21.7(13.76 to 30.87) | 21.53(13.65 to 30.64) | 20.35(13.49 to 27.06) | 20.3(13.46 to 26.99) | 16.82(9.36 to 26.92) | 16.9(9.49 to 26.93) | 0.31(0.1 to 0.56) | 0.02(0.01 to 0.03) |
| Australia | 2.73(0.68 to 5.66) | 2.71(0.68 to 5.64) | 3.08(0.74 to 6) | 3.08(0.74 to 6) | 1.95(0.47 to 4.29) | 1.97(0.49 to 4.29) | 0.01(0 to 0.05) | 0.01(0 to 0.02) |
| Austria | 7.62(4.84 to 11.09) | 7.58(4.81 to 11.03) | 8.36(5.51 to 12) | 8.35(5.51 to 12) | 5.59(3.13 to 9.1) | 5.6(3.17 to 9.1) | 0.02(0 to 0.08) | 0.01(0 to 0.02) |
| Azerbaijan | 16.76(9.25 to 25.9) | 16.34(9.01 to 25.28) | 16.37(9.53 to 23.74) | 16.28(9.47 to 23.59) | 13.12(6.87 to 22.49) | 13.22(7.06 to 22.48) | 1.19(0.1 to 4.3) | 0.3(0.01 to 1.3) |
| Bahamas | 10.14(2.72 to 20.7) | 9.86(2.66 to 20.11) | 10.74(2.99 to 20.86) | 10.7(2.98 to 20.77) | 7.6(1.79 to 16.45) | 7.65(1.98 to 16.46) | 0.01(0 to 0.03) | 0.01(0 to 0.01) |
| Bahrain | 33(25.04 to 41.75) | 32.12(24.41 to 40.64) | 28.31(21.29 to 35.33) | 28.26(21.25 to 35.27) | 26.5(17.86 to 36.44) | 26.51(17.87 to 36.44) | 0.03(0.01 to 0.05) | 0.02(0.01 to 0.02) |
| Bangladesh | 23.17(17.16 to 29.91) | 22.55(16.73 to 29.14) | 15.5(9.48 to 21.63) | 15.38(9.39 to 21.51) | 18.41(12.66 to 24.47) | 18.03(12.11 to 24.02) | 0.08(0.02 to 0.18) | 0.02(0.01 to 0.03) |
| Barbados | 14.31(5.67 to 25.31) | 14.1(5.58 to 24.98) | 14.71(6.13 to 24.78) | 14.68(6.12 to 24.73) | 10.8(3.78 to 21.41) | 10.84(3.83 to 21.42) | 0.01(0 to 0.03) | 0.01(0 to 0.02) |
| Belarus | 11.05(7.37 to 15.7) | 10.96(7.32 to 15.59) | 11.68(7.93 to 16.14) | 11.68(7.93 to 16.13) | 8.22(4.76 to 12.97) | 8.25(4.78 to 13) | 0.07(0.01 to 0.17) | 0.01(0 to 0.02) |
| Belgium | 8.05(5.19 to 11.55) | 8.02(5.17 to 11.5) | 8.82(5.88 to 12.31) | 8.81(5.87 to 12.3) | 5.91(3.39 to 9.68) | 5.92(3.4 to 9.68) | 0.01(0 to 0.02) | 0.01(0 to 0.02) |
| Belize | 13.6(4.96 to 26.03) | 13.05(4.76 to 25.01) | 12.91(4.38 to 23.71) | 12.81(4.34 to 23.53) | 10.51(3.48 to 20.71) | 10.54(3.66 to 20.66) | 0.04(0 to 0.1) | 0.01(0 to 0.02) |
| Benin | 12.31(6.35 to 19.83) | 11.07(5.74 to 17.78) | 7.56(3.14 to 13.53) | 7.48(3.12 to 13.37) | 9.77(4.67 to 16.66) | 9.62(4.46 to 16.44) | 0.07(0.02 to 0.18) | 0.02(0.01 to 0.05) |
| Bermuda | 3.24(0.58 to 6.93) | 3.21(0.57 to 6.86) | 3.61(0.64 to 7.5) | 3.61(0.64 to 7.49) | 2.35(0.43 to 5.37) | 2.37(0.46 to 5.39) | 0(0 to 0.01) | 0(0 to 0.01) |
| Bhutan | 17.97(13.04 to 23.37) | 17.71(12.82 to 22.96) | 12.91(7.31 to 18.39) | 12.81(7.27 to 18.3) | 14.05(9.32 to 19.97) | 13.84(9.12 to 19.74) | 0.06(0.02 to 0.15) | 0.02(0.01 to 0.04) |
| Bolivia (Plurinational State of) | 16.04(10.38 to 23.1) | 15.29(9.89 to 22.09) | 13.83(8.65 to 20.56) | 13.7(8.55 to 20.42) | 12.52(7.36 to 19.57) | 12.47(7.46 to 19.51) | 0.15(0.03 to 0.45) | 0.03(0.01 to 0.09) |
| Bosnia and Herzegovina | 18.67(14.34 to 23.73) | 18.53(14.24 to 23.55) | 17.28(12.49 to 22.06) | 17.27(12.48 to 22.04) | 14.49(9.3 to 20.28) | 14.51(9.33 to 20.29) | 0.01(0 to 0.03) | 0.01(0 to 0.01) |
| Botswana | 15.24(9.82 to 21.81) | 14.65(9.45 to 20.97) | 13.75(8.92 to 19.43) | 13.73(8.9 to 19.39) | 11.93(7.19 to 18.18) | 11.97(7.24 to 18.15) | 0.08(0.02 to 0.2) | 0.04(0.01 to 0.09) |
| Brazil | 7.46(4.82 to 10.76) | 7.38(4.76 to 10.63) | 7.68(4.96 to 10.9) | 7.63(4.94 to 10.83) | 5.75(3.33 to 9.25) | 5.67(3.31 to 9.09) | 0.06(0.04 to 0.09) | 0.01(0.01 to 0.01) |
| Brunei Darussalam | 3.62(1.01 to 6.9) | 3.56(1 to 6.8) | 4.06(1.16 to 7.6) | 4.04(1.15 to 7.56) | 2.64(0.81 to 5.36) | 2.72(0.87 to 5.41) | 0.03(0 to 0.12) | 0.01(0 to 0.02) |
| Bulgaria | 13.17(9.56 to 16.98) | 13.11(9.52 to 16.9) | 13.31(9.75 to 17.28) | 13.31(9.75 to 17.27) | 9.95(6.2 to 14.84) | 9.96(6.19 to 14.84) | 0.01(0 to 0.04) | 0(0 to 0.01) |
| Burkina Faso | 9.19(3.53 to 17.92) | 7.99(3.1 to 15.55) | 5.24(1.64 to 11.38) | 5.2(1.62 to 11.29) | 6.87(2.24 to 14.39) | 6.75(2.16 to 14.27) | 0.06(0.01 to 0.17) | 0.02(0 to 0.05) |
| Burundi | 6.53(2.53 to 12.65) | 5.94(2.27 to 11.52) | 3.75(1.16 to 8.29) | 3.71(1.15 to 8.2) | 4.94(1.64 to 10.76) | 4.82(1.59 to 10.49) | 0.03(0.01 to 0.08) | 0.02(0 to 0.05) |
| Cabo Verde | 26.84(18.51 to 36.26) | 25.63(17.66 to 34.48) | 21.69(14.42 to 29.14) | 21.54(14.3 to 28.92) | 21.51(13.43 to 31.16) | 21.52(13.58 to 31.18) | 0.06(0.01 to 0.15) | 0.02(0.01 to 0.03) |
| Cambodia | 9.34(5.47 to 14.64) | 9.05(5.3 to 14.2) | 6.71(3.04 to 11.47) | 6.66(3.02 to 11.42) | 7.32(4.13 to 11.67) | 7.22(4.08 to 11.51) | 0.01(0 to 0.03) | 0.01(0 to 0.01) |
| Cameroon | 23.77(15.96 to 32.99) | 21.76(14.44 to 30.07) | 16.21(9.41 to 23.15) | 16.06(9.31 to 22.86) | 19.4(12.24 to 27.56) | 19.19(12.12 to 27.34) | 0.16(0.04 to 0.39) | 0.03(0.02 to 0.06) |
| Canada | 3.05(1.27 to 5.44) | 3.04(1.26 to 5.42) | 3.45(1.5 to 6.03) | 3.45(1.5 to 6.03) | 2.19(0.87 to 4.4) | 2.2(0.87 to 4.41) | 0.01(0 to 0.02) | 0(0 to 0.01) |
| Central African Republic | 8.68(3.05 to 17.33) | 8.17(2.84 to 16.31) | 5.08(1.45 to 11) | 5.07(1.45 to 10.99) | 6.57(2.07 to 13.9) | 6.44(2.07 to 13.86) | 0.04(0.01 to 0.12) | 0.02(0 to 0.05) |
| Chad | 9.56(3.89 to 18.57) | 8.44(3.45 to 16.55) | 5.32(1.69 to 11.44) | 5.26(1.67 to 11.3) | 6.89(2.46 to 15.09) | 6.76(2.35 to 15.1) | 0.05(0.01 to 0.14) | 0.02(0 to 0.04) |
| Chile | 15.47(10.86 to 20.47) | 15.26(10.7 to 20.19) | 15.55(11.22 to 20.54) | 15.53(11.2 to 20.51) | 11.73(7.13 to 17.97) | 11.76(7.14 to 18.05) | 0.02(0 to 0.07) | 0.01(0 to 0.02) |
| China | 25.4(20.73 to 30.82) | 25.37(20.69 to 30.78) | 22.63(16.98 to 28.16) | 22.53(16.91 to 28.06) | 19.88(13.76 to 26.68) | 19.79(13.79 to 26.5) | 0.04(0.02 to 0.07) | 0.02(0.01 to 0.04) |
| Colombia | 14.69(9.96 to 19.97) | 14.47(9.81 to 19.59) | 14.15(10.03 to 19.08) | 14.06(9.97 to 18.96) | 11.19(6.53 to 17.48) | 11.21(6.72 to 17.47) | 0.2(0.05 to 0.42) | 0.02(0 to 0.03) |
| Comoros | 7.06(3.99 to 11.06) | 6.66(3.79 to 10.35) | 4.86(2.23 to 8.41) | 4.82(2.22 to 8.35) | 5.52(3.07 to 8.89) | 5.42(2.99 to 8.71) | 0.02(0.01 to 0.07) | 0.01(0 to 0.03) |
| Congo | 19.68(11.14 to 31.29) | 18.69(10.49 to 29.7) | 15.75(8.59 to 24.45) | 15.69(8.55 to 24.37) | 15.81(8.62 to 26.89) | 15.71(8.61 to 26.87) | 0.13(0.04 to 0.33) | 0.04(0.01 to 0.08) |
| Cook Islands | 2.95(0.57 to 6.88) | 2.91(0.57 to 6.77) | 3.16(0.54 to 7.74) | 3.14(0.53 to 7.7) | 2.15(0.38 to 5.79) | 2.16(0.41 to 5.8) | 0.01(0 to 0.04) | 0(0 to 0.01) |
| Costa Rica | 11.81(7.95 to 16.54) | 11.61(7.83 to 16.22) | 12.16(8.31 to 16.32) | 12.1(8.26 to 16.24) | 8.86(5.19 to 13.77) | 8.9(5.27 to 13.77) | 0.06(0.01 to 0.14) | 0.01(0 to 0.02) |
| Croatia | 12.58(8.83 to 16.74) | 12.52(8.78 to 16.63) | 13.02(9.23 to 17.02) | 13.02(9.22 to 17.01) | 9.42(5.68 to 14.24) | 9.43(5.7 to 14.24) | 0.01(0 to 0.02) | 0(0 to 0.01) |
| Cuba | 11.82(5.35 to 20.6) | 11.75(5.32 to 20.45) | 12.23(5.87 to 20.48) | 12.22(5.86 to 20.46) | 8.85(3.7 to 16.88) | 8.88(3.74 to 16.88) | 0(0 to 0) | 0.01(0 to 0.01) |
| Cyprus | 10.41(7 to 14.4) | 10.34(6.95 to 14.3) | 11.19(7.64 to 15.32) | 11.19(7.63 to 15.31) | 7.72(4.47 to 12.48) | 7.73(4.49 to 12.48) | 0.01(0 to 0.03) | 0.01(0 to 0.01) |
| Czechia | 11.32(7.77 to 15.33) | 11.26(7.73 to 15.25) | 11.98(8.54 to 15.98) | 11.98(8.53 to 15.97) | 8.42(4.98 to 13) | 8.43(5.01 to 13) | 0.01(0 to 0.03) | 0.01(0 to 0.01) |
| Cote d'Ivoire | 17.45(9.69 to 27.05) | 15.89(8.72 to 24.72) | 11.14(5.24 to 18.62) | 11.05(5.2 to 18.48) | 13.81(7.22 to 23.04) | 13.51(6.98 to 22.78) | 0.12(0.03 to 0.31) | 0.03(0.01 to 0.05) |
| Democratic People's Republic of Korea | 18.65(12.83 to 25.24) | 18.6(12.76 to 25.3) | 13.83(8.15 to 19.89) | 13.86(8.15 to 19.88) | 15.14(9.54 to 21.3) | 15.13(9.54 to 21.19) | 0.06(0.02 to 0.15) | 0.03(0.01 to 0.06) |
| Democratic Republic of the Congo | 9.83(4.72 to 16.96) | 9.27(4.46 to 16.14) | 6.29(2.42 to 11.83) | 6.27(2.42 to 11.76) | 7.98(3.57 to 14.39) | 7.82(3.46 to 14.19) | 0.05(0.01 to 0.15) | 0.02(0.01 to 0.04) |
| Denmark | 5.53(2.98 to 8.63) | 5.51(2.97 to 8.6) | 6.17(3.51 to 9.53) | 6.17(3.51 to 9.52) | 4.02(2.09 to 7.16) | 4.02(2.09 to 7.16) | 0(0 to 0.01) | 0.01(0 to 0.02) |
| Djibouti | 22.81(12.38 to 35.5) | 21.29(11.58 to 33.13) | 18.55(9.59 to 28.05) | 18.46(9.52 to 27.95) | 18.08(8.65 to 31.51) | 18.02(8.86 to 31.22) | 0.1(0.03 to 0.28) | 0.06(0.02 to 0.16) |
| Dominica | 12.4(4.75 to 22.56) | 12.21(4.67 to 22.28) | 12.61(5.02 to 22.3) | 12.56(5 to 22.22) | 9.37(3.35 to 18.93) | 9.42(3.56 to 18.92) | 0.02(0 to 0.05) | 0.01(0 to 0.02) |
| Dominican Republic | 11.6(4.98 to 21.38) | 11.14(4.79 to 20.52) | 11.34(4.64 to 20.43) | 11.23(4.6 to 20.18) | 8.84(3.43 to 17.79) | 8.87(3.59 to 17.77) | 0.05(0.01 to 0.16) | 0.01(0 to 0.02) |
| Ecuador | 13.47(8.24 to 19.37) | 12.9(7.88 to 18.54) | 13.54(8.6 to 19.06) | 13.35(8.48 to 18.79) | 10.22(5.52 to 16.46) | 10.25(5.67 to 16.43) | 0.15(0.01 to 0.47) | 0.02(0 to 0.05) |
| Egypt | 35.44(26.77 to 45.47) | 33.99(25.67 to 43.7) | 29.22(21.78 to 36.34) | 28.88(21.56 to 35.96) | 28.91(19.38 to 40.02) | 28.94(19.45 to 40.02) | 0.16(0.06 to 0.39) | 0.03(0.02 to 0.05) |
| El Salvador | 14.52(9.17 to 21.9) | 14.13(8.97 to 21.41) | 13.5(8.14 to 19.99) | 13.35(8.03 to 19.8) | 11.13(5.96 to 17.95) | 11.13(5.97 to 17.93) | 0.05(0.01 to 0.17) | 0.01(0 to 0.02) |
| Equatorial Guinea | 25.33(14.06 to 38.8) | 23.19(12.97 to 35.47) | 21.76(12.93 to 30.77) | 21.56(12.8 to 30.5) | 20.37(10.41 to 33.39) | 20.5(10.83 to 33.31) | 0.17(0.05 to 0.43) | 0.04(0.02 to 0.08) |
| Eritrea | 14.28(7.44 to 23.68) | 13.23(6.88 to 21.85) | 9.1(3.93 to 16.34) | 9.06(3.92 to 16.27) | 11.49(5.53 to 19.15) | 11.36(5.28 to 19.2) | 0.05(0.01 to 0.13) | 0.02(0.01 to 0.06) |
| Estonia | 2.51(0.87 to 4.62) | 2.48(0.86 to 4.58) | 2.73(0.98 to 4.93) | 2.73(0.98 to 4.93) | 1.81(0.59 to 3.59) | 1.83(0.62 to 3.62) | 0.01(0 to 0.03) | 0(0 to 0.01) |
| Eswatini | 13.16(8.5 to 18.97) | 12.56(8.04 to 18.12) | 11.04(6.56 to 16.19) | 11.05(6.59 to 16.2) | 10.34(6.25 to 15.71) | 10.3(6.28 to 15.7) | 0.1(0.02 to 0.27) | 0.04(0.01 to 0.1) |
| Ethiopia | 8.11(4.25 to 13.51) | 7.36(3.92 to 12.13) | 5.38(2.45 to 9.75) | 5.37(2.46 to 9.65) | 5.88(2.67 to 10.51) | 5.49(2.4 to 10.12) | 0.03(0.01 to 0.09) | 0.02(0.01 to 0.06) |
| Fiji | 7.07(2.19 to 16.39) | 6.8(2.11 to 15.73) | 6.77(1.99 to 15.16) | 6.72(1.97 to 15.05) | 5.44(1.49 to 13.19) | 5.45(1.51 to 13.16) | 0.02(0 to 0.06) | 0.01(0 to 0.01) |
| Finland | 1.63(0.3 to 3.53) | 1.62(0.29 to 3.5) | 1.86(0.35 to 4.05) | 1.86(0.35 to 4.05) | 1.16(0.23 to 2.87) | 1.17(0.23 to 2.87) | 0(0 to 0) | 0(0 to 0.01) |
| France | 6.97(4.31 to 10.29) | 6.92(4.28 to 10.23) | 7.68(4.92 to 11.21) | 7.68(4.92 to 11.2) | 5.09(2.84 to 8.48) | 5.1(2.85 to 8.48) | 0.04(0 to 0.12) | 0.01(0 to 0.02) |
| Gabon | 22.68(12.81 to 35.35) | 21.61(12.24 to 33.76) | 20.86(12.57 to 29.99) | 20.79(12.54 to 29.87) | 17.81(9.29 to 29.88) | 17.91(9.69 to 29.86) | 0.12(0.03 to 0.36) | 0.04(0.02 to 0.09) |
| Gambia | 15.62(8.6 to 25.17) | 14.35(7.91 to 23.19) | 9.38(4.33 to 15.66) | 9.31(4.29 to 15.51) | 12.67(6.58 to 20.85) | 12.41(6.29 to 20.44) | 0.06(0.02 to 0.16) | 0.02(0.01 to 0.04) |
| Georgia | 11.83(7.96 to 16.81) | 11.68(7.86 to 16.62) | 11.61(7.71 to 16.53) | 11.59(7.7 to 16.5) | 9.04(5.29 to 14.15) | 9.06(5.34 to 14.22) | 0.05(0.01 to 0.11) | 0.01(0 to 0.01) |
| Germany | 7.31(4.57 to 10.65) | 7.28(4.55 to 10.6) | 8.05(5.24 to 11.61) | 8.05(5.24 to 11.61) | 5.35(3 to 8.82) | 5.35(3 to 8.83) | 0(0 to 0.02) | 0.01(0 to 0.02) |
| Ghana | 23.92(16.25 to 33.03) | 22.29(15.03 to 30.72) | 17.5(10.79 to 24.91) | 17.17(10.58 to 24.46) | 19.49(12.38 to 29.07) | 19.27(12.3 to 28.98) | 0.31(0.07 to 0.93) | 0.04(0.01 to 0.12) |
| Greece | 9.36(6.24 to 13.22) | 9.32(6.21 to 13.15) | 10.1(6.8 to 13.97) | 10.09(6.8 to 13.96) | 6.9(3.99 to 10.87) | 6.91(4 to 10.87) | 0(0 to 0) | 0.01(0 to 0.01) |
| Greenland | 2.69(0.27 to 7.57) | 2.68(0.27 to 7.53) | 2.99(0.29 to 8.52) | 2.99(0.29 to 8.51) | 1.96(0.21 to 5.81) | 1.99(0.25 to 5.81) | 0.02(0 to 0.08) | 0.01(0 to 0.02) |
| Grenada | 14.26(4.84 to 26.79) | 13.96(4.72 to 26.31) | 14.44(5.71 to 25.38) | 14.39(5.69 to 25.31) | 10.81(3.49 to 21.43) | 10.86(3.63 to 21.43) | 0.02(0 to 0.06) | 0.01(0 to 0.02) |
| Guam | 4.44(1.69 to 8.09) | 4.32(1.65 to 7.88) | 4.87(2.05 to 8.6) | 4.83(2.04 to 8.54) | 3.33(1.27 to 6.85) | 3.46(1.34 to 6.96) | 0.03(0 to 0.12) | 0.01(0 to 0.02) |
| Guatemala | 14.28(9.78 to 19.97) | 13.34(9.09 to 18.52) | 11.11(6.4 to 16.37) | 10.76(6.24 to 15.82) | 11.41(7.09 to 16.92) | 11.33(7 to 16.92) | 0.12(0.05 to 0.22) | 0.03(0.01 to 0.06) |
| Guinea | 10.89(5.2 to 18.83) | 9.88(4.75 to 17.04) | 6.36(2.45 to 12.27) | 6.32(2.44 to 12.21) | 8.36(3.55 to 15.58) | 8.18(3.36 to 15.46) | 0.05(0.01 to 0.15) | 0.02(0 to 0.04) |
| Guinea-Bissau | 12.26(5.93 to 21.05) | 11.26(5.5 to 19.34) | 7.2(2.85 to 13.16) | 7.13(2.82 to 13.04) | 9.75(4.38 to 17.28) | 9.54(4.27 to 16.92) | 0.07(0.02 to 0.18) | 0.02(0.01 to 0.04) |
| Guyana | 13.17(5.02 to 25.05) | 12.71(4.85 to 24.33) | 13.05(4.96 to 23.7) | 12.97(4.94 to 23.55) | 10.06(3.27 to 20.43) | 10.1(3.4 to 20.44) | 0.02(0.01 to 0.04) | 0.01(0 to 0.02) |
| Haiti | 6.92(3.04 to 13.18) | 6.42(2.82 to 12.41) | 4.78(1.71 to 9.47) | 4.72(1.68 to 9.35) | 5.46(2.38 to 10.65) | 5.38(2.32 to 10.52) | 0.02(0 to 0.05) | 0.01(0 to 0.02) |
| Honduras | 11.33(7.74 to 15.85) | 10.92(7.45 to 15.26) | 8(4.24 to 12.66) | 7.94(4.22 to 12.58) | 9.07(5.67 to 13.7) | 8.86(5.51 to 13.4) | 0.18(0.05 to 0.47) | 0.03(0.01 to 0.08) |
| Hungary | 11.21(7.92 to 14.91) | 11.17(7.89 to 14.83) | 11.58(8.29 to 15.26) | 11.58(8.29 to 15.26) | 8.41(5.07 to 12.82) | 8.43(5.1 to 12.82) | 0.02(0 to 0.05) | 0.01(0 to 0.01) |
| Iceland | 1.73(0.34 to 3.78) | 1.71(0.34 to 3.74) | 1.97(0.38 to 4.39) | 1.97(0.38 to 4.39) | 1.23(0.25 to 3) | 1.25(0.27 to 3.01) | 0.01(0 to 0.02) | 0.01(0 to 0.02) |
| India | 30.17(24.64 to 35.92) | 29.91(24.56 to 35.54) | 21.29(15.38 to 26.85) | 21.26(15.37 to 26.81) | 24.37(18.15 to 30.63) | 24.57(17.99 to 31.26) | 0.12(0.07 to 0.21) | 0.02(0.01 to 0.04) |
| Indonesia | 12.13(8.55 to 16.34) | 11.86(8.36 to 16.03) | 11.47(7.71 to 15.46) | 11.43(7.72 to 15.38) | 9.28(5.89 to 13.74) | 9.28(5.98 to 13.6) | 0.01(0 to 0.02) | 0.01(0 to 0.01) |
| Iran (Islamic Republic of) | 23.53(17.73 to 29.58) | 22.86(17.27 to 28.63) | 22.09(16.4 to 27.6) | 21.88(16.25 to 27.32) | 18.84(12.28 to 26.53) | 19.1(12.61 to 26.8) | 0.17(0.03 to 0.4) | 0.02(0.01 to 0.03) |
| Iraq | 28.45(21.13 to 37.1) | 26.11(19.45 to 34.07) | 25.22(18.72 to 31.91) | 25(18.57 to 31.63) | 22.78(14.68 to 32.36) | 22.83(15.01 to 32.36) | 0.17(0.05 to 0.52) | 0.03(0.02 to 0.05) |
| Ireland | 3.74(1.74 to 6.32) | 3.72(1.72 to 6.29) | 4.23(2.03 to 6.99) | 4.23(2.02 to 6.98) | 2.69(1.21 to 5.09) | 2.7(1.23 to 5.1) | 0(0 to 0.01) | 0.01(0 to 0.02) |
| Israel | 13.51(9.36 to 18.17) | 13.29(9.2 to 17.83) | 14.01(10.06 to 18.37) | 13.98(10.04 to 18.34) | 10.12(6.15 to 15.29) | 10.15(6.19 to 15.31) | 0.07(0 to 0.25) | 0.02(0 to 0.03) |
| Italy | 10.26(6.97 to 14.06) | 10.29(7.02 to 14.09) | 11.33(8.06 to 15.28) | 11.25(8 to 15.21) | 8.15(4.86 to 12.51) | 8.09(4.83 to 12.43) | 0.01(0.01 to 0.02) | 0.01(0 to 0.01) |
| Jamaica | 10.21(6.09 to 15.27) | 9.99(5.98 to 14.97) | 10.45(6.04 to 15.61) | 10.39(6 to 15.51) | 7.67(4.06 to 13.12) | 7.73(4.24 to 13.11) | 0.01(0 to 0.01) | 0.01(0 to 0.01) |
| Japan | 8.59(5.18 to 12.64) | 8.57(5.17 to 12.61) | 9.34(5.76 to 13.81) | 9.35(5.76 to 13.81) | 6.32(3.36 to 10.36) | 6.34(3.38 to 10.39) | 0(0 to 0) | 0(0 to 0.01) |
| Jordan | 20.33(15.14 to 26.28) | 19.05(14.16 to 24.65) | 19.92(14.78 to 25.31) | 19.74(14.67 to 25.12) | 15.75(9.97 to 23.36) | 15.81(10.16 to 23.36) | 0.06(0.02 to 0.14) | 0.02(0.01 to 0.03) |
| Kazakhstan | 13.61(8.07 to 20.3) | 13.47(8 to 20.11) | 13.43(8.55 to 19.13) | 13.4(8.53 to 19.09) | 10.37(5.48 to 16.77) | 10.44(5.72 to 16.76) | 0.06(0 to 0.15) | 0.01(0 to 0.03) |
| Kenya | 8.97(5.63 to 13.42) | 8.29(5.21 to 12.35) | 7.18(3.89 to 10.93) | 7.17(3.91 to 10.92) | 6.84(4.11 to 10.7) | 6.55(3.88 to 10.35) | 0.03(0.01 to 0.06) | 0.01(0.01 to 0.02) |
| Kiribati | 4.42(1.51 to 10.02) | 4.22(1.45 to 9.55) | 3.15(0.9 to 7.37) | 3.1(0.89 to 7.3) | 3.46(1.18 to 7.92) | 3.44(1.17 to 7.88) | 0.02(0 to 0.08) | 0.01(0 to 0.03) |
| Kuwait | 33.71(26.99 to 41.49) | 32.05(25.5 to 39.5) | 28.67(21.83 to 35.26) | 28.57(21.77 to 35.12) | 27.09(18.56 to 36.93) | 27.11(18.64 to 36.93) | 0.06(0.03 to 0.11) | 0.02(0.01 to 0.04) |
| Kyrgyzstan | 14.92(9.13 to 22.5) | 14.61(8.94 to 21.99) | 13.25(7.66 to 19.39) | 13.2(7.63 to 19.3) | 11.53(6.28 to 18.67) | 11.51(6.35 to 18.65) | 0.13(0.02 to 0.31) | 0.06(0.01 to 0.14) |
| Lao People's Democratic Republic | 8.57(5.15 to 12.7) | 8.21(4.94 to 12.14) | 6.26(3.11 to 10.4) | 6.19(3.07 to 10.31) | 6.74(3.81 to 10.26) | 6.65(3.86 to 10.25) | 0.01(0 to 0.03) | 0.01(0 to 0.01) |
| Latvia | 7.63(4.76 to 11.27) | 7.57(4.72 to 11.18) | 8.21(5.22 to 11.83) | 8.21(5.21 to 11.82) | 5.63(3.13 to 9.13) | 5.67(3.18 to 9.14) | 0(0 to 0.01) | 0(0 to 0.01) |
| Lebanon | 19.39(13.03 to 26.16) | 18.93(12.72 to 25.59) | 19.25(13.35 to 25.02) | 19.22(13.32 to 24.97) | 14.9(9.01 to 22.6) | 14.96(9.17 to 22.61) | 0.04(0.01 to 0.11) | 0.02(0 to 0.04) |
| Lesotho | 13.28(8.42 to 19.28) | 12.9(8.13 to 18.74) | 10.19(5.37 to 15.79) | 10.22(5.38 to 15.87) | 10.62(6.46 to 16.13) | 10.56(6.38 to 16.16) | 0.07(0.02 to 0.17) | 0.04(0.01 to 0.09) |
| Liberia | 12.62(6.87 to 20.54) | 11.32(6.19 to 18.65) | 7.72(3.34 to 14.03) | 7.63(3.3 to 13.87) | 10.26(4.98 to 17.51) | 10.04(4.84 to 17.25) | 0.07(0.02 to 0.21) | 0.01(0.01 to 0.03) |
| Libya | 24.2(15.94 to 32.95) | 23.35(15.37 to 31.79) | 22.71(15.14 to 29.81) | 22.66(15.1 to 29.76) | 19(11.08 to 29.31) | 19.02(11.15 to 29.31) | 0.03(0.01 to 0.07) | 0.01(0.01 to 0.02) |
| Lithuania | 6.16(3.58 to 9.44) | 6.12(3.56 to 9.38) | 6.79(4.02 to 10.24) | 6.78(4.02 to 10.23) | 4.51(2.33 to 7.63) | 4.54(2.38 to 7.62) | 0.01(0 to 0.03) | 0(0 to 0.01) |
| Luxembourg | 5.79(3.29 to 8.76) | 5.74(3.26 to 8.69) | 6.46(3.78 to 9.66) | 6.45(3.77 to 9.65) | 4.21(2.2 to 7.31) | 4.22(2.21 to 7.31) | 0.01(0 to 0.03) | 0.01(0 to 0.02) |
| Madagascar | 5.53(2.96 to 9.02) | 5.03(2.71 to 8.25) | 3.67(1.61 to 6.59) | 3.62(1.58 to 6.5) | 4.42(2.23 to 7.62) | 4.33(2.18 to 7.4) | 0.03(0.01 to 0.08) | 0.01(0 to 0.03) |
| Malawi | 6.14(3.05 to 10.83) | 5.54(2.73 to 9.8) | 3.79(1.48 to 7.43) | 3.76(1.47 to 7.37) | 4.84(2.19 to 8.65) | 4.74(2.09 to 8.65) | 0.02(0.01 to 0.06) | 0.01(0 to 0.03) |
| Malaysia | 11.14(7.18 to 15.98) | 10.9(7.02 to 15.62) | 11.78(7.6 to 16.26) | 11.75(7.57 to 16.21) | 8.29(4.69 to 13.2) | 8.31(4.73 to 13.2) | 0.01(0 to 0.03) | 0.01(0 to 0.02) |
| Maldives | 6.94(4.48 to 10.07) | 6.75(4.36 to 9.79) | 6.75(4.06 to 10.07) | 6.71(4.03 to 9.99) | 5.19(2.97 to 8.57) | 5.22(3.04 to 8.56) | 0.01(0 to 0.02) | 0.01(0 to 0.01) |
| Mali | 10.2(3.93 to 19.24) | 9.38(3.63 to 17.85) | 5.53(1.7 to 11.81) | 5.47(1.69 to 11.68) | 7.37(2.49 to 15.52) | 7.28(2.39 to 15.7) | 0.05(0.01 to 0.14) | 0.02(0 to 0.05) |
| Malta | 8.32(5.26 to 11.88) | 8.27(5.22 to 11.79) | 9.1(5.94 to 12.8) | 9.09(5.94 to 12.79) | 6.12(3.42 to 9.92) | 6.13(3.45 to 9.92) | 0.02(0 to 0.06) | 0.01(0 to 0.02) |
| Marshall Islands | 5.53(2.17 to 11.34) | 5.3(2.08 to 10.87) | 4.69(1.59 to 10.12) | 4.63(1.57 to 9.97) | 4.26(1.59 to 8.92) | 4.25(1.58 to 8.89) | 0.02(0 to 0.09) | 0(0 to 0.01) |
| Mauritania | 26.33(16.29 to 38.16) | 24.21(14.99 to 34.85) | 18.17(10.32 to 26.54) | 17.99(10.2 to 26.31) | 21.29(12.25 to 32.72) | 20.96(11.95 to 32.06) | 0.2(0.04 to 0.52) | 0.03(0.02 to 0.06) |
| Mauritius | 9.8(4.14 to 16.38) | 9.61(4.05 to 16.07) | 10.46(4.9 to 17.38) | 10.41(4.88 to 17.31) | 7.29(3.06 to 13.77) | 7.32(3.11 to 13.79) | 0(0 to 0.01) | 0.01(0 to 0.01) |
| Mexico | 13.13(9.03 to 17.85) | 12.88(8.86 to 17.49) | 12.75(8.9 to 16.89) | 12.68(8.87 to 16.79) | 10.11(6.29 to 15.11) | 10.16(6.48 to 14.96) | 0.31(0.18 to 0.46) | 0.04(0.02 to 0.06) |
| Micronesia (Federated States of) | 6.44(2.13 to 14.56) | 6.2(2.05 to 14) | 5.74(1.67 to 13.12) | 5.68(1.66 to 12.99) | 5(1.52 to 11.69) | 5(1.52 to 11.65) | 0.02(0 to 0.06) | 0(0 to 0.01) |
| Monaco | 7.19(3.92 to 11.59) | 7.16(3.9 to 11.55) | 7.93(4.36 to 11.85) | 7.92(4.36 to 11.84) | 5.25(2.54 to 9.2) | 5.25(2.54 to 9.2) | 0(0 to 0.01) | 0.01(0 to 0.01) |
| Mongolia | 21.24(14.84 to 28.65) | 20.53(14.31 to 27.75) | 17.85(11.95 to 23.95) | 17.81(11.95 to 23.91) | 16.87(10.94 to 24.16) | 16.82(10.95 to 24.15) | 0.6(0.11 to 1.82) | 0.08(0.01 to 0.29) |
| Montenegro | 14.3(10.13 to 18.64) | 14.06(9.96 to 18.33) | 14.14(10.12 to 18.33) | 14.12(10.11 to 18.31) | 10.9(6.75 to 15.91) | 10.92(6.78 to 15.92) | 0.04(0 to 0.19) | 0.01(0 to 0.01) |
| Morocco | 22.3(16.02 to 28.33) | 21.56(15.5 to 27.35) | 20.97(15.27 to 26.43) | 20.94(15.24 to 26.4) | 17.39(10.91 to 25.08) | 17.45(11.22 to 25.05) | 0.1(0.03 to 0.26) | 0.02(0.01 to 0.03) |
| Mozambique | 4.85(2.39 to 8.59) | 4.36(2.15 to 7.77) | 2.79(1.01 to 5.67) | 2.76(1 to 5.61) | 3.69(1.57 to 6.93) | 3.61(1.48 to 6.81) | 0.01(0 to 0.04) | 0.01(0 to 0.03) |
| Myanmar | 13.81(10.05 to 18.2) | 13.53(9.87 to 17.83) | 10.5(6.21 to 15.2) | 10.43(6.16 to 15.1) | 10.96(7.42 to 15.02) | 10.8(7.24 to 14.87) | 0.01(0 to 0.04) | 0.01(0 to 0.02) |
| Namibia | 14.36(8.85 to 21.33) | 13.84(8.54 to 20.61) | 12.47(7.27 to 18.25) | 12.43(7.23 to 18.21) | 11.24(6.36 to 17.39) | 11.21(6.32 to 17.23) | 0.09(0.03 to 0.22) | 0.04(0.02 to 0.08) |
| Nauru | 2.57(0.63 to 5.89) | 2.43(0.6 to 5.56) | 2.64(0.65 to 6.46) | 2.59(0.64 to 6.29) | 2.07(0.62 to 5.19) | 2.13(0.68 to 5.2) | 0.04(0 to 0.15) | 0.01(0 to 0.02) |
| Nepal | 25.88(17.68 to 34.15) | 25.58(17.46 to 33.8) | 16.2(9.5 to 23.07) | 16.07(9.41 to 22.8) | 20.48(12.9 to 28.78) | 20.13(12.49 to 28.35) | 0.1(0.03 to 0.24) | 0.02(0.01 to 0.05) |
| Netherlands | 7.52(4.78 to 10.88) | 7.49(4.76 to 10.84) | 8.28(5.34 to 11.91) | 8.28(5.33 to 11.9) | 5.5(3.08 to 9.01) | 5.51(3.08 to 9.02) | 0.03(0 to 0.11) | 0.01(0 to 0.02) |
| New Zealand | 2.06(0.35 to 4.5) | 2.05(0.35 to 4.48) | 2.34(0.41 to 5) | 2.34(0.41 to 5) | 1.47(0.25 to 3.49) | 1.55(0.33 to 3.66) | 0.02(0 to 0.07) | 0.01(0 to 0.02) |
| Nicaragua | 11.24(7.2 to 16.7) | 10.92(7.01 to 16.24) | 8.85(4.58 to 13.97) | 8.73(4.53 to 13.81) | 8.75(5.04 to 13.97) | 8.66(5 to 13.67) | 0.06(0.01 to 0.15) | 0.01(0 to 0.01) |
| Niger | 8.66(2.53 to 20.96) | 7.58(2.23 to 18.34) | 4.78(1.08 to 12.35) | 4.72(1.06 to 12.22) | 6.1(1.61 to 15.52) | 6.02(1.56 to 15.55) | 0.05(0.01 to 0.17) | 0.02(0 to 0.06) |
| Nigeria | 22.37(15.03 to 31.48) | 20.13(13.65 to 28.27) | 16.13(9.71 to 22.8) | 15.82(9.49 to 22.34) | 14.82(8.48 to 23.5) | 14.12(7.72 to 23.13) | 0.13(0.04 to 0.27) | 0.03(0.02 to 0.05) |
| Niue | 3.31(0.7 to 7.74) | 3.26(0.69 to 7.63) | 3.55(0.74 to 8.54) | 3.52(0.73 to 8.49) | 2.47(0.55 to 6.71) | 2.55(0.65 to 6.7) | 0.02(0 to 0.07) | 0(0 to 0.01) |
| North Macedonia | 19.52(14.58 to 24.33) | 19.31(14.44 to 24.11) | 18.46(13.63 to 23.33) | 18.43(13.61 to 23.3) | 15.12(9.66 to 21.4) | 15.12(9.7 to 21.41) | 0.01(0 to 0.02) | 0.01(0 to 0.01) |
| Northern Mariana Islands | 4.89(2.35 to 8.28) | 4.8(2.32 to 8.14) | 5.13(2.6 to 8.44) | 5.1(2.58 to 8.38) | 3.64(1.62 to 7.11) | 3.68(1.69 to 7.13) | 0.01(0 to 0.06) | 0(0 to 0.01) |
| Norway | 2.49(0.84 to 4.72) | 2.49(0.85 to 4.72) | 2.82(0.95 to 5.39) | 2.83(0.96 to 5.39) | 1.76(0.57 to 3.84) | 1.77(0.58 to 3.86) | 0.01(0 to 0.01) | 0(0 to 0.01) |
| Oman | 26.93(18.57 to 36.42) | 25.44(17.47 to 34.22) | 24.61(17.41 to 31.72) | 24.46(17.34 to 31.56) | 21.4(12.79 to 31.32) | 21.43(12.93 to 31.32) | 0.1(0.03 to 0.31) | 0.02(0.01 to 0.03) |
| Pakistan | 24.55(18.37 to 31.47) | 23.67(17.79 to 30.19) | 17.69(11.43 to 24.24) | 17.61(11.38 to 24.14) | 19.16(12.84 to 26.11) | 18.92(12.57 to 26.09) | 0.17(0.08 to 0.36) | 0.04(0.02 to 0.08) |
| Palau | 2.79(0.01 to 7.54) | 2.76(0.01 to 7.45) | 3.16(0.01 to 7.93) | 3.15(0.01 to 7.9) | 2.06(0.05 to 5.96) | 2.13(0.1 to 6.07) | 0.01(0 to 0.05) | 0(0 to 0.01) |
| Palestine | 20.49(14 to 28.27) | 19.06(13.05 to 26.2) | 19.89(14.24 to 25.81) | 19.64(14.04 to 25.5) | 15.92(9.55 to 24.71) | 15.96(9.59 to 24.72) | 0.05(0.02 to 0.11) | 0.02(0.01 to 0.04) |
| Panama | 8.68(4.81 to 12.8) | 8.41(4.66 to 12.42) | 8.93(5.34 to 13.08) | 8.88(5.3 to 12.99) | 6.51(3.32 to 10.93) | 6.57(3.31 to 10.93) | 0.2(0 to 0.57) | 0.05(0 to 0.15) |
| Papua New Guinea | 5.12(1.39 to 13.29) | 4.85(1.33 to 12.75) | 3.42(0.82 to 9.2) | 3.35(0.81 to 9.06) | 4.02(1.05 to 10.63) | 3.98(1.03 to 10.62) | 0.03(0 to 0.11) | 0(0 to 0.01) |
| Paraguay | 8.13(5.1 to 12.22) | 7.87(4.93 to 11.82) | 7.57(4.41 to 11.42) | 7.53(4.38 to 11.35) | 6.2(3.53 to 10.38) | 6.2(3.55 to 10.44) | 0.03(0 to 0.12) | 0.01(0 to 0.02) |
| Peru | 19.15(13.13 to 25.79) | 18.2(12.49 to 24.54) | 17.21(11.55 to 23.36) | 17.01(11.44 to 23.05) | 14.98(9.27 to 22.66) | 14.99(9.37 to 22.59) | 0.18(0.04 to 0.5) | 0.02(0.01 to 0.04) |
| Philippines | 11.11(8.21 to 14.7) | 10.9(8.08 to 14.37) | 10.68(7.38 to 14.18) | 10.64(7.37 to 14.12) | 8.4(5.55 to 11.89) | 8.41(5.57 to 11.88) | 0.02(0 to 0.04) | 0.01(0.01 to 0.01) |
| Poland | 15.15(11.21 to 19.61) | 15.23(11.32 to 19.68) | 15.22(11.28 to 19.31) | 15.2(11.26 to 19.29) | 11.55(7.2 to 17) | 11.6(7.27 to 17.07) | 0.02(0.01 to 0.03) | 0.01(0 to 0.01) |
| Portugal | 4.16(2.11 to 6.78) | 4.14(2.09 to 6.76) | 4.65(2.44 to 7.39) | 4.65(2.43 to 7.38) | 3(1.43 to 5.48) | 3.01(1.44 to 5.48) | 0(0 to 0.01) | 0(0 to 0.01) |
| Puerto Rico | 2.76(0.59 to 5.37) | 2.74(0.58 to 5.33) | 3.15(0.65 to 6.15) | 3.14(0.65 to 6.14) | 1.98(0.43 to 4.34) | 2.02(0.47 to 4.38) | 0(0 to 0.01) | 0(0 to 0.01) |
| Qatar | 37.79(29.52 to 46.88) | 35.99(28 to 44.72) | 30.57(22.86 to 37.84) | 30.42(22.75 to 37.68) | 31.15(22.08 to 41.06) | 31.15(22.11 to 41.06) | 0.05(0.02 to 0.12) | 0.02(0.01 to 0.03) |
| Republic of Korea | 18.6(13.11 to 24.59) | 18.43(12.99 to 24.39) | 18.57(13.43 to 24.11) | 18.55(13.43 to 24.1) | 14.17(8.6 to 21.14) | 14.17(8.6 to 21.14) | 0.01(0 to 0.02) | 0.01(0 to 0.02) |
| Republic of Moldova | 9.05(4.74 to 14.55) | 8.98(4.71 to 14.42) | 9.5(5.11 to 14.83) | 9.48(5.11 to 14.82) | 6.8(3.46 to 11.93) | 6.96(3.66 to 11.97) | 0.32(0 to 1.12) | 0.01(0 to 0.05) |
| Romania | 10.59(7.39 to 14.17) | 10.53(7.34 to 14.11) | 11.01(7.95 to 14.57) | 11(7.94 to 14.56) | 7.92(4.77 to 12.11) | 7.95(4.83 to 12.1) | 0.04(0 to 0.11) | 0(0 to 0.01) |
| Russian Federation | 7.22(3.74 to 11.57) | 7.15(3.71 to 11.45) | 7.64(4.07 to 11.49) | 7.62(4.05 to 11.47) | 5.25(2.58 to 9.11) | 5.27(2.63 to 9.14) | 0.1(0.06 to 0.14) | 0.02(0.01 to 0.03) |
| Rwanda | 11.21(5.46 to 19.68) | 10.36(5.08 to 18.13) | 7.3(2.96 to 13.61) | 7.25(2.94 to 13.51) | 9.14(4.2 to 16.58) | 9.03(4.09 to 16.51) | 0.05(0.01 to 0.13) | 0.02(0.01 to 0.07) |
| Saint Kitts and Nevis | 4.8(1.94 to 8.29) | 4.72(1.91 to 8.13) | 5.3(2.27 to 9.34) | 5.29(2.26 to 9.32) | 3.53(1.39 to 6.7) | 3.63(1.51 to 6.73) | 0.01(0 to 0.05) | 0.01(0 to 0.02) |
| Saint Lucia | 14.14(5.5 to 25.41) | 13.93(5.43 to 25.01) | 14.32(6.01 to 24.81) | 14.29(5.99 to 24.74) | 10.71(3.75 to 21.06) | 10.76(3.86 to 21.06) | 0.01(0 to 0.04) | 0.01(0 to 0.02) |
| Saint Vincent and the Grenadines | 14(4.78 to 26.57) | 13.71(4.69 to 26.14) | 14.05(5.04 to 24.85) | 14.01(5.02 to 24.78) | 10.6(3.36 to 21.19) | 10.65(3.56 to 21.19) | 0.02(0 to 0.05) | 0.01(0 to 0.02) |
| Samoa | 6.22(1.91 to 14.12) | 5.96(1.83 to 13.56) | 5.27(1.5 to 12.13) | 5.26(1.49 to 12.1) | 4.91(1.34 to 11.57) | 4.89(1.34 to 11.67) | 0.01(0 to 0.05) | 0(0 to 0.01) |
| San Marino | 5.62(2.1 to 9.95) | 5.58(2.08 to 9.89) | 6.26(2.65 to 10.8) | 6.26(2.64 to 10.79) | 4.09(1.49 to 7.86) | 4.09(1.51 to 7.88) | 0.01(0 to 0.03) | 0.01(0 to 0.02) |
| Sao Tome and Principe | 14.76(8.56 to 23.35) | 14.09(8.17 to 22.21) | 11.04(5.44 to 18.03) | 10.84(5.35 to 17.7) | 11.74(6.34 to 18.77) | 11.64(6.24 to 18.55) | 0.08(0.02 to 0.19) | 0.01(0.01 to 0.03) |
| Saudi Arabia | 33.62(26.03 to 42.01) | 32.58(25.29 to 40.84) | 28.24(21.08 to 35.13) | 28.13(21.01 to 34.99) | 27.05(18.41 to 37.33) | 27.05(18.41 to 37.33) | 0.05(0.01 to 0.13) | 0.02(0.01 to 0.03) |
| Senegal | 16.95(9.58 to 27.68) | 15.61(8.77 to 25.69) | 10.36(4.83 to 17.4) | 10.28(4.79 to 17.25) | 13.3(6.79 to 21.65) | 13.03(6.52 to 21.48) | 0.1(0.03 to 0.28) | 0.02(0.01 to 0.05) |
| Serbia | 16.9(12.36 to 21.67) | 16.79(12.27 to 21.53) | 16.47(11.92 to 20.97) | 16.46(11.92 to 20.96) | 12.95(8.16 to 19.01) | 12.96(8.18 to 19.02) | 0.01(0 to 0.01) | 0.01(0 to 0.01) |
| Seychelles | 10.32(4.74 to 17.22) | 10.13(4.65 to 16.92) | 11.03(5.67 to 18.1) | 11(5.65 to 18.03) | 7.67(3.36 to 14.3) | 7.72(3.45 to 14.31) | 0.01(0 to 0.03) | 0.01(0 to 0.02) |
| Sierra Leone | 11.42(5.6 to 19.97) | 10.33(5.08 to 18.06) | 6.79(2.77 to 13.06) | 6.71(2.75 to 12.9) | 8.97(4.01 to 16.56) | 8.83(3.85 to 16.34) | 0.05(0.01 to 0.12) | 0.02(0.01 to 0.04) |
| Singapore | 12.75(7.48 to 18.7) | 12.67(7.43 to 18.63) | 13.36(8.19 to 19.37) | 13.35(8.18 to 19.35) | 9.54(5.01 to 16.23) | 9.54(5.02 to 16.23) | 0.01(0 to 0.04) | 0.01(0 to 0.02) |
| Slovakia | 12.55(8.76 to 16.83) | 12.42(8.68 to 16.64) | 13.22(9.59 to 17.41) | 13.21(9.59 to 17.4) | 9.37(5.54 to 14.45) | 9.38(5.55 to 14.46) | 0(0 to 0.02) | 0(0 to 0.01) |
| Slovenia | 11.55(8.08 to 15.36) | 11.47(8.03 to 15.27) | 12.13(8.66 to 16.12) | 12.12(8.66 to 16.11) | 8.61(5.27 to 13.25) | 8.62(5.27 to 13.25) | 0.02(0 to 0.06) | 0(0 to 0.01) |
| Solomon Islands | 4.34(1.34 to 10.15) | 4.14(1.27 to 9.63) | 2.92(0.75 to 7.51) | 2.87(0.74 to 7.4) | 3.51(1.07 to 8.27) | 3.46(1.07 to 8.07) | 0.02(0 to 0.07) | 0(0 to 0.01) |
| Somalia | 2.49(0.57 to 6.78) | 2.23(0.48 to 6.09) | 1.36(0.24 to 4.21) | 1.35(0.24 to 4.17) | 1.69(0.36 to 5.09) | 1.66(0.36 to 4.97) | 0.01(0 to 0.03) | 0.01(0 to 0.02) |
| South Africa | 17.95(12.65 to 23.91) | 17.66(12.53 to 23.38) | 16.44(11.95 to 22.1) | 16.42(11.95 to 22.04) | 14.33(9.07 to 20.87) | 14.53(9.42 to 20.93) | 0.1(0.06 to 0.16) | 0.04(0.03 to 0.07) |
| South Sudan | 10.34(5.28 to 17.45) | 9.14(4.65 to 15.62) | 6.33(2.58 to 11.44) | 6.25(2.55 to 11.34) | 7.92(3.69 to 14.14) | 7.79(3.6 to 14.06) | 0.04(0.01 to 0.14) | 0.02(0.01 to 0.08) |
| Spain | 5.52(3.17 to 8.45) | 5.5(3.16 to 8.42) | 6.13(3.66 to 9.27) | 6.12(3.66 to 9.26) | 4.02(2.11 to 6.92) | 4.02(2.12 to 6.93) | 0(0 to 0.01) | 0(0 to 0.01) |
| Sri Lanka | 12.59(8.15 to 17.64) | 12.37(8.01 to 17.35) | 11.67(7.46 to 16.59) | 11.62(7.43 to 16.51) | 9.78(5.71 to 15.07) | 9.79(5.73 to 15.06) | 0.01(0 to 0.02) | 0.01(0 to 0.01) |
| Sudan | 24.46(15.38 to 35.64) | 22.31(13.96 to 32.61) | 18.36(10.9 to 26.55) | 18.04(10.67 to 26.11) | 19.9(11.6 to 30.46) | 19.74(11.56 to 30.14) | 0.11(0.03 to 0.36) | 0.02(0.01 to 0.04) |
| Suriname | 13.91(5.91 to 24.95) | 13.58(5.77 to 24.35) | 13.61(5.92 to 23.45) | 13.56(5.91 to 23.33) | 10.64(4.24 to 20.51) | 10.68(4.36 to 20.48) | 0.03(0 to 0.1) | 0.01(0 to 0.02) |
| Sweden | 1.72(0.42 to 3.71) | 1.71(0.42 to 3.67) | 1.96(0.47 to 4.23) | 1.95(0.47 to 4.23) | 1.23(0.29 to 3.03) | 1.24(0.31 to 3.04) | 0(0 to 0) | 0.01(0 to 0.01) |
| Switzerland | 5.63(3.24 to 8.64) | 5.6(3.22 to 8.58) | 6.28(3.75 to 9.4) | 6.27(3.75 to 9.4) | 4.09(2.16 to 7.04) | 4.11(2.2 to 7.04) | 0(0 to 0) | 0.01(0 to 0.01) |
| Syrian Arab Republic | 20.15(14.15 to 26.63) | 19.03(13.33 to 25.27) | 19.95(14.49 to 25.38) | 19.63(14.25 to 25) | 15.86(9.6 to 23.59) | 15.87(9.65 to 23.59) | 0.01(0 to 0.05) | 0.01(0 to 0.03) |
| Taiwan (Province of China) | 15.95(11.71 to 20.71) | 15.81(11.62 to 20.53) | 16.1(11.97 to 20.52) | 16.09(11.96 to 20.5) | 12.08(7.48 to 17.88) | 12.08(7.48 to 17.88) | 0.01(0 to 0.03) | 0.01(0 to 0.02) |
| Tajikistan | 20.39(10.87 to 33.4) | 19.42(10.44 to 31.96) | 16.69(8.9 to 25.52) | 16.41(8.76 to 24.99) | 16.27(7.96 to 27.8) | 16.22(7.92 to 27.71) | 0.41(0.08 to 1.33) | 0.16(0.03 to 0.49) |
| Thailand | 17.83(13.3 to 22.86) | 17.55(13.1 to 22.51) | 16.78(12.08 to 21.31) | 16.73(12.04 to 21.25) | 13.74(8.82 to 19.89) | 13.75(8.85 to 19.89) | 0.01(0 to 0.02) | 0.01(0 to 0.01) |
| Timor-Leste | 7.22(3.71 to 12.41) | 6.91(3.56 to 11.85) | 5.42(2.45 to 9.92) | 5.35(2.42 to 9.77) | 5.67(2.67 to 10.28) | 5.59(2.64 to 10.09) | 0.01(0 to 0.02) | 0.01(0 to 0.01) |
| Togo | 14.21(8.11 to 21.74) | 13.21(7.52 to 20.23) | 9.18(4.33 to 15.29) | 9.13(4.32 to 15.23) | 11.58(6.42 to 18.35) | 11.38(6.33 to 18.12) | 0.07(0.02 to 0.19) | 0.02(0.01 to 0.03) |
| Tokelau | 2.64(0.01 to 7.57) | 2.57(0.01 to 7.4) | 3(0.01 to 9.05) | 2.97(0.01 to 9) | 1.98(0.04 to 6.35) | 2.09(0.1 to 6.39) | 0.02(0 to 0.1) | 0.01(0 to 0.03) |
| Tonga | 6.78(2.19 to 15.63) | 6.55(2.11 to 15.12) | 6.25(1.97 to 14.16) | 6.19(1.95 to 14.01) | 5.2(1.55 to 11.99) | 5.2(1.57 to 11.94) | 0.02(0 to 0.08) | 0.01(0 to 0.01) |
| Trinidad and Tobago | 14.52(4.34 to 27.84) | 14.2(4.22 to 27.14) | 14.81(5.17 to 26.52) | 14.74(5.14 to 26.38) | 11.02(3.16 to 22.81) | 11.07(3.38 to 22.81) | 0(0 to 0.01) | 0.01(0 to 0.01) |
| Tunisia | 20.18(13.39 to 28.25) | 19.72(13.1 to 27.59) | 19.84(13.76 to 26.27) | 19.82(13.74 to 26.23) | 15.56(8.94 to 24.07) | 15.64(9.1 to 24.07) | 0.04(0.01 to 0.11) | 0.01(0 to 0.03) |
| Turkey | 17.7(12.65 to 23.22) | 17.47(12.47 to 22.95) | 17.76(12.96 to 22.87) | 17.72(12.94 to 22.82) | 13.47(8.4 to 20.36) | 13.5(8.46 to 20.36) | 0.05(0.01 to 0.19) | 0.01(0 to 0.02) |
| Turkmenistan | 17.2(9.59 to 27.67) | 16.37(9.18 to 26.21) | 16.93(10.09 to 24.74) | 16.73(9.97 to 24.44) | 13.52(6.62 to 23) | 13.56(6.7 to 23.09) | 0.45(0.05 to 1.04) | 0.11(0.01 to 0.3) |
| Tuvalu | 3.3(1.33 to 6.67) | 3.21(1.3 to 6.48) | 3.03(1.12 to 6.81) | 3(1.11 to 6.75) | 2.5(0.98 to 5.56) | 2.53(1.01 to 5.53) | 0.01(0 to 0.05) | 0(0 to 0.01) |
| Uganda | 10.4(5.83 to 16.23) | 9.27(5.13 to 14.48) | 6.42(2.96 to 11) | 6.36(2.96 to 10.89) | 8.3(4.31 to 13.38) | 8.18(4.11 to 13.34) | 0.04(0.01 to 0.12) | 0.02(0.01 to 0.04) |
| Ukraine | 9.57(5.48 to 14.81) | 9.49(5.43 to 14.68) | 10.08(5.9 to 15.09) | 10.07(5.9 to 15.07) | 7.12(3.72 to 12.36) | 7.14(3.74 to 12.39) | 0.04(0.01 to 0.1) | 0.01(0 to 0.02) |
| United Arab Emirates | 26.77(19.17 to 34.81) | 26.5(18.94 to 34.46) | 24.69(17.57 to 31.52) | 24.67(17.56 to 31.49) | 21.12(13.23 to 30.78) | 21.13(13.25 to 30.78) | 0.01(0 to 0.05) | 0.01(0 to 0.01) |
| United Kingdom | 5.51(3.18 to 8.5) | 5.49(3.17 to 8.45) | 6.11(3.66 to 9.29) | 6.1(3.66 to 9.28) | 4.08(2.13 to 7.06) | 4.1(2.14 to 7.08) | 0(0 to 0) | 0.01(0 to 0.01) |
| United Republic of Tanzania | 8.02(4.73 to 12.25) | 7.15(4.23 to 10.97) | 5.4(2.58 to 9) | 5.34(2.55 to 8.91) | 6.44(3.56 to 10.14) | 6.34(3.4 to 10.11) | 0.03(0.01 to 0.08) | 0.02(0.01 to 0.04) |
| United States of America | 3.54(1.67 to 6.06) | 3.53(1.66 to 6.02) | 3.99(1.91 to 6.75) | 4(1.92 to 6.75) | 2.59(1.15 to 4.9) | 2.62(1.18 to 4.93) | 0.04(0.02 to 0.05) | 0.01(0 to 0.01) |
| United States Virgin Islands | 4.86(2.1 to 8.04) | 4.81(2.08 to 7.98) | 5.41(2.48 to 9.03) | 5.4(2.48 to 9.02) | 3.54(1.53 to 6.7) | 3.58(1.59 to 6.73) | 0.01(0 to 0.03) | 0.01(0 to 0.01) |
| Uruguay | 5.44(2.44 to 9.28) | 5.38(2.42 to 9.19) | 5.92(2.73 to 9.77) | 5.91(2.73 to 9.75) | 3.99(1.77 to 7.62) | 4.02(1.8 to 7.62) | 0(0 to 0.01) | 0.01(0 to 0.01) |
| Uzbekistan | 21.39(12.01 to 32.71) | 20.48(11.54 to 31.41) | 19.47(11.79 to 27.63) | 19.27(11.68 to 27.4) | 16.96(8.85 to 27.55) | 17(8.96 to 27.55) | 1.55(0.41 to 2.93) | 0.76(0.19 to 1.45) |
| Vanuatu | 5.61(1.76 to 12.59) | 5.33(1.68 to 11.98) | 4.28(1.16 to 9.58) | 4.22(1.14 to 9.45) | 4.5(1.31 to 10.06) | 4.43(1.32 to 9.95) | 0.02(0 to 0.07) | 0(0 to 0.01) |
| Venezuela (Bolivarian Republic of) | 14.79(8.59 to 21.66) | 14.52(8.44 to 21.24) | 15.08(9.33 to 20.9) | 15.02(9.29 to 20.81) | 11.19(6.09 to 18.59) | 11.29(6.27 to 18.59) | 0.06(0.01 to 0.15) | 0.01(0 to 0.02) |
| Viet Nam | 12.7(9.32 to 16.96) | 12.51(9.18 to 16.72) | 11.38(7.47 to 15.41) | 11.38(7.49 to 15.4) | 9.8(6.35 to 14.31) | 9.79(6.32 to 14.2) | 0.01(0 to 0.03) | 0.01(0 to 0.01) |
| Yemen | 20.55(12.03 to 30.23) | 19.03(11.2 to 27.92) | 15.56(7.7 to 23.93) | 15.39(7.63 to 23.71) | 16.24(8.66 to 25.94) | 16.11(8.49 to 25.96) | 0.12(0.04 to 0.29) | 0.02(0.01 to 0.04) |
| Zambia | 11.37(6.89 to 17.52) | 10.31(6.23 to 15.86) | 8.39(4.35 to 13.46) | 8.31(4.32 to 13.32) | 9.06(5.3 to 14.24) | 8.95(5.24 to 14.1) | 0.06(0.02 to 0.15) | 0.02(0.01 to 0.05) |
| Zimbabwe | 9.21(5.41 to 14.5) | 8.5(5 to 13.29) | 6.42(3.06 to 10.65) | 6.42(3.06 to 10.63) | 7.33(4.31 to 11.31) | 7.23(4.27 to 11.16) | 0.07(0.02 to 0.18) | 0.04(0.01 to 0.09) |

Abbreviations: DALY disability-adjusted life year; COPD chronic obstructive pulmonary disease; TBL cancer tracheal, bronchus, and lung cancer; LRIs lower respiratory infections; URIs upper respiratory infections.
